# Application of synchrotron through-the-substrate microdiffraction to crystals in polished thin sections

**DOI:** 10.1107/S2052252515007794

**Published:** 2015-06-11

**Authors:** Jordi Rius, Oriol Vallcorba, Carlos Frontera, Inmaculada Peral, Anna Crespi, Carles Miravitlles

**Affiliations:** a Institut de Ciència de Materials de Barcelona, CSIC, Campus de la Universitat Autònoma de Barcelona, Bellaterra, Catalonia 08193, Spain; b Experiments Division (MSPD beamline), ALBA Synchrotron Facility-CELLS, Cerdanyola del Vallès, Barcelona, 08920, Spain

**Keywords:** tts-μXRD, polished thin sections, crystal microvolume, Patterson function direct methods, δ recycling, synchrotron radiation, two-dimensional frame merging, multicrystal merging, structure solution

## Abstract

The synchrotron through-the-substrate X-ray microdiffraction technique (tts-μXRD) is extended to the structural study of microvolumes of crystals embedded in polished thin sections of compact materials [Rius, Labrador, Crespi, Frontera, Vallcorba & Melgarejo (2011 ▸). *J.Synchrotron Rad.* **18**, 891–898]. The resulting tts-μXRD procedure includes some basic steps: (i) collection of a limited number of consecutive two-dimensional patterns (frames) for each randomly oriented crystal microvolume; (ii) refinement of the metric from the one-dimensional diffraction pattern which results from circularly averaging the sum of collected frames; (iii) determination of the reciprocal lattice orientation of each randomly oriented crystal microvolume which allows assigning the *hkl* indices to the spots and, consequently, merging the intensities of the different frames into a single-crystal data set (frame merging); and (iv) merging of the individual crystal data sets (multicrystal merging) to produce an extended data set suitable for structure refinement/solution. Its viability for crystal structure solution by Patterson function direct methods (δ recycling) and for accurate single-crystal least-squares refinements is demonstrated with some representative examples from petrology in which different glass substrate thicknesses have been employed. The section of the crystal microvolume must be at least of the same order of magnitude as the focus of the beam (15 × 15 µm in the provided examples). Thanks to its versatility and experimental simplicity, this method­ology should be useful for disciplines as disparate as petrology, materials science and cultural heritage.

## Abstract 2

The synchrotron through-the-substrate X-ray microdiffraction technique is applied to the structural study of microvolumes of randomly oriented crystals embedded in polished thin sections. The whole procedure is discussed in detail with the help of examples from petrology, and possible future developments are envisaged.

## 1. Introduction   

Polished thin sections of rock with thicknesses between 15 and 30 µm are commonly used in mineralogical and petrologic studies. These sections are fixed on glass substrates and are ideal for microscopic observation and for determining the optical properties of the specimens under plane-polarized transmitted light. The usual glass substrate thickness for a petrologic polished thin section is around 0.15 cm. Further thickness reduction to 0.09 cm is possible, but below this the risk of fracture during conventional sample preparation becomes too high. Microscopic observations can be complemented at selected points of the thin section with scanning electron microscopy or backscattered electron images, with energy dispersive spectrometry or electron microprobe analyses, and even with Raman spectroscopy. One advantage of these local techniques is that they can be applied to in­homo­geneous samples. Very often, the diffraction information at a certain point is also needed to complete the structural characterization. Measurement in transmission mode, *i.e.* by the beam passing through the glass substrate, is very favourable since it leaves the gauge volume largely unchanged during the experiment. In a paper by Rius *et al.* (2011 ▸), the viability of the resulting technique called ‘through-the-substrate microdiffraction’ (tts-μXRD) was demonstrated. That first study was aimed primarily at samples having their components in polycrystalline form. In the present contribution, this technique is extended to thin sections containing crystal microvolumes at least as large as the beam spot (around 15–25 µm diameter). Unlike the polycrystalline case, where the intensities are obtained by circularly averaging the Debye rings, here the intensities of individual reflections are extracted from a reduced number of two-dimensional patterns collected using the rotation method (Arndt & Wonacott, 1977 ▸), *i.e.* by rotating the thin section around a tilt axis normal to the beam direction. The viability of the tts-μXRD technique is demonstrated with three representative petrologic examples. This technique is extremely simple to apply and is primarily intended for fast local crystal structure refinement (in this case the approximate unit cell is known). However, crystal structure solution by Patterson function direct methods (PFDM; Rius, 2012*a* ▸,*b* ▸, 2014*b* ▸) is also possible if enough crystals of the same type are present in the thin section.

**Figure 1:**
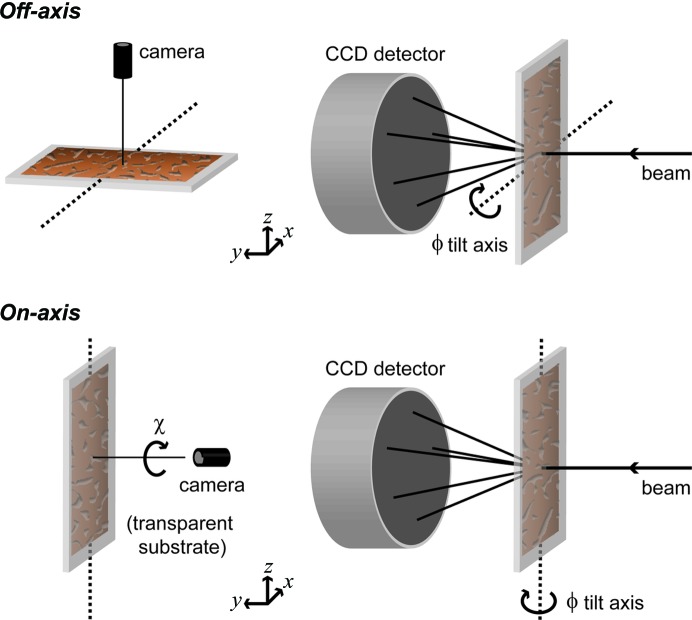
Setups for selecting target points on polished thin sections. Off-axis: (left) the sample visualization system is placed perpendicular to the thin section surface and the target points are searched on the *xy* plane; (right) before data collection the thin section is placed normal to the incoming beam, and the diffraction pattern is collected by rotating the thin section around the ϕ tilt axis (dark part = thin section, light part = glass substrate). On-axis: (left) the visualization system is along the beam axis and the target points are searched on the *xz* plane; (right) before data collection the system is removed, and in this case the diffraction pattern is collected by rotating the thin section around the vertical ϕ tilt axis. The number of collected spots can be increased by an additional collection with the thin section rotated by Δχ = 90° around the axis defined by the beam.

The application of tts-μXRD requires attention to various practical aspects. Since part of the attractiveness of the technique is the easy access to selected points on the thin section, a clear and detailed visualization system is most important. As already mentioned by Rius *et al.* (2011 ▸), one very convenient solution used at the synchrotron beamline BM16 (ESRF, Grenoble, France) is to put the sample visualization system normal to the surface of the thin section, as indicated in Fig. 1 ▸ (off-axis). This enables the user to find the target point by shifting the sample horizontally. Before data collection, the thin section is rotated by 90° to position it normal to the beam (with the substrate placed between the thin section and the incoming beam). A second solution is simply to place the visualization system along the beam axis (on-axis), so that posterior rotation by 90° around the tilt axis is no longer necessary (Fig. 1 ▸). However, the substrate must be transparent, otherwise the target point cannot easily be found. Placing the thin section between the substrate and the incoming beam is not advisable, since intensities diffracted at high angles travel further inside the substrate and will be affected more by absorption. This contributes to an increase in the number of unobserved reflections.

Another important experimental issue is the distribution of the diffraction spots on the two-dimensional pattern. The spots can be produced by means of:

(i) An energy scan (stationary sample). The thin section is placed normal to the incident beam and, since it is kept stationary during the scan, the gauge volume remains unchanged. Modification of the beam energy causes a change in the Ewald sphere radius. Since the region between the Ewald spheres of the upper and lower energy limits of the scan has circular symmetry, the diffraction spots will be uniformly distributed on the two-dimensional frame.

(ii) An angular ϕ-scan (sample rotation). The rotation axis is perpendicular to the beam direction and usually positioned either horizontally (χ = 90°) or vertically (χ = 0°) (Fig.1). During the ϕ-scan, the gauge volume is modified slightly. This setup produces an uneven distribution of spots on the two-dimensional frame, *i.e.* the spot density along the rotation axis direction is lower. This limitation can be overcome by collecting a second two-dimensional diffraction pattern with the sample rotated around the beam axis direction, *e.g.* by applying Δχ = 90° (Helliwell, 1999 ▸).

The angular ϕ-scan mode is more appropriate to monochromatic radiation and its application is the only one treated in the present contribution.

To avoid superposition of spots on the two-dimensional diffraction patterns (frames), the ϕ-scans must be of limited size. Consequently, multiple frames at different offset angles (ϕ_*i*_) are normally collected to increase the number of diffraction spots from the crystal microvolume. In the case of thin sections on substrates, the range of suitable offset angles is obviously restricted by the substrate absorption and noise, and also by the increasing effect of any eventual displacement of the rotation centre.

The strategy for studying crystal microvolumes in thin sections described in this contribution, although very different in its practical aspects and details, bears a resemblance to the strategy used by automated diffraction tomography (ADT) to overcome the missing-wedge limitations in electron diffraction (especially for plate-shaped specimens) (Kolb *et al.*, 2007 ▸, 2008 ▸). The ADT technique has proven to be very effective for structural studies of crystal nanovolumes.

## 2. Description of the overall data collection strategy   

**Figure 2:**
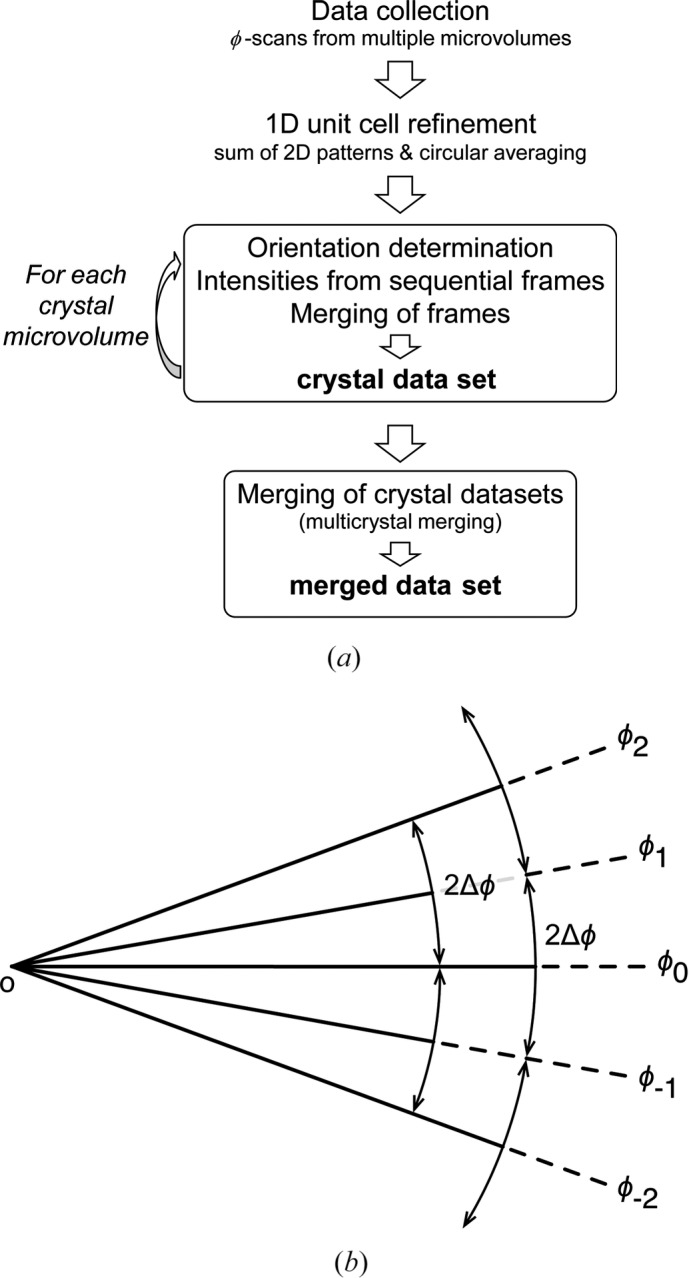
General description of the tts-μXRD technique applied to multiple crystal microvolumes. (*a*) After collecting the data from all crystal microvolumes, refining the global metric, orienting the multiple crystals and merging the sequential ϕ scans of each individual crystal (frame merging), the final data set results from merging the individual data sets (multicrystal merging). To consider possible gauge volume variations, a double scaling process is carried out (the first by scaling each frame during frame merging and the second by scaling each crystal data set during multicrystal merging, as detailed in the text). (*b*) The strategy for data collection for each crystal microvolume. Sequential (partially overlapping) ϕ scans are centred at the offset angles ϕ_*i*_ (= *i*Δϕ) with 2Δϕ widths. The substrate thickness limits the number of ϕ scans.

In a typical tts-μXRD experiment dealing with crystal microvolumes, the X-ray diffraction information is obtained by rotating the thin section with the selected microvolume at the origin (Fig. 2 ▸). Each ϕ scan is defined by its centre ϕ_*i*_ (offset angle) and the corresponding angular increment (Δϕ) (mostly between 5 and 10°). If multiple scans at different offset angles are needed, the corresponding rotation limits for a given offset angle ϕ_*i*_ will be [ϕ_*i*_ − Δϕ, ϕ_*i*_ + Δϕ], which for the particular choice 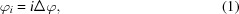
reduce to 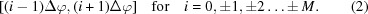
This choice of ϕ_*i*_ ensures that each diffraction spot is measured twice and that a spot lying at the border of one ϕ scan falls within the neighbouring ϕ scan (Fig. 2 ▸ *b*). Data are collected for a limited number of microvolumes of different crystals (*j* = 1 to *N*) of the same compound. According to this schema, each frame is uneqivocally characterized by the (*j*, Δϕ, *i*) triplet.

For small crystal structures, enough diffraction information from the crystal microvolume can be collected in a single broad ϕ scan. For structures with large unit-cell volumes, multiple thinner scans at different offset angles are needed to avoid overlap of diffraction peaks on the two-dimensional pattern. Of all the frames, that with an offset angle equal to zero (zero-frame) is selected for finding the orientation of the crystal which, once known, is used to index the reflections of the frames at non-zero offset angles (off-frames). The data set of each crystal microvolume (crystal data set) is obtained by merging the intensities of the zero- and off-frames (frame merging). In the last step, all crystal data sets are merged to give a more complete data set suitable for accurate crystal structure determination and refinement (multicrystal merging).

### 2.1. Orientation determination of crystal microvolumes   

Before the orientation search, the crystalline compound needs to be identified. To this purpose the diffraction patterns of *N* crystal microvolumes are added, *i.e.* a total of (2*M* + 1)*N* frames. The resulting two-dimensional pattern is then circularly averaged to give a one-dimensional pattern, from which the glass substrate contribution (separately measured) is subtracted. The final one-dimensional pattern is used as follows:

(i) To identify the substance from existing powder diffraction files, *e.g.* PDF-2 or PDF-4 of the International Centre for Diffraction Data. Since there is only one major component, the pattern search should be very reliable even if a few strong intensities are absent. Eventually, information on the chemical composition can be added to restrict the search.

(ii) To index crystal structures by powder diffraction procedures, *e.g.* using indexing programs like *DICVOL* (Boultif & Louër, 2004 ▸; Louër & Boultif, 2014 ▸). The successive dichotomy method used by *DICVOL* is particularly robust against missing reflections at low 2θ angles. The presence of a single phase simplifies the indexing.

(iii) To refine the unit-cell parameters by model-free whole-pattern matching, *e.g.* using *DAJUST* (Vallcorba *et al.*, 2012 ▸). Since no structure model is used, missing reflections or the presence of intensities with preferred orientation do not affect the quality of the whole-pattern refinement. An accurate reciprocal lattice is a requirement for successful determination of the crystal orientation.

As already mentioned, the orientation of the crystal microvolume is determined from the zero-frame information by applying a rotation function variant ROT [see equation (3)] to the background-corrected *y* pixel intensities of the frame. The Ω symbol in equation (3) generically designates the explored angular variables which specify the rotation applied to the (initially arbitrarily oriented) reciprocal lattice, so that *H*(Ω) represents the rotated lattice node *H*. ROT is defined as the sum function measuring the coincidence between the experimental pixel intensities (*y*) of the frame and a delta function with non-zero values (unity) only at *H*(Ω)_proj_, *i.e.* the projection onto the two-dimensional detector of the point where *H*(Ω) crosses the reflection sphere during its rotation around the tilt axis. ROT is calculated with the expression 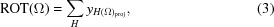
where 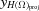 is the intensity measured experimentally at the point *H*(Ω)_proj_. Since ROT is a sum function, the true orientation will be characterized by a positive maximum. In the test examples, the highest ROT values always correspond to the true Ω. It has been implemented in the *DINCO14* code (Rius, 2014*a* ▸).

The portion of reciprocal space which is explored by Ω depends not only on the resolution limit *d*_min_ (the minimum *d* spacing) but also on the semi-aperture Δϕ of the ϕ scan. The number of reflections on a two-dimensional pattern can be estimated roughly with 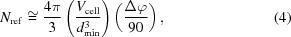
where Δϕ is given in degrees. It is clear that the amount of spot overlap on a two-dimensional frame depends on the number of reflections *N*_ref_. Since for structural studies *d*_min_ is normally fixed around 1 Å, it follows from equation (4) that *N*_ref_ is directly proportional to the product of *V*_cell_ and Δϕ. In other words, to keep overlap to a minimum, Δϕ must be small when *V*_cell_ is large.

All tests indicate that the accuracy of the metric refined from the one-dimensional pattern is enough for the whole tts-μXRD study. However, if the spots of some frames are already indexed then the reciprocal lattice parameters (r.l.p.) and the sample-to-detector separation (OD) can optionally be further refined by minimizing the observed and calculated ρ distances between the spot maxima (*H*) and the centres of the respective two-dimensional patterns, 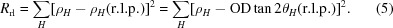
The sample-to-detector distance is periodically updated with 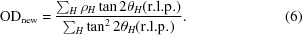
Reflections from at least two differently oriented crystals should be included in the *H* summation. Use of the metric thus optimized should be helpful for two-dimensional patterns with a high spot density.

### 2.2. Estimation of integrated intensities   

The intensity assigned to a given reflection is the intensity of the peak closer than a certain distance to the corresponding calculated reflection position. The peak intensity is estimated by integrating the counts recorded in the detector pixels inside the range defined by the angular azimuthal aperture Δχ of the arc and by Δρ, the width of the radial interval. The integration along the radial direction is carried out first, and the pixel values at the corresponding integration boundaries are used to estimate and remove the background that is considered to be constant in this small region. With these two parameters, different spot types (even those with a certain degree of mosaicity) can be treated. The integrated intensities are corrected for Lorentz and polarization effects (Lipson & Langford, 1999 ▸). For practical purposes, it is interesting to distinguish between completely and partially recorded reflections (complete and partial reflections). Complete ones have the whole diffraction peak within the ϕ-scan interval and hence their intensities are reliable. In contrast, partial reflections are located at the border of the ϕ scan. By representing the angular size of a diffraction peak as ∊, reflections located inside the [−Δϕ, −(Δϕ − ∊)] and [Δϕ − ∊, Δϕ] intervals of a given ϕ scan will have part of the diffraction peak outside the scanned region. Consequently, the measured intensity of a partial reflection will be a fraction of its true value. The above-described data collection strategy circumvents this difficulty by ensuring the participation of each reflection in two consecutive ϕ scans. Unfortunately, for partial reflections lying at the outer borders of the extreme off-frames this no longer holds, so that they are simply left out. (In the test examples ∊ has been taken as 0.5°).

Now, let the angle α between the incident beam and the normal to the substrate be introduced. Since the intensity data are acquired at different α angles, the path length of the primary beam inside the substrate will be modified and thus also the intensity which reaches the thin section. By simple geometric considerations, the additional absorption correction term with respect to the normal incidence is found to be
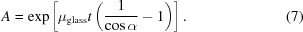
Application of *A* requires the linear absorption coefficient of glass (μ_glass_) to be known. It can be estimated from the intensity ratio, 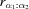, between two glass substrate diffraction patterns, the first measured at α_1_ = 0 and the second at an arbitrary α_2_ value. Since the intensity of each pattern is proportional to the path length inside the glass multiplied by the absorption factor, it holds that 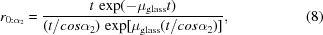
so that 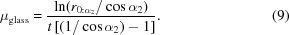
For a glass substrate with *t* = 0.16 cm and for α_2_ = 20°, the experimental ratio is *r*_0:20_ = 0.966 (λ = 0.4246 Å). Introduction of these values into equation (9) gives μ_glass_ = 2.7 cm^−1^, which is of the same order of magnitude as the value of 3.0 cm^−1^ calculated for a common sodium silicate glass [wt%: 37.3 SiO_2_, 10.6 CaO, 13.2 Na_2_O, 1.5 Al_2_O_3_, 1.4Σ K_2_O, SO_3_, Fe_2_O_3_, MgO; mass absorption coefficients from MacGillavry & Rieck (1968 ▸); ρ = 2.5 g cm^−3^]. To illustrate the significance of the relative absorption correction *A*, its dependence on the beam angle of incidence for two μ_glass_ values (corresponding to λ = 0.425 and 0.71 Å) and several thicknesses is given in Table 1 ▸. The limit of α has been set at 40°, due to increasing uncertainties associated with eventual rotation-axis misplacement at higher angles. As is logical, the best condition for low absorption corresponds to the thinnest substrate and the hardest radiation.

### 2.3. Data merging of consecutive frames for a restricted offset interval (frame merging)   

If *I*_*Hj*_ is the intensity (corrected for absorption, polarization and Lorentz effects) of an arbitrary *H* reflection of frame *j*, and if *c*_*j*_ is the scaling factor for this frame (which is inversely proportional to the gauge volume), then *c*_*j*_ *I*_*Hj*_ 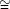 *c*_*i*_ *I*_*Hi*_ must hold for every *i*th frame of the same crystal microvolume. (Notice that the estimation of accurate *c_j_* scaling factors is greatly facilitated by the measurement of consecutive frames with 50% overlap). The best *c*_*j*_ values are those minimizing the *Q*_f_ residual 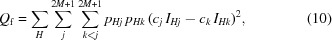
which involves the intensities of the 2*M* + 1 frames. The minimization also includes 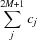 = 2*M* + 1 as a constraint. The *H* sum in equation (10) extends over all reflections in the asymmetric unit (*U*) of reciprocal space, and the value of *p*_*Hj*_ indicates whether the intensity of the *H* reflection (or a symmetry-equivalent one) is present (= 1) or absent (= 0) for frame *j*. The evolution of the refinement is followed at the end of each cycle with the *R*_frame_ figure of merit defined by 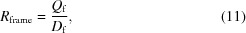
with 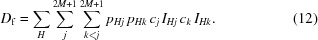
In general, convergence is reached after a few cycles. The result of frame merging is a crystal data set containing the merged intensities of the corresponding crystal microvolume. The merged intensity for a given *H* 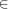 *U* reflection is 
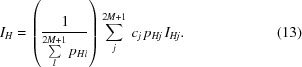
The fact that most reflections are measured twice also allows control of the presence of inconsistent intensities.

### 2.4. Merging of data sets from randomly oriented crystal microvolumes (multicrystal merging)   

In general, the *N* crystal data sets are on slightly different absolute scales. This may be due to small variations in the diffracting volumes, *e.g.* a lack of homogeneity with depth, a lateral change in the thickness of the thin section or even variable primary beam intensities. Similarly to frame merging, the intensities of the *N* data sets can be reduced to a common scale by minimizing 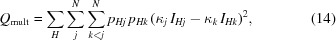
as a function of the κ scale factors of the data sets, together with the 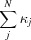 = *N* constraint. Convergence of the refinement is controlled with the *R*_mult_ residual 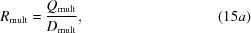


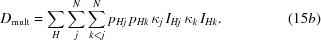
It is calculated at the end of each cycle, and convergence is normally reached after a few cycles. The result of multicrystal merging is a more complete data set including the information from all *N* data sets. The merged intensity for a given *H* 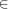 *U* reflection is 
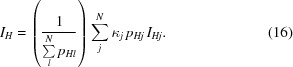

*R*_mult_ is the global figure of merit for multicrystal merging. If its value is abnormally high, the crystal data set(s) responsible must be identified. For instance, an erroneous strong intensity may be the cause. The calculation of an individual *R*_C_ residual for each crystal data set requires the prior definition of a cross residual measuring the discrepancy between two data sets, say *j* and *k*

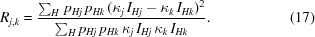
Accordingly, the *R*_C_ residual for the *j*th crystal data set is simply the sum of all cross residuals in which this particular data set participates
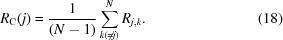
A high value of *R*_C_ identifies a problematic data set, which should be revised. For holohedral Laue groups, multicrystal merging is quite straightforward and facilitated by the increased number of reflections coming from each crystal as a result of frame merging. Once scaled, the intensities of the reflections in the different crystal data sets are merged to give the final list containing all symmetry-independent reflections. Both frame and multicrystal merging have been implemented in the *DMERGE14* code (Rius, 2014*a* ▸) which supplies a file with the basic reflection information, *i.e. hkl* indices, intensity value and associated uncertainty. This file can be processed by a crystal structure determination program like *XLENS* (Rius, 2013 ▸) or by a single-crystal least-squares refinement program like *SHELX97* (Sheldrick, 2008 ▸). Multicrystal merging for merohedral Laue groups is more complicated because such groups have only half (hemihedry) or one quarter (tetrato­hedry) of the symmetry operations of the corresponding lattice symmetry group (crystal system), *e.g.* in the hemihedral Laue group 4/*m*, reflections of type *hkl* and *khl* are no longer equivalent as for 4/*mmm*. In such cases (if Laue symmetry is assumed to be valid for the intensity distribution), the choice between the two sets of indices is arbitrary for the first data set, but once the choice has been made the assignments for the remaining crystal data sets must be consistent with the first choice. Consequently, the scaling procedure has to calculate *R*_mult_ for all possible combinations and select the one with the lowest value. That this situation can be solved was recently demonstrated by Liu & Spence (2014 ▸).

## 3. Practical application and test examples   

### 3.1. Experimental conditions   

Diffraction data were collected at the microdiffraction/high-pressure station of the MSPD beamline (ALBA Synchrotron, Barcelona, Spain) (Fauth *et al.*, 2013 ▸). This endstation is equipped with Kirkpatrick–Baez mirrors providing a monochromatic focused beam of 15 × 15 µm (full width at half-maximum) and a Rayonix SX165 CCD detector (round active area of 165 mm diameter, frame size 2048 × 2048 pixels, 79 µm pixel size, dynamic range 16 bit). The energy used was 29.2 keV (λ = 0.4246 Å), as determined from the Sn absorption *K* edge. The sample-to-detector distance and the beam centre position were calibrated using the *Fit2D* software (Hammersley, 1998 ▸) from LaB_6_ diffraction data measured under exactly the same conditions as the samples. Samples were mounted on an *xyz* stage with a vertical tilt axis. The thin section always faced the detector. The transparent glass substrate allows direct selection of the measurement point with the on-axis visualization system, so that no rotation of the sample was required. In all three examples, the samples were mounted visually normal to the beam. The associated small error (<2°) is not critical, since the correct orientation is found later by the rotation function. The only effect is a small shift in the origin of the offset angle with no practical consequences. Specific data collection conditions for each test example were:

(i) Diopside-(Fe): sample-to-detector distance = 189.95 mm, acquisition time per frame = 4 s, Δϕ (semi-aperture) = 10° (only zero-frames), *N* (number of microvolumes) = 4, *d*_min_ = 1.06 Å, *t* (glass substrate thickness) = 0.16 cm. The microvolume of crystal 4 corresponds to the same thin section as the remaining three crystals but was measured one year later under the same conditions, except for the sample-to-detector distance (184.00 mm).

(ii) Garnet (grossular): sample-to-detector distance = 184.00 mm, acquisition time per frame = 5 s, Δϕ = 7.5°, offset range = −15 to 15°, *N* = 1, *d*_min_ = 1.08 Å, *t* = 0.09 cm.

(iii) Axinite: four and three microvolumes of two different thin sections from the same outcrop were measured. Sample-to-detector distance = 184.00 mm, acquisition time per frame = 3 s, Δϕ = 7.5°, *N* = 7, offset range between −22.5 and 22.5° for four microvolumes and between −15 and 15° for the remaining three, *d*_min_ = 1.08 Å, *t* = 0.09 cm.

In the case of the mineral axinite, a large single crystal (diameter ≃0.3 mm) was also found and its diffraction data (SC data) were measured, thus serving to check the tts data. The SC data were collected on a Bruker APEX CCD diffractometer (graphite-monochromated Mo *K*α radiation) at room temperature [2θ_min_ = 4.537°, 2θ_max_ = 56.659°, number of measured reflections = 4127, *d*_min_ = 0.74 Å, *R*_int_ = 0.022, *R*(σ) = 0.03]. Data collection, data reduction and absorption correction were performed using Bruker *SMART*, *SAINT* and *SADABS* software. Quantitative analyses were carried out on a JEOL JXA-8230 electron microprobe (EMP) at 20 kV, 15 nA and a focus of 5 × 5 µm.

### 3.2. Test examples   

The first and second examples represent limiting situations where either multicrystal merging (diopside) or frame merging (garnet) suffice to characterize the crystal structure fully. The third example (axinite) was selected to illustrate the general case combining both merging modes to produce the final extended data set.

#### 3.2.1. Diopside in a diabase: an example of multicrystal merging   

**Figure 3:**
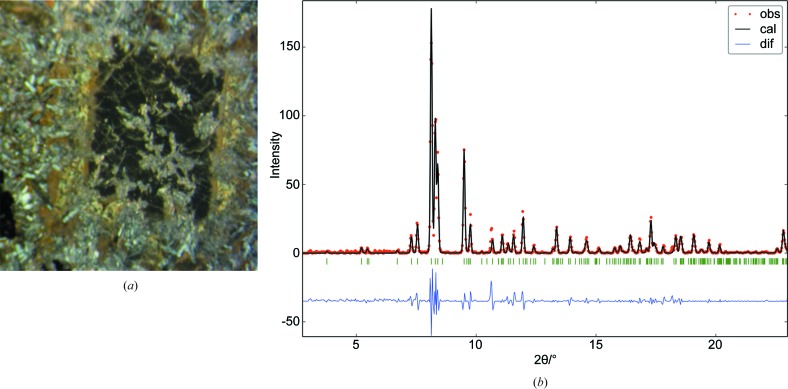
Diopside-(Fe). (*a*) Cross-polarized photomicrograph of one of the studied crystals (black) interpenetrated by the finely grained matrix. The crystal edges are approximately 1 mm long. The size of the beam focus (15 × 15 µm) allows analysis of a homogeneous part of the crystal. (*b*) Model-free whole-pattern refinement, showing the observed pattern (dots), the calculated pattern (line) and their difference (bottom). The observed pattern corresponds to the circular average of the sum of collected two-dimensional patterns.

The purpose of this first example was to confirm that the extracted intensity data allowed accurate crystal structure refinements in spite of the rather thick glass substrate (0.16 cm). The studied polished thin section was cut out of a diabase rock containing aerinite veinlets paved with prehnite at the walls and also including some small unidentified idiomorphic crystals (Fig. 3 ▸ *a*). A total of eleven points distributed over four such crystals were analysed using the EMP to check their similarity. The resulting average cationic composition (normalized to 16 sites) is Si_7.85 (2)_Mg_3.87 (10)_Ca_3.14 (14)_Fe_0.81 (11)_Al_0.33 (4)_. The standard deviations in parentheses measure the variability of the composition among analysed points. To clarify the cationic distribution in the crystal structure, tts-μXRD was applied to microvolumes of these four crystals.

**Figure 4:**
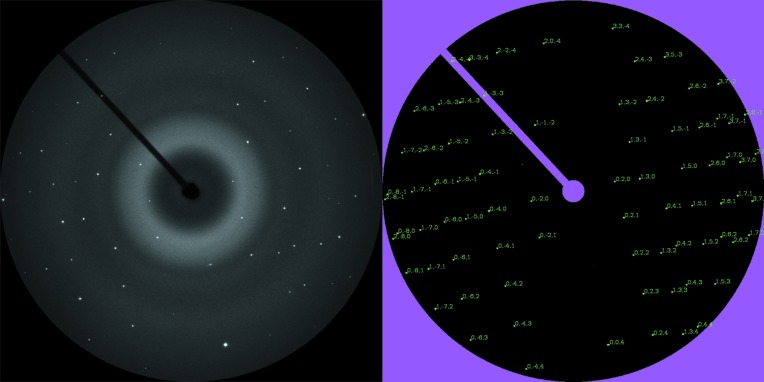
Diopside-(Fe). (left) Zero-frame with ±10° oscillation around the tilt axis of a crystal microvolume in the polished thin section. (right) The corresponding pattern indexed by ROT, the rotation function variant of equation (3) (background removed).

All measured frames were added and circularly averaged to produce a one-dimensional pattern. The experimental pattern of the glass substrate was also circularly averaged and then scaled and subtracted from the one-dimensional pattern of the sample. The difference pattern was indexed with *DICVOL*. The found unit cell fits to a clinopyroxene of the diopside–hedenbergite series (PDF-4 card 04-016-4356). Further model-free whole-pattern refinement with *DAJUST* converged to χ = 0.56 (Fig. 3 ▸ *b*) and supplied the unit-cell parameters for the initial rotation search [*a* = 9.711 (3), *b* = 8.916 (2), *c* = 5.237 (2) Å and β = 106.44 (2)°]. In all four cases, the highest-ranked ROT solution indexed the diffraction spots of the zero-frame (Fig. 4 ▸).

**Figure 5:**
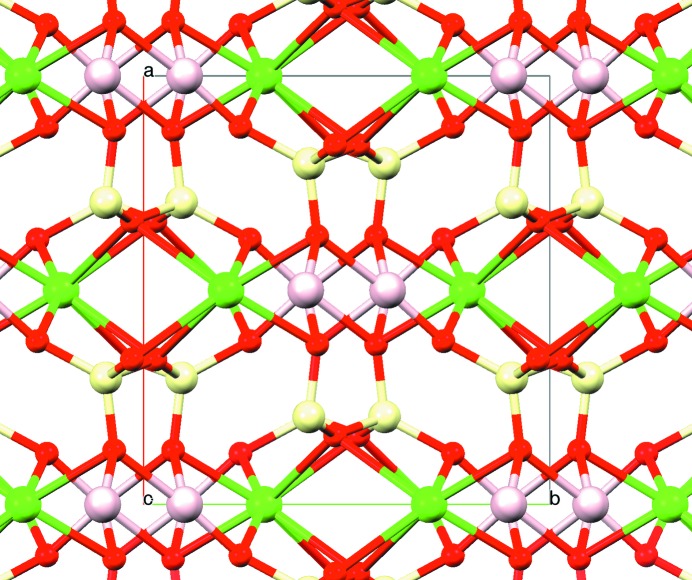
Diopside-(Fe). A perspective view of the unit cell along the *c* direction, as obtained from δ recycling PFDM. The tetrahedrally coordinated Si atoms (*T* site) and the O atoms (small spheres) form the pyroxene chains (upper view). The octahedrally coordinated atoms at *M*1 are mainly Mg and the eightfold coordinated atoms at *M*2 are principally Ca.

To check the accuracy of the unit-cell parameters derived from the one-dimensional pattern, the unit-cell parameters and the sample-to-detector distance were further refined with equations (5) and (6) by introducing five reflections per pattern (*hkl* indices plus *p* coordinates for each spot). The new parameters were *a* = 9.7354 (4), *b* = 8.9109 (6), *c* = 5.2451 (3) Å and β = 106.385 (1)° (*V* = 436.54 Å^3^; sample-to-detector distance = 189.42 mm). A rotation search with this new unit cell gave nearly coincident results for all crystals. The individual values of this second search and of the subsequent multicrystal merging are listed in Table 2 ▸. The merged data set contains 113 independent intensity data. Since the total number of unique reflections at this resolution is 198, it represents a data completeness of 57.07%. Despite being incomplete, application of δ recycling PFDM to this data set solved the structure (three solutions out of 25 trials) (Fig. 5 ▸).

The unit-cell contents of a clinopyroxene of the diopside–hedenbergite series (space group *C*2/*c*) can be expressed by the general formula *X*_4_ *Y*_4_(*T*_8_O_24_), where *T* is the tetrahedrally coordinated site predominantly occupied by Si, *Y* represents the cations at the octahedral *M*1 site (Mg^2+^, Fe^2+^) and *X* represents the large cations sitting on the eightfold coordinated *M*2 site (principally Ca^2+^).

The crystal structure was refined by introducing the final data set in the least-squares refinement program *SHELX97*. The figures of merit for the last refinement were *R*_1_ = 0.061, *wR*_2_ = 0.134 and *S* = 1.033 for all 113 data and 20 parameters, with the corresponding final values listed in Table 3 ▸. Relevant bond lengths are: for the *T* site, *T*—O1 = 1.63 (1), *T*—O2 = 1.59 (1), *T*—O3 = 1.68 (1) and *T*—O3′ = 1.65 (1) Å, with 〈*T*—O〉 = 1.637 Å; for the *M*1 site, *M*1—O1 = 2.04 (1), *M*1—O1′ = 2.15 (1) and *M*1—O2 = 2.03 (1) Å (2×), with 〈*M*1—O〉 = 2.072 Å; and for the *M*2 site, *M*2—O1 = 2.31 (1), *M*2—O2 = 2.28 (1), *M*2—O3 = 2.61 (1) and *M*2—O3′ = 2.74 Å (2×), with 〈*M*2—O〉 = 2.485 Å.

The occupancy of Fe at *M*1was refined separately from the joint Mg and Al occupancy (both unified because of their similar scattering power). The refinement yields 3.64 (Mg+Al) and 0.36 Fe at *M*1 in the unit cell (Table 3 ▸). Regarding the *M*2 site, the refined scattering power suggests full occupancy (four Ca atoms in the unit cell), which contradicts the EMP results (only three atoms). This discrepancy can only be explained if the remaining Fe and Mg reside at *M*2 (the resulting global scattering power is very similar to that of four Ca, *i.e.* 79.6 compared with 80 electrons). Consequently, the respective compositions of sites *T*, *M*1 and *M*2 satisfying both the XRD and EMP requirements are (Si_7.84_Al_0.16_), (Mg_3.48_Fe^2+^_0.36_Al_0.16_) and (Ca_3.14_Fe^2+^_0.46_Mg_0.40_).

#### 3.2.2. Garnet in a metamorphic rock: an example of frame merging   

**Figure 6:**
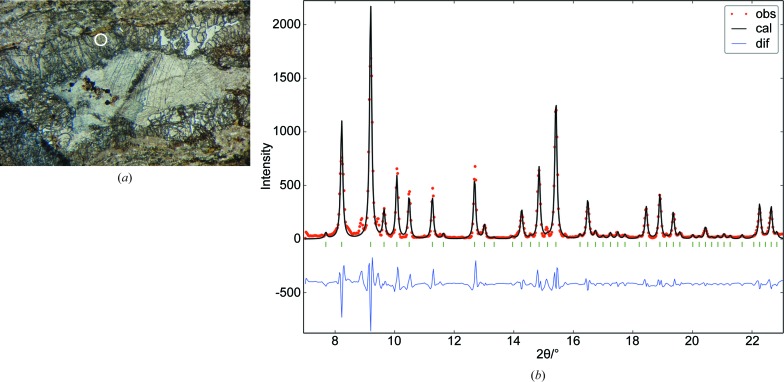
Garnet. (*a*) Photomicrograph of the polished thin section, showing an elongated calcite single crystal limited by two bands (above and below) of cracked garnet (each band is approximately 500 µm thick). The measured microvolume is inside the marked homogeneous block of area 150 × 150 µm. (*b*) Model-free whole-pattern refinement of garnet, showing the observed pattern (dots), the calculated one (line) and their difference (bottom). The observed pattern corresponds to the circular average of the sum of collected two-dimensional patterns.

Owing to the promising results obtained with diopside, which confirmed that the intensities from multicrystal merging are accurate enough for satisfactory single-crystal refinements, the next step was to show the viability of increasing the size of the crystal data set by measuring off-frames. However, this implies a longer beam path through the substrate (increased absorption) and a slight variation in the illuminated volume. To keep the substrate effect to a minimum, the thickness *t* of the glass substrate was reduced from 0.16 to 0.09 cm. The polished thin section was cut out of a contact metamorphic rock from Tibidabo mountain (close to Barcelona city). The measured zone corresponds to a microvolume of a visually homogeneous garnet block (Fig. 6 ▸ *a*).

The selected crystal microvolume was identified as a garnet (grossular) by comparing the corresponding one-dimensional pattern (circular average of the sum of collected two-dimensional frames) with the PDF-4 Minerals database. The general formula (unit-cell content) of a garnet is *A*_24_ *B*_16_(SiO_4_)_24_, with *A* and *B* being, respectively, eightfold and sixfold coordinated sites. The metrics for the orientation search obtained from the model-free whole-pattern refinement (χ_final_ = 2.4) are *a* = 11.8473 (6) Å and *V* = 1662.869 Å^3^ in space group *Ia*3*d* (Fig. 6 ▸ *b*). EMP analysis confirmed that the garnet subspecies is grossular. The cationic composition averaged over three points and scaled to 64 sites is Si_23.88 (50)_Al_10.25 (50)_Ca_23.80 (69)_Fe_5.90 (29)_Mn_0.18 (1)_. Grossular is a typical product of contact metamorphism in impure limestones [a large recrystallized calcite single crystal can be seen in Fig.6(*a*)] and partial replacement of Al by Fe^3+^ occurs quite often (Klein & Hurlbut, 1997*a* ▸). The distribution of Fe in the crystal structure will be investigated by tts-μXRD.

Application of the rotation function with the refined metrics always gave true solutions (only the ten top-ranked ones were checked). The results of frame merging are summarized in Table 4 ▸. Due to the high Laue symmetry of the compound, the frame merging process ended with 44 symmetry-independent reflections, representing a data completeness of 64.29% (*d*_min_ = 1.082 Å) and with an average data redundancy of 3.8 for the observed reflections.

The crystal structure refinement was carried out using *SHELX97*. The final figures of merit were *R*_1_ = 0.034 and *wR*_2_ = 0.087 for all 44 data and ten refined parameters (*S* = 0.86). The refined structural parameters, including the compositions of sites *A* and *B*, are listed in Table 5 ▸. According to the XRD and EMP results, the unit-cell contents of the analysed grossular block must be (Ca_23.82_Mn_0.18_)(Al_10.24_Fe^3+^_5.76_)(Si_24_O_96_). The most relevant bond lengths are: Si—O = 1.645 (5) Å (4×); (*B* site)—O = 1.941 (4) Å (6×); (*A* site)—O = 2.472 (6) Å (4×), 2.318 (4) Å (4×). The refined (*B* site)—O distance coincides with the expected value for the above composition [1.94 Å = 0.64 × 1.90 + 0.36 × 2.01; the respective expected 〈Al—O〉 and 〈Fe^3+^—O〉 bond lengths are 1.90 and 2.01 Å for a coordination number (CN) of 6 (Klein & Hurlbut, 1997*b* ▸)].

#### 3.2.3. Axinite: a general case combining frame and multicrystal merging   

**Figure 7:**
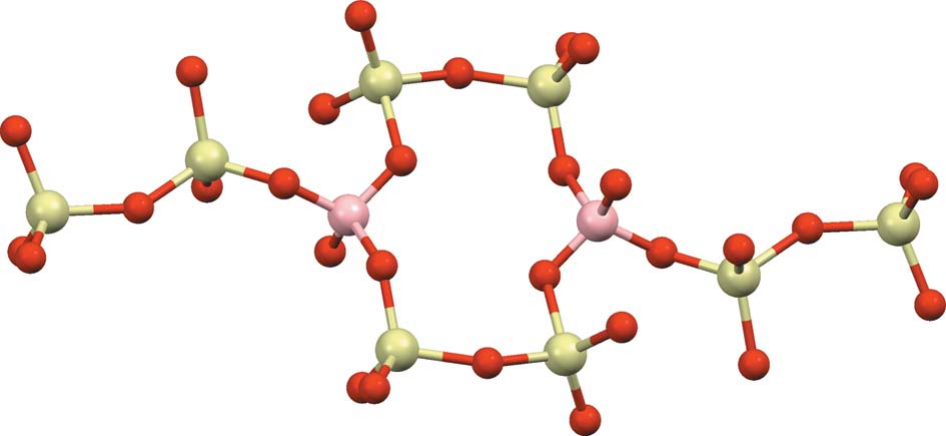
Axinite. A view of the borosilicate anion, [Si_8_B_2_O_30_]^22−^, as determined by δ recycling PFDM from tts data. Besides this anion, axinite also contains Ca^2+^, Al^3+^, Fe^2+^, Mn^2+^ and hydroxyl groups.

**Figure 8:**
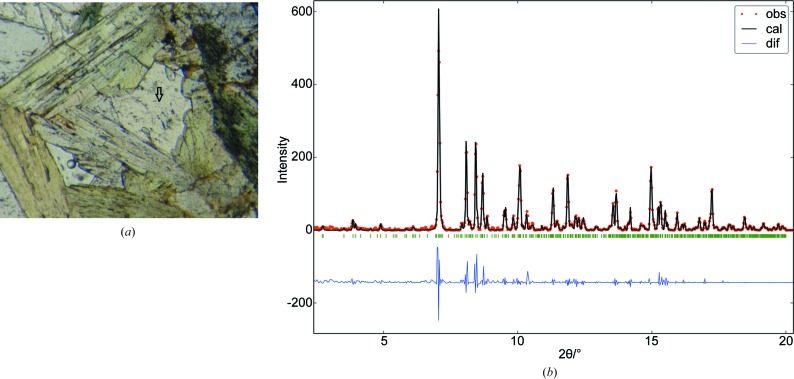
(*a*) Axinite. Photomicrograph showing one representative measured point (arrow) in the middle of the triangle, contoured by green epidote crystals. (*b*) Model-free whole-pattern refinement with the observed pattern (dots), the calculated one (line) and their difference (bottom). The observed pattern corresponds to the circular average of the sum of collected two-dimensional patterns.

Axinite is a triclinic complex silicate with the unit-cell formula Ca_4_ *X*_2_Al_4_[Si_8_B_2_O_30_](OH)_2_ (space group 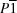). It contains the borosilicate anion [Si_8_B_2_O_30_]^22−^, with *X* being Fe^2+^, Mn^2+^ and even Mg^2+^ (Fig. 7 ▸). The studied specimen comes from an epidote–pyroxene–axinite pneu­mato­litic outcrop close to Pont de Suert (Catalonia, Spain) (Fig. 8 ▸ *a*). EMP analyses (excluding boron) at nine points of several axinite crystals showed a small dispersion. By scaling the Si atomic content to eight sites in the unit cell, the cationic composition is Si_8.00 (5)_Ca_3.94 (13)_Al_3.83 (6)_Fe_1.04 (9)_Mn_0.41 (3)_Mg_0.65 (4)_.

The one-dimensional pattern for the unit-cell refinement was obtained as in the previous examples. The unit-cell parameters were also optimized by model-free whole-pattern refinement and then used for the subsequent orientation search [*a* = 7.1548 (5), *b* = 8.9549 (7), *c* = 9.18633 (6) Å, α = 88.162 (6), β = 77.345 (5), γ = 81.564 (7)°, *V* = 568.1 Å^3^, χ = 1.29] (Fig. 8 ▸ *b*).

The orientations of the crystal microvolumes were determined by applying the rotation function to the corresponding zero-frames. The summary of the orientation determination for each of the seven crystals is given in Table 6 ▸. Once the orientation of the crystal is known, the intensities of the corresponding off-frames can easily be found by applying, consecutively, the different offset rotations. Table 7 ▸ gives the resulting *c* scaling factors (one for each frame), as well as the *R*_frame_ residual measuring the internal consistency of the frame merging process. Finally, multicrystal merging gives the final data set containing 614 unique reflections, which represents a data coverage of 64.7% for *d*_min_ = 1.08 Å (Table 8 ▸). To check if δ recycling can cope with this intensity data set, 25 trials of random phase refinements were computed with *XLENS* (50 cycles per trial). 18 out of the 25 trials were correct solutions. The cycloborosilicate anion shown in Fig. 7 ▸ is the direct output of one such solution.

The relatively large number of observed reflections allows refinement of the crystal structure without restraints. During the refinement, the occupancies of Al1 and Al2 were also refined. Since they were always close to unity they were fixed. For Ca1 and Ca2, the occupancies tend to be slightly lower than unity, which is compatible with the presence of a very small amount of Mg. The final figures of merit supplied by *SHELX97* were *R*_1_ = 0.0648, *wR*_2_ = 0.1542 and *S* = 1.49 for 614 data and 94 parameters. The atomic coordinates for the non-O atoms and the refined occupancies are listed in Table 9 ▸. In parallel, the *SHELX97* refinement with SC data (carried out under identical conditions except for the increased resolution) converged to *R* *1* = 0.054, *wR*_2_ = 0.1722 and *S* = 1.18 for 2439 unique reflections and 94 parameters. Disposal of this independent set of refined parameters allowed checking of the quality of the parameters refined from tts data.

Table 9 ▸ lists the atomic parameters refined from both data sets, showing the good agreement between them [average separation between the positions of pairs of corresponding atoms is 0.008 (2) Å for Si, Ca, Al and *X*, and 0.018 (10) Å for O and B]. This is also reflected in the very similar mean bond lengths between corresponding coordination polyhedra (Table 10 ▸). These values agree with the expected values, namely 〈Ca—O〉 = 2.48 Å (CN = 8), 〈Al—O〉 = 1.90 Å (CN = 6), 〈B—O〉 = 1.47 and 〈Si—O〉 = 1.62 Å (CN = 4) (Klein & Hurlbut, 1997*b* ▸). From the combination of EMP and XRD information, the most probable unit-cell content is (Ca_3.94_Mg_0.06_)(Fe_1.04_Mn_0.41_Mg_0.44_)(Al_3.85_Mg_0.15_)[Si_8_B_2_O_30_](OH)_2_. The composition of *X* is indirectly confirmed by the similarity between the calculated and refined scattering powers, 42.6 and 41.9 (6) electrons, respectively. Further analysis of the irregular coordination polyhedron of *X* is not immediate and will not be pursued here since it is not the purpose of the present contribution.

## 4. Concluding remarks   

The viability of solving and refining crystal structures from two-dimensional patterns of crystal microvolumes collected with the tts-μXRD technique has been demonstrated. In the case of thick glass substrates, frames are preferentially collected at low offset angles. Consequently, a larger number of randomly oriented crystals need to be measured (especially for low symmetries, for which the redundancy of intensities is less). The use of thinner substrates (<1 mm) allows the measurement of data at higher offset angles, thus reducing the number of crystal data sets required. Test calculations also show that, thanks to the proposed data collection strategy in which each reflection is measured twice, the incidence in the refinement of inaccurate intensity data from partially measured reflections at frame boundaries is minimal. Also, refinement of the individual frame scaling factors should largely absorb possible small variations in diffracting volumes (for sections polished to 30 µm thickness, complete homogeneity of the selected microvolume cannot be guaranteed).

Unlike the multicrystal approach to crystal structure and refinement (Vaughan *et al.*, 2004 ▸; Sørensen *et al.*, 2012 ▸) where the polycrystalline sample is measured like a single crystal (*i.e.* by rotating 360°), in tts-μXRD the limited rotation interval and the presence of the glass substrate reduce the ability of the rotation function to discriminate between multiple crystals in the microvolume. A possible solution for microvolumes with only a few crystals is to index a strong spot which is known to correspond to a resolved reflection in 2θ. With this reciprocal lattice direction already fixed, the orientation search reduces to a rotation around this particular direction.

Finally, it is important to distinguish between the requirements imposed on the intensity data by structure solution and refinement methods. Least-squares refinement methods are more sensitive to high detection thresholds, *i.e.* to the presence of a large number of unobserved reflections. To lower the detection threshold, the absorption and background noise should be kept to a minimum. This may be achieved by developing non-conventional sample preparation methods permitting much thinner glass substrates (or even other types of substrate). For Patterson function direct methods, a high detection threshold is not so problematic, since the sole knowledge that an intensity value is weak can be used to advantage during phase refinement. Work is now in progress to incorporate this large amount of information into Patterson-function direct methods more efficiently.

## Supplementary Material

Supporting data for the results shown in the examples (orientation determination, merging and refinements) given in the article.. DOI: 10.1107/S2052252515007794/fc5010sup1.zip

## Figures and Tables

**Table 1:** Relative absorption correction factor *A* as a function of the incident angle of the primary beam for three glass substrate thicknesses *t* and two _glass_ values [3cm^1^ for = 0.425 (Sn *K*) and 12cm^1^ for = 0.71 (Mo *K*)] Variations of *A* greater than 10% are given in italics.

_glass_ (cm^1^)	*t* (cm)	0	10	20	30	40
3	0.015	1.000	1.001	1.003	1.007	1.014
	0.100	1.000	1.005	1.019	1.048	1.096
	0.150	1.000	1.007	1.029	1.072	*1.147*
12	0.015	1.000	1.003	1.012	1.028	1.057
	0.100	1.000	1.019	1.080	*1.204*	*1.443*
	0.150	1.000	1.028	*1.122*	*1.321*	*1.733*

**Table 2:** Diopside-(Fe): application of rotation search to the zero-frames (10 oscillation) of four crystal microvolumes (only the ten top-ranked solutions are considered) *w*/*t* is the ratio of the highest wrong solution to the true one, *N*_spots_ is the number of indexed spots for the best solution, sep is the average angular separation (in ) between the calculated reflection position and the closest peak centre for the best solution, is the average of the values of the indexed reflections (in ), also for the best solution, and , *R* and *N*_I_ are, respectively, the scaling factor, the residual and the number of intensities (after merging symmetry-equivalent intensities). Merging of the data sets of the four crystals gives *R*_mult_ = 0.032 for *d*_min_ = 1.05.

Crystal	*w*/*t*	*N*_spots_	sep			*R*	*N*_I_
1	0.57	53	0.052	1.09	1.028	0.025	37
2	0.61	42	0.061	0.96	1.019	0.025	41
3	0.53	48	0.101	1.23	1.006	0.018	44
4	0.50	63	0.061	0.31	0.944	0.043	56

**Table 3:** Diopside-(Fe): atomic coordinates, occupancies and isotropic *U* values refined from four merged data sets with s.u.s in parentheses The refinement assumes complementary occupancies for Mg and Fe at *M*1 (site code = multiplicity and Wyckoff notation).

Atom or site	Site code	Occupancies and atomic type	*x*/*a*	*y*/*b*	*z*/*c*	*U*_iso_ (^2^)
*T*	8*f*	1 Si	0.2898(6)	0.0924(6)	0.2372(12)	0.013(2)
O1	8*f*	1 O	0.1150(11)	0.0889(12)	0.1404(23)	0.013(2)
O2	8*f*	1 O	0.3648(10)	0.2493(11)	0.3256(24)	0.013(2)
O3	8*f*	1 O	0.3509(10)	0.0196(13)	0.9943(22)	0.013(2)
*M*1	4*e*	0.91(2) Mg + 0.09(2) Fe	0	0.9061(8)	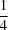	0.013(4)
*M*2	4*e*	1.00(2) Ca	0	0.29520(5)	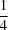	0.021(3)

**Table 4:** Garnet: refined scaling factor *c* for each frame, with the number of extracted intensities (*N*_extracted_) and average angular separation (sep, in ) between the calculated reflection position and the closest peak centre Intensity extraction consists of two-stages. In the first stage, rotation search is applied to the zero-frame (_0_, = 7.5) to orient the reciprocal lattice. In the second stage, the remaining frames are indexed by applying successive offset increments to the oriented lattice.

Frame	_*i*_	*c*	*N*_extracted_	sep
1	15.0	0.933	85	0.17
2	7.5	0.952	68	0.27
3	0	1.081	84	0.13
4	7.5	1.005	78	0.24
5	15.0	1.022	62	0.22
*R*_frame_	0.021			

**Table 5:** Refined atomic coordinates, occupancies and isotropic *U* values from frame-merged intensity data of a single grossular microvolume Site *B* is refined with complementary AlFe occupancies.

Atom or site	Site code	Occupancies and atomic type	*x*/*a*	*y*/*b*	*z*/*c*	*U*_iso_ (^2^)
Si	24*d*	1 Si	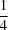	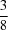	0	0.011(2)
O	96*h*	1 O	0.0466(5)	0.6524(3)	0.0380(4)	0.015(2)
*A*	24*c*	1.02(3) Ca	0	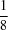	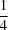	0.015(2)
*B*	16*a*	0.64(2) Al +0.36(2) Fe	0	0	0	0.012(2)

**Table 6:** Axinite: application of the rotation search to the zero-frames (7.5 oscillation) of seven crystal microvolumes: (only the ten top-ranked solutions are considered) *w*/*t* is the ratio of the highest wrong solution to the true one, *N*_spots_ is the number of indexed spots for the best solution, and sep and are as in Table 2 ▸.

Crystal	*w*/*t*	*N*_spots_	sep	
1	0.40	87	0.052	0.24
2	0.38	72	0.044	0.65
3	0.49	79	0.118	0.07
4	0.39	68	0.061	0.16
5	0.39	68	0.064	0.57
6	0.49	76	0.106	0.35
7	0.60	61	0.034	0.48

**Table 7:** Frame merging for axinite: refined scaling factors *c* (zero- and off-frames) with the corresponding *R*_frame_ value for each of the seven crystal microvolumes

		Crystal						
Frame	_*i*_	1	2	3	4	5	6	7
1	22.5	1.070	1.009	0.991	1.057			
2	15.0	1.035	1.026	0.989	0.997	0.992	1.012	0.997
3	7.5	1.016	0.993	0.996	1.000	0.984	1.043	1.004
4	0	1.001	0.987	1.031	0.997	0.963	0.978	1.037
5	7.5	0.997	0.966	1.008	0.974	1.021	0.986	0.978
6	15.0	0.955	0.942	0.945	0.976	1.038	0.973	0.983
7	22.5	0.918	1.072	1.037	0.998			
*R*_frame_		0.023	0.021	0.024	0.040	0.009	0.008	0.008

**Table 8:** Multicrystal merging for axinite (*R*_mult_ = 0.023 for *d*_min_ = 1.08) , *R*_C_ and *N*_I_ are, respectively, the scaling factor, the residual and the number of intensities for each crystal data set.

Crystal	1	2	3	4	5	6	7
	0.995	0.997	1.006	0.985	0.970	0.969	1.073
*R*_C_	0.081	0.060	0.057	0.050	0.072	0.071	0.102
*N*_I_	146	117	134	119	101	95	84

**Table 9:** Axinite: atomic coordinates, occupancies and isotropic *U* values for non-O atoms refined from final tts data (upper value) and from SC data (lower value, in italics) Site *X* normally contains Fe^2+^ and Mn^2+^, but the exact composition depends on the sample origin.

Atom or site	Site code	Occupancy and atomic type	*x*/*a*	*y*/*b*	*z*/*c*	*U*_iso_ (^2^)
Ca1	2*i*	0.993(15) Ca	0.1822(5)	0.9161(4)	0.6013(4)	0.019(1)
		*1.002(6)*	*0.1827(2)*	*0.9162(2)*	*0.6010(1)*	*0.009(0.3)*
Ca2	2*i*	0.982(15) Ca	0.2543(5)	0.3939(4)	0.1517(4)	0.018(1)
		*0.995(6)*	*0.2536(1)*	*0.3947(1)*	*0.1523(1)*	*0.008(0.3)*
*X*	2*i*	0.806(11) Fe	0.7664(4)	0.8875(4)	0.0922(3)	0.020(2)
		*0.796(4)*	*0.7678(1)*	*0.8871(1)*	*0.0916(1)*	*0.009(0.3)*
Al4	2*i*	1 Al	0.0523(7)	0.7468(5)	0.3004(5)	0.012(1)
			*0.0527(2)*	*0.7458(1)*	*0.3006(1)*	*0.003(0.3)*
Al5	2*i*	1 Al	0.3514(7)	0.5797(6)	0.4360(5)	0.015(1)
			*0.3516(2)*	*0.5790(1)*	*0.4363(1)*	*0.004(0.3)*
Si6	2*i*	1 Si	0.6983(7)	0.9889(5)	0.7567(5)	0.018(1)
			*0.6994(2)*	*0.98843(1)*	*0.7566(1)*	*0.005(0.3)*
Si7	2*i*	1 Si	0.2105(7)	0.7673(5)	0.9500(5)	0.015(1)
			*0.2110(2)*	*0.7664(1)*	*0.9498(1)*	*0.005(0.3)*
Si8	2*i*	1 Si	0.3587(6)	0.2306(5)	0.4809(5)	0.015(1)
			*0.3586(2)*	*0.2303(1)*	*0.4812(1)*	*0.005(0.3)*
Si9	2*i*	1 Si	0.7819(7)	0.5223(5)	0.2259(5)	0.015(1)
			*0.7811(2)*	*0.5231(1)*	*0.2254(1)*	*0.004(0.3)*
B10	2*i*	1 B	0.4608(27)	0.7118(23)	0.1342(21)	0.013(1)
			*0.4603(7)*	*0.7132(5)*	*0.1341(5)*	*0.005(0.8)*

**Table 10:** Coordination polyhedra in axinite The asterisk (*) in the first column indicates that this site is filled with Fe, Mn and Mg. For the mean bond lengths, the value in parentheses gives an idea of the dispersion of the individual bond lengths. The agreement between corresponding mean bond lengths from tts and SC data is excellent.

Central cation	Coordination No.	Mean bond length from tts data	Mean bond length from SC data
Ca1	8	2.535(0.300)	2.534(0.304)
Ca2	7	2.484(0.205)	2.479(0.201)
X*	6	2.217(0.264)	2.209(0.257)
Al4	6	1.907(0.042)	1.905(0.052)
Al5	6	1.887(0.039)	1.891(0.029)
Si6	4	1.620(0.022)	1.624(0.023)
Si7	4	1.618(0.036)	1.619(0.029)
Si8	4	1.618(0.025)	1.624(0.017)
Si9	4	1.610(0.034)	1.619(0.022)
B10	4	1.483(0.034)	1.479(0.034)
