# Supplementary material for: Application of synchrotron through-the-substrate microdiffraction to crystals in polished thin sections
Source: IUCrJ. 2015 Jun 11;2(Pt 4):452–63. doi: 10.1107/S2052252515007794 (PMC4491317; doi:10.1107/S2052252515007794)
Supplement: Supplementary file 1 [file m-02-00452-sup1.zip › Suppl_files_Rius/Axinite_merging&scaling&LS/Suppl_Axinite.docx]

**Axinite- Multi-crystal merging.**

**J.Rius ICMAB_CSIC 2014**

CRYSTAL DATA:

=============

AXINITA(PONT DE SUERT) P-1 V=568.30 AX2+MAX2,t=3s, CELLA DAJUST

A= 7.155 B= 8.955 C= 9.186

ALPHA= 88.16 BETA= 77.35 GAMMA= 81.56

VOLUME (A3)= 568.06

(SINT/L)2= 0.005237*H2 + 0.003186*K2 + 0.003112*L2 +

-0.001170*HK +-0.001751*HL + 0.000000*KL

BRAVAIS LATTICE IS P CENTRED AT:

1) 0.000000 0.000000 0.000000

LAUE SYMMETRY OPERATIONS:

R11 R12 R13 R21 R22 R23 R31 R32 R33 T1 T2 T3

1) 1 0 0 0 1 0 0 0 1 0.00 0.00 0.00

TYPE OF RADIATION IS X-RAYS

UNIT CELL CONTENTS

SYMBOL ATOMIC_NUMBER NUMBER IN CELL TYPE SCAT_POWER

CA 20 6 1 20.00

SI 14 8 2 14.00

AL 13 4 3 13.00

O 8 32 4 8.00

B 5 2 5 5.00

H 1 2 6 1.00

DSMIN OF INPUT REFLECTIONS IS: 1.0800 ANGS

RESIDUAL: 0.0596

------------------------------------------------------------------------------

FACTOR DE ESCALA (F"S) N.PATRO NOM_PATRO

0.9954602 1 MAXI_01.HKL

0.9968784 2 MAXI_02.HKL

1.0058788 3 MAXI_03.HKL

0.9851037 4 MAXI_07.HKL

0.9703034 5 NAXI_08.HKL

0.9693254 6 NAXI_10.HKL

1.0732964 7 NAXI_14.HKL

------------------------------------------------------------------------------

MATRIU DE CORRELACIO:

276 140 152 140 103 117 75

140 277 216 75 78 58 56

152 216 265 81 89 63 58

140 75 81 253 133 93 76

103 78 89 133 193 38 44

117 58 63 93 38 221 95

75 56 58 76 44 95 191

------------------------------------------------------------------------------

MATRIU DE RESIDUALS:

0.0000 0.0398 0.0461 0.0571 0.0767 0.0533 0.2144

0.0398 0.0000 0.0435 0.0306 0.0488 0.0793 0.1175

0.0461 0.0435 0.0000 0.0461 0.0397 0.0597 0.1091

0.0571 0.0306 0.0461 0.0000 0.0471 0.0666 0.0497

0.0767 0.0488 0.0397 0.0471 0.0000 0.1328 0.0881

0.0533 0.0793 0.0597 0.0666 0.1328 0.0000 0.0324

0.2144 0.1175 0.1091 0.0497 0.0881 0.0324 0.0000

------------------------------------------------------------------------------

HKL F2AV DF2AV N.CONT F2SCA(IMAG=1), F2SCA(IMAG=2)... FINS NIMAG:

========================================================================

4 0 0 1736.560 41.672 3 0.000 2037.798 1542.477 0.000 1629.406 0.000 0.000

5 0 0 2498.564 49.986 3 0.000 3148.063 2172.732 0.000 2174.896 0.000 0.000

6 0 0 344.212 18.553 3 0.000 433.133 399.031 0.000 200.471 0.000 0.000

-4 1 0 786.237 28.040 1 0.000 0.000 0.000 0.000 786.237 0.000 0.000

1 1 0 125.357 11.196 2 136.492 0.000 114.221 0.000 0.000 0.000 0.000

2 1 0 1023.059 31.985 4 0.000 974.328 926.710 1329.275 861.923 0.000 0.000

3 1 0 1212.433 34.820 4 0.000 1508.061 993.084 1370.363 978.226 0.000 0.000

5 1 0 234.400 15.310 4 0.000 261.390 201.094 284.219 190.896 0.000 0.000

-5 2 0 491.514 22.170 1 0.000 0.000 0.000 0.000 491.514 0.000 0.000

2 2 0 605.675 24.610 2 0.000 448.387 762.962 0.000 0.000 0.000 0.000

3 2 0 786.239 28.040 3 0.000 955.755 742.696 0.000 660.266 0.000 0.000

4 2 0 94.578 9.725 1 0.000 0.000 0.000 94.578 0.000 0.000 0.000

5 2 0 415.082 20.374 4 0.000 401.959 380.930 507.321 370.118 0.000 0.000

0 3 0 274.191 16.559 1 0.000 0.000 0.000 0.000 0.000 0.000 274.191

1 3 0 3735.960 61.123 2 0.000 0.000 0.000 0.000 0.000 3269.310 4202.611

3 3 0 9813.068 99.061 2 0.000 9068.199 10557.938 0.000 0.000 0.000 0.000

4 3 0 1576.727 39.708 4 1882.887 1829.673 1414.182 0.000 1180.166 0.000 0.000

5 3 0 31871.426 178.526 1 0.000 0.000 0.000 0.000 31871.426 0.000 0.000

-1 4 0 3740.834 61.162 1 0.000 0.000 0.000 0.000 0.000 0.000 3740.834

0 4 0 2990.817 54.688 2 0.000 0.000 0.000 0.000 0.000 2981.710 2999.925

1 4 0 2841.741 53.308 2 0.000 0.000 0.000 0.000 0.000 2476.999 3206.483

2 4 0 18704.576 136.765 1 0.000 0.000 0.000 0.000 0.000 18704.576 0.000

3 4 0 433.876 20.830 2 503.507 0.000 364.245 0.000 0.000 0.000 0.000

4 4 0 4418.275 66.470 2 0.000 4646.346 4190.206 0.000 0.000 0.000 0.000

5 4 0 401.682 20.042 3 447.370 380.652 377.024 0.000 0.000 0.000 0.000

6 4 0 106.457 10.318 1 106.457 0.000 0.000 0.000 0.000 0.000 0.000

-1 5 0 1840.230 42.898 1 0.000 0.000 0.000 0.000 0.000 0.000 1840.230

1 5 0 1628.168 40.351 2 0.000 0.000 0.000 0.000 0.000 1566.046 1690.290

2 5 0 178.955 13.377 1 0.000 0.000 0.000 0.000 0.000 178.955 0.000

3 5 0 2180.937 46.701 2 0.000 0.000 2055.617 0.000 0.000 2306.256 0.000

4 5 0 384.836 19.617 2 0.000 387.072 382.599 0.000 0.000 0.000 0.000

5 5 0 307.030 17.522 3 0.000 263.199 238.550 419.342 0.000 0.000 0.000

-2 6 0 1404.683 37.479 1 0.000 0.000 0.000 0.000 0.000 0.000 1404.683

-1 6 0 248.334 15.759 1 0.000 0.000 0.000 0.000 0.000 248.334 0.000

0 6 0 5077.414 71.256 2 0.000 0.000 0.000 0.000 0.000 4439.674 5715.153

2 6 0 4705.469 68.596 2 0.000 0.000 0.000 0.000 0.000 4412.539 4998.400

4 6 0 1058.097 32.528 4 1184.184 928.069 1204.680 0.000 0.000 915.454 0.000

5 6 0 537.501 23.184 2 0.000 506.821 568.182 0.000 0.000 0.000 0.000

-2 7 0 2152.332 46.393 1 0.000 0.000 0.000 0.000 0.000 0.000 2152.332

0 7 0 992.860 31.510 2 0.000 0.000 0.000 0.000 0.000 919.635 1066.086

4 7 0 2049.853 45.275 3 0.000 0.000 2139.465 0.000 0.000 2087.914 1922.181

0 8 0 2877.311 53.641 2 0.000 0.000 0.000 0.000 0.000 2335.336 3419.286

1 8 0 741.338 27.228 1 0.000 0.000 0.000 0.000 0.000 741.338 0.000

2 8 0 5977.428 77.314 2 0.000 0.000 0.000 0.000 0.000 5253.379 6701.477

-2 -8 1 2198.133 46.884 2 0.000 0.000 0.000 0.000 0.000 1799.149 2597.117

0 -8 1 2527.930 50.279 1 0.000 0.000 0.000 0.000 0.000 0.000 2527.930

-3 -7 1 486.300 22.052 3 0.000 445.168 599.467 0.000 0.000 414.266 0.000

-2 -7 1 1378.226 37.124 3 0.000 0.000 1502.076 0.000 0.000 966.643 1665.960

-1 -7 1 1514.271 38.914 2 0.000 0.000 0.000 0.000 0.000 1354.863 1673.679

1 -7 1 4619.267 67.965 2 0.000 0.000 0.000 0.000 0.000 4877.148 4361.386

2 -7 1 1090.796 33.027 1 0.000 0.000 0.000 0.000 0.000 0.000 1090.796

-3 -6 1 841.124 29.002 4 0.000 690.996 892.077 0.000 0.000 994.483 786.942

0 -6 1 6987.303 83.590 2 0.000 0.000 0.000 0.000 0.000 5671.460 8303.146

1 -6 1 2746.465 52.407 2 0.000 0.000 0.000 0.000 0.000 2975.931 2516.998

-3 -5 1 13829.821 117.600 2 0.000 14685.443 12974.200 0.000 0.000 0.000 0.000

-2 -5 1 798.541 28.258 4 0.000 750.075 836.661 0.000 0.000 722.245 885.182

-1 -5 1 10025.488 100.127 2 0.000 0.000 0.000 0.000 0.000 11633.142 8417.836

0 -5 1 310.902 17.632 1 0.000 0.000 0.000 0.000 0.000 310.902 0.000

1 -5 1 9125.069 95.525 2 0.000 0.000 0.000 0.000 0.000 10141.605 8108.533

-5 -4 1 811.675 28.490 4 0.000 790.998 669.533 1028.422 757.748 0.000 0.000

-4 -4 1 529.559 23.012 3 0.000 505.092 482.959 600.628 0.000 0.000 0.000

-2 -4 1 430.616 20.751 2 0.000 389.050 472.183 0.000 0.000 0.000 0.000

0 -4 1 533.653 23.101 2 0.000 0.000 0.000 0.000 0.000 581.833 485.473

1 -4 1 6989.710 83.604 1 0.000 0.000 0.000 0.000 0.000 0.000 6989.710

-6 -3 1 1187.672 34.463 3 0.000 1273.701 908.367 0.000 1380.947 0.000 0.000

-5 -3 1 1014.891 31.857 3 0.000 1053.005 1224.026 0.000 767.643 0.000 0.000

-3 -3 1 172.108 13.119 3 0.000 157.751 176.386 182.188 0.000 0.000 0.000

-2 -3 1 863.676 29.388 3 1057.017 708.208 825.804 0.000 0.000 0.000 0.000

-1 -3 1 4035.377 63.525 4 0.000 3181.047 6160.933 0.000 0.000 3474.291 3325.240

0 -3 1 1330.772 36.480 2 0.000 0.000 0.000 0.000 0.000 1165.498 1496.046

-5 -2 1 1340.243 36.609 4 0.000 1455.510 1324.476 1486.591 1094.396 0.000 0.000

-4 -2 1 841.469 29.008 4 0.000 852.980 842.135 865.186 805.575 0.000 0.000

-3 -2 1 3163.728 56.247 4 0.000 2944.620 3072.165 3493.468 3144.657 0.000 0.000

-2 -2 1 3419.797 58.479 3 0.000 3351.070 3370.097 3538.224 0.000 0.000 0.000

-1 -2 1 1158.588 34.038 3 1298.757 1083.563 1093.444 0.000 0.000 0.000 0.000

0 -2 1 337.109 18.361 4 0.000 360.727 320.839 0.000 0.000 336.994 329.877

6 -2 1 3866.816 62.184 1 0.000 0.000 0.000 0.000 3866.816 0.000 0.000

-6 -1 1 167.884 12.957 2 0.000 168.543 0.000 167.224 0.000 0.000 0.000

-5 -1 1 216.723 14.722 2 0.000 221.650 0.000 211.796 0.000 0.000 0.000

-4 -1 1 224.226 14.974 3 0.000 182.366 278.010 212.301 0.000 0.000 0.000

-3 -1 1 6116.490 78.208 4 0.000 6241.151 6833.057 7131.025 4260.726 0.000 0.000

-2 -1 1 8717.193 93.366 3 0.000 0.000 9760.677 7625.653 8765.250 0.000 0.000

-1 -1 1 1191.214 34.514 4 1196.938 1107.304 1349.154 1111.462 0.000 0.000 0.000

0 -1 1 787.865 28.069 3 0.000 751.357 714.902 0.000 0.000 0.000 897.335

1 -1 1 453.605 21.298 1 0.000 453.605 0.000 0.000 0.000 0.000 0.000

2 -1 1 434.415 20.843 1 0.000 434.415 0.000 0.000 0.000 0.000 0.000

3 -1 1 4712.917 68.651 1 0.000 4712.917 0.000 0.000 0.000 0.000 0.000

4 -1 1 135.411 11.637 1 0.000 135.411 0.000 0.000 0.000 0.000 0.000

5 -1 1 719.978 26.832 2 0.000 765.727 0.000 0.000 674.228 0.000 0.000

6 -1 1 369.572 19.224 1 0.000 369.572 0.000 0.000 0.000 0.000 0.000

-6 0 1 7073.004 84.101 4 0.000 8724.981 5123.311 7595.929 6847.795 0.000 0.000

-5 0 1 390.622 19.764 4 0.000 273.316 382.994 481.003 425.176 0.000 0.000

-2 0 1 14067.169 118.605 1 0.000 0.000 0.000 0.000 14067.169 0.000 0.000

-1 0 1 354.445 18.827 2 0.000 0.000 0.000 329.742 379.147 0.000 0.000

1 0 1 139.159 11.797 3 152.952 106.403 158.123 0.000 0.000 0.000 0.000

2 0 1 198.427 14.086 2 0.000 200.224 0.000 0.000 196.630 0.000 0.000

3 0 1 8292.972 91.066 1 0.000 0.000 0.000 0.000 8292.972 0.000 0.000

4 0 1 160.794 12.680 1 0.000 0.000 160.794 0.000 0.000 0.000 0.000

5 0 1 150.585 12.271 1 0.000 0.000 150.585 0.000 0.000 0.000 0.000

-4 1 1 1704.386 41.284 1 0.000 0.000 0.000 0.000 1704.386 0.000 0.000

-3 1 1 10251.230 101.248 1 0.000 0.000 0.000 0.000 10251.230 0.000 0.000

-2 1 1 4721.811 68.715 1 0.000 0.000 0.000 0.000 4721.811 0.000 0.000

0 1 1 2606.052 51.050 2 0.000 0.000 0.000 2890.987 0.000 0.000 2321.118

2 1 1 2226.298 47.184 3 2139.451 0.000 2669.138 0.000 1870.305 0.000 0.000

3 1 1 794.485 28.187 2 0.000 0.000 912.728 0.000 676.243 0.000 0.000

5 1 1 523.178 22.873 2 0.000 0.000 478.244 0.000 568.113 0.000 0.000

6 1 1 231.073 15.201 1 0.000 0.000 231.073 0.000 0.000 0.000 0.000

-5 2 1 2319.988 48.166 1 0.000 0.000 0.000 0.000 2319.988 0.000 0.000

0 2 1 958.942 30.967 1 0.000 0.000 0.000 0.000 0.000 0.000 958.942

2 2 1 129.645 11.386 2 131.815 0.000 0.000 127.476 0.000 0.000 0.000

3 2 1 1216.796 34.883 2 1139.196 0.000 1294.396 0.000 0.000 0.000 0.000

4 2 1 5210.622 72.185 3 5249.242 4390.589 5992.035 0.000 0.000 0.000 0.000

5 2 1 340.774 18.460 3 0.000 329.682 290.486 402.156 0.000 0.000 0.000

-1 3 1 18912.365 137.522 1 0.000 0.000 0.000 0.000 0.000 0.000 18912.365

0 3 1 1746.973 41.797 2 0.000 0.000 0.000 0.000 0.000 1667.390 1826.556

2 3 1 1022.426 31.975 2 1062.199 0.000 0.000 0.000 0.000 982.653 0.000

4 3 1 872.199 29.533 2 1002.674 0.000 741.725 0.000 0.000 0.000 0.000

6 3 1 351.397 18.746 3 419.891 342.025 292.276 0.000 0.000 0.000 0.000

-1 4 1 756.864 27.511 1 0.000 0.000 0.000 0.000 0.000 0.000 756.864

0 4 1 10327.339 101.624 2 0.000 0.000 0.000 0.000 0.000 12092.340 8562.338

2 4 1 9098.180 95.384 2 0.000 0.000 0.000 0.000 0.000 9641.658 8554.701

3 4 1 551.516 23.484 2 596.715 0.000 0.000 0.000 0.000 506.318 0.000

4 4 1 1567.461 39.591 2 1950.013 0.000 1184.910 0.000 0.000 0.000 0.000

5 4 1 316.513 17.791 3 315.337 348.097 286.104 0.000 0.000 0.000 0.000

-1 5 1 24356.275 156.065 1 0.000 0.000 0.000 0.000 0.000 0.000 24356.275

1 5 1 196.008 14.000 1 0.000 0.000 0.000 0.000 0.000 196.008 0.000

2 5 1 1232.845 35.112 2 0.000 0.000 0.000 0.000 0.000 1093.572 1372.117

-2 6 1 1138.291 33.739 1 0.000 0.000 0.000 0.000 0.000 0.000 1138.291

-1 6 1 4575.283 67.641 1 0.000 0.000 0.000 0.000 0.000 0.000 4575.283

1 6 1 380.413 19.504 1 0.000 0.000 0.000 0.000 0.000 380.413 0.000

2 6 1 3648.427 60.402 2 0.000 0.000 0.000 0.000 0.000 3309.533 3987.320

3 6 1 1536.157 39.194 2 0.000 0.000 0.000 0.000 0.000 1437.031 1635.284

4 6 1 5588.010 74.753 3 6661.977 0.000 5390.676 0.000 0.000 4711.376 0.000

-2 7 1 1991.736 44.629 1 0.000 0.000 0.000 0.000 0.000 0.000 1991.736

0 7 1 1793.474 42.349 2 0.000 0.000 0.000 0.000 0.000 1617.939 1969.008

1 7 1 694.725 26.358 1 0.000 0.000 0.000 0.000 0.000 694.725 0.000

2 7 1 785.265 28.023 2 0.000 0.000 0.000 0.000 0.000 679.278 891.252

3 7 1 579.991 24.083 1 0.000 0.000 0.000 0.000 0.000 579.991 0.000

0 -7 2 467.837 21.630 2 0.000 518.637 0.000 0.000 0.000 417.038 0.000

1 -7 2 2210.498 47.016 3 0.000 1814.349 0.000 0.000 0.000 2383.481 2433.665

-4 -6 2 1013.079 31.829 2 0.000 970.353 1055.805 0.000 0.000 0.000 0.000

-3 -6 2 1633.462 40.416 3 2057.451 0.000 1407.150 0.000 0.000 0.000 1435.786

-2 -6 2 593.872 24.369 4 0.000 625.069 601.763 0.000 0.000 531.179 617.476

-1 -6 2 3380.490 58.142 4 0.000 3455.893 3388.542 0.000 0.000 3469.593 3207.935

1 -6 2 230.454 15.181 1 0.000 0.000 0.000 0.000 0.000 230.454 0.000

2 -6 2 1040.786 32.261 2 0.000 1309.466 0.000 0.000 0.000 0.000 772.105

3 -6 2 1754.405 41.886 1 0.000 1754.405 0.000 0.000 0.000 0.000 0.000

-5 -5 2 2901.876 53.869 4 0.000 3190.348 3298.351 2665.022 2453.783 0.000 0.000

-4 -5 2 418.526 20.458 2 0.000 349.915 487.137 0.000 0.000 0.000 0.000

-3 -5 2 2905.817 53.906 3 3860.299 2014.583 2842.569 0.000 0.000 0.000 0.000

-2 -5 2 502.621 22.419 3 0.000 506.563 502.972 0.000 0.000 0.000 498.329

-1 -5 2 1931.933 43.954 4 0.000 1822.160 2125.866 0.000 0.000 1788.579 1991.126

0 -5 2 222.451 14.915 2 0.000 246.742 0.000 0.000 0.000 198.160 0.000

1 -5 2 3213.725 56.690 3 0.000 3899.540 0.000 0.000 0.000 3362.827 2378.808

3 -5 2 7690.023 87.693 1 0.000 7690.023 0.000 0.000 0.000 0.000 0.000

-5 -4 2 1964.973 44.328 4 0.000 1840.952 1921.970 2135.207 1961.761 0.000 0.000

-4 -4 2 295.864 17.201 1 0.000 295.864 0.000 0.000 0.000 0.000 0.000

-3 -4 2 1234.803 35.140 3 1367.845 959.849 1376.715 0.000 0.000 0.000 0.000

-2 -4 2 331.260 18.201 3 383.752 315.779 294.249 0.000 0.000 0.000 0.000

-1 -4 2 632.719 25.154 4 0.000 709.798 640.687 0.000 0.000 574.758 605.634

0 -4 2 4248.720 65.182 4 0.000 3928.876 3706.134 0.000 0.000 4874.696 4485.176

2 -4 2 7870.501 88.716 1 0.000 7870.501 0.000 0.000 0.000 0.000 0.000

3 -4 2 539.456 23.226 1 0.000 539.456 0.000 0.000 0.000 0.000 0.000

5 -4 2 283.372 16.834 1 0.000 283.372 0.000 0.000 0.000 0.000 0.000

-5 -3 2 243.569 15.607 2 0.000 253.371 0.000 233.767 0.000 0.000 0.000

-4 -3 2 160.618 12.674 3 165.329 174.853 0.000 141.673 0.000 0.000 0.000

-3 -3 2 335.454 18.315 4 428.879 312.738 267.042 333.158 0.000 0.000 0.000

-2 -3 2 550.819 23.470 3 664.703 447.284 540.469 0.000 0.000 0.000 0.000

0 -3 2 2119.607 46.039 4 0.000 1808.754 1725.945 0.000 0.000 2469.294 2474.433

1 -3 2 435.326 20.864 2 0.000 426.485 444.167 0.000 0.000 0.000 0.000

2 -3 2 1709.219 41.343 1 0.000 1709.219 0.000 0.000 0.000 0.000 0.000

4 -3 2 2192.139 46.820 1 0.000 2192.139 0.000 0.000 0.000 0.000 0.000

-5 -2 2 2813.714 53.044 4 0.000 3011.251 2906.423 2796.894 2540.287 0.000 0.000

-4 -2 2 105.864 10.289 1 0.000 0.000 0.000 105.864 0.000 0.000 0.000

-3 -2 2 218.914 14.796 4 0.000 217.903 215.583 193.174 248.996 0.000 0.000

-2 -2 2 1247.012 35.313 4 1287.926 1217.553 1148.677 1333.894 0.000 0.000 0.000

1 -2 2 2105.369 45.884 2 0.000 2056.441 2154.297 0.000 0.000 0.000 0.000

2 -2 2 1815.701 42.611 1 0.000 1815.701 0.000 0.000 0.000 0.000 0.000

3 -2 2 552.653 23.509 1 0.000 552.653 0.000 0.000 0.000 0.000 0.000

4 -2 2 351.455 18.747 1 0.000 351.455 0.000 0.000 0.000 0.000 0.000

5 -2 2 318.254 17.840 1 0.000 318.254 0.000 0.000 0.000 0.000 0.000

-5 -1 2 407.274 20.181 3 0.000 336.440 388.356 497.025 0.000 0.000 0.000

-3 -1 2 275.167 16.588 4 0.000 166.029 309.305 281.502 343.832 0.000 0.000

-1 -1 2 8043.487 89.685 5 7079.589 8302.631 8719.604 7120.254 8995.359 0.000 0.000

0 -1 2 364.920 19.103 4 309.243 375.147 342.026 0.000 0.000 0.000 433.266

1 -1 2 5350.986 73.150 3 6421.495 4804.930 4826.531 0.000 0.000 0.000 0.000

2 -1 2 6259.477 79.117 1 0.000 6259.477 0.000 0.000 0.000 0.000 0.000

3 -1 2 2415.330 49.146 1 0.000 2415.330 0.000 0.000 0.000 0.000 0.000

4 -1 2 7826.676 88.469 1 0.000 7826.676 0.000 0.000 0.000 0.000 0.000

5 -1 2 4897.854 69.985 2 0.000 5020.846 0.000 0.000 4774.863 0.000 0.000

6 -1 2 146.600 12.108 1 0.000 146.600 0.000 0.000 0.000 0.000 0.000

-5 0 2 1627.514 40.342 3 0.000 1410.314 1808.477 0.000 1663.752 0.000 0.000

-4 0 2 5981.830 77.342 3 0.000 8530.541 3797.812 0.000 5617.138 0.000 0.000

-3 0 2 599.831 24.491 2 0.000 0.000 0.000 455.782 743.880 0.000 0.000

-2 0 2 314.346 17.730 2 0.000 0.000 0.000 312.663 316.030 0.000 0.000

-1 0 2 8426.334 91.795 2 0.000 0.000 0.000 8387.197 8465.471 0.000 0.000

0 0 2 100.743 10.037 3 88.501 0.000 0.000 0.000 105.823 0.000 107.905

2 0 2 145.361 12.057 2 0.000 145.945 144.777 0.000 0.000 0.000 0.000

3 0 2 268.408 16.383 2 0.000 331.600 0.000 0.000 205.216 0.000 0.000

4 0 2 465.736 21.581 3 0.000 615.579 462.278 0.000 319.353 0.000 0.000

5 0 2 287.321 16.951 2 0.000 0.000 269.976 0.000 304.666 0.000 0.000

6 0 2 124.572 11.161 1 0.000 0.000 124.572 0.000 0.000 0.000 0.000

-4 1 2 4280.066 65.422 2 0.000 0.000 0.000 4438.540 4121.593 0.000 0.000

-1 1 2 110.095 10.493 1 0.000 0.000 0.000 110.095 0.000 0.000 0.000

1 1 2 119.860 10.948 2 133.876 0.000 0.000 105.845 0.000 0.000 0.000

3 1 2 268.317 16.380 2 0.000 0.000 285.973 0.000 250.662 0.000 0.000

4 1 2 87.894 9.375 1 0.000 0.000 87.894 0.000 0.000 0.000 0.000

5 1 2 9286.505 96.367 1 0.000 0.000 0.000 0.000 9286.505 0.000 0.000

-2 2 2 344.803 18.569 1 0.000 0.000 0.000 344.803 0.000 0.000 0.000

-1 2 2 5201.385 72.121 1 0.000 0.000 0.000 5201.385 0.000 0.000 0.000

0 2 2 691.784 26.302 2 0.000 0.000 0.000 700.902 0.000 0.000 682.666

1 2 2 362.365 19.036 4 316.189 0.000 0.000 314.691 0.000 374.174 444.405

2 2 2 887.520 29.791 3 839.773 0.000 0.000 886.972 0.000 935.815 0.000

3 2 2 927.129 30.449 3 1189.089 0.000 0.000 556.134 1036.165 0.000 0.000

6 2 2 782.189 27.968 4 897.129 808.578 735.188 0.000 687.861 0.000 0.000

-5 3 2 8250.039 90.830 1 0.000 0.000 0.000 0.000 8250.039 0.000 0.000

-4 3 2 881.215 29.685 1 0.000 0.000 0.000 0.000 881.215 0.000 0.000

0 3 2 562.869 23.725 2 0.000 0.000 0.000 576.251 0.000 0.000 549.487

1 3 2 2266.575 47.609 3 0.000 0.000 0.000 2119.233 0.000 2154.174 2526.317

2 3 2 76.363 8.739 1 0.000 0.000 0.000 76.363 0.000 0.000 0.000

3 3 2 81.010 9.001 2 58.020 0.000 0.000 104.001 0.000 0.000 0.000

4 3 2 9282.296 96.345 3 9886.975 0.000 9351.498 0.000 8608.417 0.000 0.000

5 3 2 575.605 23.992 4 625.016 0.000 606.519 640.774 430.110 0.000 0.000

-1 4 2 1308.068 36.167 1 0.000 0.000 0.000 0.000 0.000 0.000 1308.068

0 4 2 2193.102 46.831 2 0.000 0.000 0.000 0.000 0.000 2283.809 2102.394

1 4 2 9499.673 97.466 2 0.000 0.000 0.000 0.000 0.000 10343.824 8655.521

4 4 2 669.962 25.884 3 765.670 0.000 0.000 473.084 0.000 771.132 0.000

5 4 2 2768.167 52.613 3 2475.390 0.000 2862.997 0.000 2966.113 0.000 0.000

0 5 2 513.084 22.651 2 0.000 0.000 0.000 0.000 0.000 616.701 409.466

2 5 2 1050.435 32.410 2 0.000 0.000 0.000 0.000 0.000 984.720 1116.151

3 5 2 598.446 24.463 3 603.463 0.000 0.000 723.659 0.000 468.217 0.000

4 5 2 3185.279 56.438 3 3758.114 0.000 0.000 2974.541 0.000 2823.182 0.000

-2 6 2 2492.138 49.921 1 0.000 0.000 0.000 0.000 0.000 0.000 2492.138

-1 6 2 4489.104 67.001 1 0.000 0.000 0.000 0.000 0.000 0.000 4489.104

0 6 2 7596.685 87.159 2 0.000 0.000 0.000 0.000 0.000 7255.339 7938.030

2 6 2 15497.176 124.488 2 0.000 0.000 0.000 0.000 0.000 14956.544 16037.809

4 6 2 1557.313 39.463 3 1646.646 0.000 0.000 1675.495 0.000 1349.799 0.000

1 7 2 474.588 21.785 1 0.000 0.000 0.000 0.000 0.000 474.588 0.000

1 8 2 580.536 24.094 1 0.000 0.000 0.000 0.000 0.000 580.536 0.000

2 8 2 645.161 25.400 1 0.000 0.000 0.000 0.000 0.000 645.161 0.000

-1 -7 3 270.687 16.453 1 0.000 0.000 0.000 0.000 0.000 270.687 0.000

0 -7 3 744.001 27.276 1 0.000 0.000 744.001 0.000 0.000 0.000 0.000

2 -7 3 256.462 16.014 1 0.000 0.000 0.000 0.000 0.000 256.462 0.000

-3 -6 3 225.380 15.013 1 225.380 0.000 0.000 0.000 0.000 0.000 0.000

-2 -6 3 248.827 15.774 2 258.021 0.000 0.000 0.000 0.000 239.633 0.000

-1 -6 3 1536.473 39.198 3 0.000 0.000 1552.312 0.000 0.000 1378.372 1678.736

1 -6 3 5458.678 73.883 4 0.000 5411.506 5120.700 0.000 0.000 5348.448 5954.059

2 -6 3 7239.893 85.088 2 0.000 5882.969 0.000 0.000 0.000 0.000 8596.816

-4 -5 3 2268.206 47.626 3 0.000 2007.279 2215.521 2581.818 0.000 0.000 0.000

-3 -5 3 1054.769 32.477 3 1086.269 903.195 1174.842 0.000 0.000 0.000 0.000

-2 -5 3 772.421 27.792 4 828.763 713.475 718.332 0.000 0.000 0.000 829.115

-1 -5 3 12218.140 110.536 4 0.000 10160.944 13407.287 0.000 0.000 12890.513 12413.818

2 -5 3 1158.742 34.040 1 0.000 1158.742 0.000 0.000 0.000 0.000 0.000

3 -5 3 4200.125 64.808 1 0.000 4200.125 0.000 0.000 0.000 0.000 0.000

-5 -4 3 164.060 12.809 1 164.060 0.000 0.000 0.000 0.000 0.000 0.000

-4 -4 3 1134.663 33.685 5 1152.335 1050.729 1113.872 1133.733 1222.646 0.000 0.000

-2 -4 3 138.296 11.760 1 138.296 0.000 0.000 0.000 0.000 0.000 0.000

-1 -4 3 477.484 21.851 4 471.609 466.802 460.628 0.000 0.000 0.000 510.897

0 -4 3 3737.138 61.132 4 0.000 3424.370 3554.375 0.000 0.000 3688.978 4280.829

1 -4 3 363.582 19.068 2 0.000 356.703 0.000 0.000 0.000 370.462 0.000

2 -4 3 1506.543 38.814 2 0.000 1387.288 1625.798 0.000 0.000 0.000 0.000

5 -4 3 265.326 16.289 1 0.000 265.326 0.000 0.000 0.000 0.000 0.000

-5 -3 3 1458.471 38.190 5 1399.466 1195.014 1348.790 1754.808 1594.279 0.000 0.000

-4 -3 3 1109.664 33.312 5 1180.944 813.199 1106.202 1246.982 1200.991 0.000 0.000

-3 -3 3 4928.619 70.204 5 5897.594 3852.375 4990.128 5253.992 4649.005 0.000 0.000

-2 -3 3 3945.325 62.812 3 4891.948 3126.131 3817.896 0.000 0.000 0.000 0.000

-1 -3 3 254.898 15.966 4 192.322 251.353 209.856 0.000 0.000 0.000 366.060

1 -3 3 558.805 23.639 3 0.000 400.895 415.644 0.000 0.000 859.877 0.000

2 -3 3 218.490 14.781 1 0.000 218.490 0.000 0.000 0.000 0.000 0.000

4 -3 3 3794.727 61.601 1 0.000 3794.727 0.000 0.000 0.000 0.000 0.000

-5 -2 3 327.976 18.110 1 0.000 0.000 0.000 327.976 0.000 0.000 0.000

-4 -2 3 8552.316 92.479 4 0.000 8255.000 7399.994 10577.874 7976.396 0.000 0.000

-3 -2 3 216.198 14.704 2 189.537 0.000 0.000 242.860 0.000 0.000 0.000

-2 -2 3 13440.441 115.933 4 10445.250 13534.195 13720.487 0.000 16061.834 0.000 0.000

-1 -2 3 3139.573 56.032 3 3120.374 2923.432 3374.913 0.000 0.000 0.000 0.000

0 -2 3 1138.100 33.736 4 1068.938 1057.517 1089.265 0.000 0.000 0.000 1336.683

1 -2 3 2597.548 50.966 3 3096.730 2608.041 2087.874 0.000 0.000 0.000 0.000

2 -2 3 239.670 15.481 2 0.000 261.390 217.950 0.000 0.000 0.000 0.000

4 -2 3 11056.705 105.151 1 0.000 11056.705 0.000 0.000 0.000 0.000 0.000

5 -2 3 396.384 19.909 1 0.000 396.384 0.000 0.000 0.000 0.000 0.000

6 -2 3 162.650 12.753 1 0.000 162.650 0.000 0.000 0.000 0.000 0.000

-4 -1 3 333.283 18.256 3 0.000 296.699 0.000 387.007 316.142 0.000 0.000

-3 -1 3 523.575 22.882 4 0.000 416.319 657.847 373.576 646.558 0.000 0.000

-2 -1 3 4402.596 66.352 5 4682.067 4194.420 4064.622 4156.388 4915.484 0.000 0.000

0 -1 3 5805.158 76.192 4 8514.303 7786.379 6595.073 0.000 0.000 0.000 324.877

1 -1 3 13848.679 117.680 2 15661.266 12036.092 0.000 0.000 0.000 0.000 0.000

2 -1 3 252.921 15.903 2 0.000 247.855 257.987 0.000 0.000 0.000 0.000

3 -1 3 5551.476 74.508 2 0.000 5016.941 6086.010 0.000 0.000 0.000 0.000

4 -1 3 2769.572 52.627 2 0.000 2340.718 3198.426 0.000 0.000 0.000 0.000

5 -1 3 1005.377 31.708 2 0.000 852.413 1158.340 0.000 0.000 0.000 0.000

-5 0 3 303.040 17.408 2 0.000 271.288 334.792 0.000 0.000 0.000 0.000

-3 0 3 1018.802 31.919 3 0.000 0.000 1074.564 932.175 1049.666 0.000 0.000

-1 0 3 762.103 27.606 3 666.210 0.000 0.000 712.654 907.444 0.000 0.000

0 0 3 6031.530 77.663 4 5573.923 0.000 0.000 4962.174 5965.385 0.000 7624.639

1 0 3 13200.169 114.892 3 15079.881 0.000 0.000 14046.208 0.000 10474.419 0.000

2 0 3 8139.232 90.218 3 8692.167 7658.581 0.000 0.000 0.000 8066.950 0.000

3 0 3 4069.058 63.789 2 3891.871 4246.245 0.000 0.000 0.000 0.000 0.000

4 0 3 3679.883 60.662 3 3185.052 3842.677 0.000 0.000 4011.919 0.000 0.000

5 0 3 438.924 20.951 2 0.000 511.760 0.000 0.000 366.088 0.000 0.000

-5 1 3 940.683 30.671 2 0.000 0.000 0.000 876.958 1004.408 0.000 0.000

-3 1 3 6439.778 80.248 2 0.000 0.000 0.000 5727.348 7152.207 0.000 0.000

-2 1 3 716.784 26.773 2 0.000 0.000 0.000 786.436 647.132 0.000 0.000

-1 1 3 16222.604 127.368 1 0.000 0.000 0.000 0.000 16222.604 0.000 0.000

1 1 3 15294.646 123.672 1 0.000 0.000 0.000 15294.646 0.000 0.000 0.000

3 1 3 17284.416 131.470 1 17284.416 0.000 0.000 0.000 0.000 0.000 0.000

6 1 3 1932.905 43.965 1 0.000 0.000 0.000 0.000 1932.905 0.000 0.000

-5 2 3 748.372 27.356 2 0.000 0.000 0.000 694.954 801.791 0.000 0.000

-1 2 3 177.948 13.340 1 0.000 0.000 0.000 177.948 0.000 0.000 0.000

0 2 3 2665.788 51.631 2 0.000 0.000 0.000 2697.435 0.000 0.000 2634.141

1 2 3 668.427 25.854 4 570.346 0.000 0.000 666.549 0.000 723.852 712.963

2 2 3 925.497 30.422 3 1070.107 0.000 0.000 837.374 0.000 869.010 0.000

3 2 3 2247.595 47.409 4 2119.623 0.000 0.000 1600.122 3406.268 1864.366 0.000

4 2 3 613.371 24.766 3 631.824 0.000 0.000 549.700 658.590 0.000 0.000

5 2 3 7370.001 85.849 1 0.000 0.000 0.000 0.000 7370.001 0.000 0.000

6 2 3 3270.157 57.185 3 2839.640 0.000 3929.163 0.000 3041.667 0.000 0.000

-3 3 3 2546.142 50.459 2 0.000 0.000 0.000 2601.915 2490.369 0.000 0.000

-1 3 3 3264.167 57.133 2 0.000 0.000 0.000 3328.485 0.000 0.000 3199.848

2 3 3 260.516 16.141 2 250.322 0.000 0.000 270.711 0.000 0.000 0.000

4 3 3 470.043 21.680 4 431.822 0.000 0.000 491.959 513.290 443.102 0.000

5 3 3 214.030 14.630 3 0.000 0.000 233.248 187.827 221.014 0.000 0.000

6 3 3 2607.723 51.066 4 2400.554 0.000 3400.755 2620.586 2008.996 0.000 0.000

-4 4 3 826.447 28.748 1 0.000 0.000 0.000 826.447 0.000 0.000 0.000

-3 4 3 1078.933 32.847 1 0.000 0.000 0.000 1078.933 0.000 0.000 0.000

-1 4 3 2755.828 52.496 2 0.000 0.000 0.000 2874.140 0.000 0.000 2637.516

0 4 3 1618.680 40.233 3 0.000 0.000 0.000 1750.276 0.000 1348.521 1757.242

2 4 3 1949.358 44.152 4 1819.268 0.000 0.000 1559.577 0.000 2283.452 2135.133

3 4 3 896.693 29.945 3 919.583 0.000 0.000 982.055 0.000 788.440 0.000

4 4 3 12354.685 111.152 3 15398.478 0.000 0.000 9584.251 0.000 12081.327 0.000

5 4 3 369.546 19.224 3 394.385 0.000 0.000 419.371 294.884 0.000 0.000

6 4 3 3416.490 58.451 3 3393.120 0.000 3749.408 0.000 3106.941 0.000 0.000

0 5 3 5324.225 72.967 3 0.000 0.000 0.000 5559.667 0.000 5437.417 4975.591

1 5 3 396.257 19.906 2 0.000 0.000 0.000 380.156 0.000 412.359 0.000

2 5 3 2066.139 45.455 3 0.000 0.000 0.000 2079.145 0.000 1978.508 2140.766

4 5 3 169.747 13.029 1 0.000 0.000 0.000 169.747 0.000 0.000 0.000

5 5 3 3150.626 56.130 2 3448.078 0.000 0.000 0.000 0.000 2853.174 0.000

-2 6 3 8246.734 90.812 1 0.000 0.000 0.000 0.000 0.000 0.000 8246.734

-1 6 3 2251.850 47.454 1 0.000 0.000 0.000 0.000 0.000 0.000 2251.850

2 6 3 5877.162 76.663 3 0.000 0.000 0.000 6025.182 0.000 5743.433 5862.869

3 6 3 2222.052 47.139 4 2096.514 0.000 0.000 2305.905 0.000 2416.884 2068.906

4 6 3 2009.645 44.829 3 2268.650 0.000 0.000 1949.496 0.000 1810.791 0.000

5 6 3 520.023 22.804 2 485.105 0.000 0.000 554.940 0.000 0.000 0.000

-1 7 3 3012.343 54.885 1 0.000 0.000 0.000 0.000 0.000 0.000 3012.343

1 7 3 588.015 24.249 1 0.000 0.000 0.000 0.000 0.000 588.015 0.000

3 7 3 966.812 31.094 2 0.000 0.000 0.000 995.301 0.000 938.323 0.000

4 7 3 376.905 19.414 1 0.000 0.000 0.000 376.905 0.000 0.000 0.000

0 -7 4 2797.707 52.893 2 0.000 2822.943 2772.472 0.000 0.000 0.000 0.000

1 -7 4 5600.959 74.840 3 0.000 6454.274 0.000 0.000 0.000 4728.270 5620.334

-3 -6 4 4302.833 65.596 4 4200.995 3503.941 4803.189 0.000 0.000 0.000 4703.209

-2 -6 4 6766.472 82.259 4 5707.086 5463.251 8516.659 0.000 0.000 0.000 7378.890

-1 -6 4 1346.148 36.690 5 1127.770 1273.422 1531.418 0.000 0.000 1370.855 1427.273

0 -6 4 431.010 20.761 1 0.000 0.000 0.000 0.000 0.000 431.010 0.000

2 -6 4 285.035 16.883 1 0.000 0.000 0.000 0.000 0.000 285.035 0.000

-3 -5 4 1162.079 34.089 3 1346.094 951.561 1188.583 0.000 0.000 0.000 0.000

-2 -5 4 936.382 30.600 4 810.263 963.109 879.197 0.000 0.000 0.000 1092.962

-1 -5 4 25202.859 158.754 3 0.000 26397.678 26996.615 0.000 0.000 0.000 22214.289

0 -5 4 1415.298 37.620 5 1077.153 1358.161 1295.640 0.000 0.000 1613.843 1731.692

1 -5 4 4439.868 66.632 2 0.000 4526.318 4353.417 0.000 0.000 0.000 0.000

2 -5 4 3268.344 57.169 3 0.000 2535.188 2894.504 0.000 0.000 4375.341 0.000

3 -5 4 2013.406 44.871 2 0.000 2030.970 1995.841 0.000 0.000 0.000 0.000

-4 -4 4 5803.716 76.182 5 4295.917 6028.167 6004.460 6006.851 6683.186 0.000 0.000

-3 -4 4 683.240 26.139 4 732.999 646.306 612.954 740.700 0.000 0.000 0.000

-2 -4 4 4327.387 65.783 4 3740.296 3814.434 4847.506 0.000 0.000 0.000 4907.314

0 -4 4 10567.900 102.800 4 11211.574 9525.918 10144.652 0.000 0.000 0.000 11389.457

1 -4 4 441.234 21.006 3 0.000 425.740 403.240 0.000 0.000 0.000 494.723

2 -4 4 451.113 21.239 2 0.000 438.967 463.259 0.000 0.000 0.000 0.000

3 -4 4 230.713 15.189 1 0.000 230.713 0.000 0.000 0.000 0.000 0.000

-4 -3 4 419.294 20.477 5 369.879 368.081 434.838 435.538 488.134 0.000 0.000

-3 -3 4 1621.952 40.273 5 1632.813 1730.714 1404.550 1625.954 1715.731 0.000 0.000

-2 -3 4 185.920 13.635 1 185.920 0.000 0.000 0.000 0.000 0.000 0.000

-1 -3 4 222.249 14.908 3 205.194 258.588 0.000 0.000 0.000 0.000 202.965

1 -3 4 771.303 27.772 3 953.691 605.452 754.766 0.000 0.000 0.000 0.000

2 -3 4 18446.508 135.818 2 0.000 19693.500 17199.514 0.000 0.000 0.000 0.000

3 -3 4 9046.460 95.113 2 0.000 8798.670 9294.251 0.000 0.000 0.000 0.000

4 -3 4 4527.374 67.286 2 0.000 4562.501 4492.246 0.000 0.000 0.000 0.000

5 -3 4 710.970 26.664 2 0.000 736.421 685.520 0.000 0.000 0.000 0.000

-4 -2 4 244.098 15.624 1 244.098 0.000 0.000 0.000 0.000 0.000 0.000

-3 -2 4 184.508 13.583 1 0.000 0.000 0.000 184.508 0.000 0.000 0.000

-2 -2 4 869.702 29.491 5 932.238 651.861 982.400 766.765 1015.245 0.000 0.000

0 -2 4 6461.933 80.386 4 4930.694 5449.070 6963.112 0.000 0.000 0.000 8504.855

1 -2 4 418.672 20.461 4 452.018 380.652 410.575 0.000 0.000 431.442 0.000

3 -2 4 1792.742 42.341 3 1821.587 1744.974 1811.664 0.000 0.000 0.000 0.000

4 -2 4 16052.102 126.697 1 0.000 16052.102 0.000 0.000 0.000 0.000 0.000

5 -2 4 1756.257 41.908 2 0.000 1695.902 1816.612 0.000 0.000 0.000 0.000

-4 -1 4 191.330 13.832 1 0.000 0.000 0.000 191.330 0.000 0.000 0.000

0 -1 4 9812.350 99.057 4 10797.084 11284.805 8554.146 8613.366 0.000 0.000 0.000

2 -1 4 1272.067 35.666 4 1403.043 1102.683 1312.729 0.000 0.000 1269.811 0.000

3 -1 4 3313.563 57.564 3 3471.543 2972.047 3497.097 0.000 0.000 0.000 0.000

4 -1 4 2009.173 44.824 3 2506.446 1553.128 1967.946 0.000 0.000 0.000 0.000

5 -1 4 4335.964 65.848 3 4658.918 4031.303 4317.671 0.000 0.000 0.000 0.000

-4 0 4 282.511 16.808 1 0.000 0.000 0.000 282.511 0.000 0.000 0.000

-3 0 4 7499.125 86.597 3 0.000 0.000 5873.514 8337.638 8286.221 0.000 0.000

-2 0 4 15826.335 125.803 3 0.000 0.000 17390.055 14219.449 15869.498 0.000 0.000

-1 0 4 3500.599 59.166 3 3194.843 0.000 0.000 3950.705 3356.247 0.000 0.000

0 0 4 4550.521 67.458 5 3989.132 0.000 4711.390 4286.542 3268.077 0.000 6497.464

1 0 4 1534.679 39.175 6 1855.130 1618.379 1546.200 1326.402 1088.050 1773.912 0.000

3 0 4 472.816 21.744 4 507.788 452.690 544.850 0.000 0.000 385.937 0.000

4 0 4 1582.947 39.786 3 1333.509 975.809 2439.522 0.000 0.000 0.000 0.000

5 0 4 195.553 13.984 1 0.000 195.553 0.000 0.000 0.000 0.000 0.000

6 0 4 170.457 13.056 1 0.000 0.000 0.000 0.000 170.457 0.000 0.000

-3 1 4 11788.783 108.576 2 0.000 0.000 0.000 13339.629 10237.937 0.000 0.000

-1 1 4 219.376 14.811 2 0.000 0.000 0.000 246.062 0.000 0.000 192.689

1 1 4 593.232 24.356 4 593.514 0.000 0.000 484.467 672.242 622.705 0.000

2 1 4 778.962 27.910 4 958.815 0.000 0.000 612.710 900.430 643.893 0.000

3 1 4 599.161 24.478 2 611.886 0.000 0.000 0.000 0.000 586.437 0.000

4 1 4 222.674 14.922 2 172.523 0.000 0.000 0.000 272.825 0.000 0.000

-3 2 4 312.478 17.677 1 0.000 0.000 0.000 312.478 0.000 0.000 0.000

-2 2 4 1556.132 39.448 2 0.000 0.000 0.000 1617.104 1495.159 0.000 0.000

-1 2 4 8765.670 93.625 3 0.000 0.000 0.000 10013.317 8406.910 0.000 7876.780

1 2 4 359.635 18.964 5 381.364 0.000 0.000 384.280 307.904 373.253 351.372

2 2 4 801.542 28.312 4 804.931 0.000 0.000 613.496 931.744 855.996 0.000

4 2 4 3206.655 56.627 3 3434.482 0.000 0.000 3096.621 0.000 3088.861 0.000

5 2 4 450.541 21.226 1 450.541 0.000 0.000 0.000 0.000 0.000 0.000

6 2 4 1086.756 32.966 1 0.000 0.000 1086.756 0.000 0.000 0.000 0.000

0 3 4 5862.059 76.564 2 0.000 0.000 0.000 6257.513 0.000 0.000 5466.604

1 3 4 313.440 17.704 3 290.821 0.000 0.000 319.485 0.000 330.013 0.000

2 3 4 23681.066 153.887 2 21881.203 0.000 0.000 0.000 0.000 25480.930 0.000

3 3 4 6507.083 80.666 3 6739.954 0.000 0.000 7351.478 0.000 5429.816 0.000

4 3 4 9484.517 97.388 3 8868.683 0.000 0.000 12145.302 0.000 7439.565 0.000

5 3 4 910.358 30.172 4 880.173 0.000 0.000 721.175 1024.537 1015.548 0.000

6 3 4 1196.268 34.587 4 1054.916 0.000 1456.940 1026.355 1246.860 0.000 0.000

-3 4 4 3646.326 60.385 2 0.000 0.000 0.000 3240.419 4052.233 0.000 0.000

-2 4 4 4756.234 68.965 2 0.000 0.000 0.000 5193.175 0.000 0.000 4319.293

-1 4 4 1030.802 32.106 2 0.000 0.000 0.000 1078.021 0.000 0.000 983.582

0 4 4 40531.605 201.325 1 0.000 0.000 0.000 0.000 0.000 0.000 40531.605

1 4 4 826.030 28.741 3 0.000 0.000 0.000 707.365 0.000 959.257 811.467

2 4 4 24953.930 157.968 2 0.000 0.000 0.000 0.000 0.000 26386.264 23521.596

3 4 4 503.627 22.442 3 425.758 0.000 0.000 505.215 0.000 579.907 0.000

4 4 4 1516.587 38.943 3 1278.670 0.000 0.000 1623.722 0.000 1647.367 0.000

0 5 4 458.161 21.405 3 0.000 0.000 0.000 438.935 0.000 467.644 467.905

1 5 4 501.187 22.387 2 0.000 0.000 0.000 442.322 0.000 560.053 0.000

2 5 4 1069.943 32.710 3 0.000 0.000 0.000 1000.163 0.000 1073.230 1136.437

3 5 4 541.203 23.264 3 468.507 0.000 0.000 558.686 0.000 596.415 0.000

4 5 4 1087.997 32.985 3 1262.122 0.000 0.000 986.480 0.000 1015.389 0.000

-2 6 4 2938.219 54.205 2 0.000 0.000 0.000 3051.001 0.000 0.000 2825.436

0 6 4 1895.104 43.533 3 0.000 0.000 0.000 1918.209 0.000 1889.792 1877.312

1 6 4 976.635 31.251 3 0.000 0.000 0.000 948.042 0.000 893.693 1088.169

2 6 4 205.644 14.340 1 0.000 0.000 0.000 205.644 0.000 0.000 0.000

2 7 4 640.114 25.300 2 0.000 0.000 0.000 519.694 0.000 760.534 0.000

3 7 4 1709.520 41.346 3 0.000 0.000 0.000 1436.429 0.000 1761.932 1930.198

0 -6 5 1114.561 33.385 5 984.202 877.754 1444.111 0.000 0.000 1219.290 1047.447

2 -6 5 60.190 7.758 1 0.000 0.000 0.000 0.000 0.000 60.190 0.000

-3 -5 5 273.470 16.537 1 273.470 0.000 0.000 0.000 0.000 0.000 0.000

-2 -5 5 4179.058 64.646 4 4024.934 3805.728 4263.055 0.000 0.000 0.000 4622.514

-1 -5 5 581.506 24.114 4 448.133 551.322 689.061 0.000 0.000 0.000 637.509

-3 -4 5 467.677 21.626 3 380.987 452.740 569.305 0.000 0.000 0.000 0.000

-2 -4 5 1509.266 38.849 4 1112.797 1359.075 1725.571 0.000 0.000 0.000 1839.619

-1 -4 5 1326.456 36.421 4 1337.404 996.370 1435.561 0.000 0.000 0.000 1536.491

1 -4 5 1346.745 36.698 4 1298.727 1339.011 1274.393 0.000 0.000 0.000 1474.849

2 -4 5 471.181 21.707 1 0.000 0.000 471.181 0.000 0.000 0.000 0.000

4 -4 5 1119.680 33.462 2 0.000 815.425 1423.935 0.000 0.000 0.000 0.000

-3 -3 5 415.352 20.380 3 419.218 347.938 478.901 0.000 0.000 0.000 0.000

-2 -3 5 6939.320 83.303 5 6150.017 6720.276 7745.137 6985.859 7095.313 0.000 0.000

-1 -3 5 3660.301 60.500 4 3646.127 3033.095 3688.802 0.000 0.000 0.000 4273.180

0 -3 5 8542.058 92.423 4 6761.824 7583.154 6586.331 0.000 0.000 0.000 13236.921

1 -3 5 5246.506 72.433 3 5519.620 4845.745 5374.154 0.000 0.000 0.000 0.000

2 -3 5 173.308 13.165 1 0.000 0.000 0.000 0.000 0.000 173.308 0.000

3 -3 5 665.383 25.795 3 731.859 572.121 692.167 0.000 0.000 0.000 0.000

5 -3 5 3818.425 61.793 2 0.000 3400.679 4236.171 0.000 0.000 0.000 0.000

-4 -2 5 531.782 23.060 3 446.746 584.593 0.000 0.000 564.008 0.000 0.000

-3 -2 5 506.885 22.514 5 520.948 386.853 531.565 447.533 647.528 0.000 0.000

-1 -2 5 3732.823 61.097 6 3936.116 4122.263 4988.975 4356.500 4710.522 0.000 282.566

0 -2 5 22213.283 149.041 3 22093.611 25557.826 18988.412 0.000 0.000 0.000 0.000

1 -2 5 2415.459 49.147 4 2420.928 2221.714 2032.053 0.000 0.000 2987.141 0.000

2 -2 5 1412.225 37.580 4 1571.276 1171.681 1246.336 0.000 0.000 1659.610 0.000

3 -2 5 6153.104 78.442 4 7747.136 5346.573 6205.098 0.000 0.000 5313.607 0.000

5 -2 5 252.725 15.897 1 0.000 0.000 252.725 0.000 0.000 0.000 0.000

-4 -1 5 2075.936 45.562 5 1632.149 1375.075 1753.972 1709.120 3909.362 0.000 0.000

-3 -1 5 10930.563 104.549 5 11928.837 10948.107 11080.154 9932.345 10763.372 0.000 0.000

-1 -1 5 1713.404 41.393 6 1266.977 1575.060 1799.341 1666.790 1686.216 0.000 2286.040

0 -1 5 533.400 23.095 5 612.124 517.742 520.597 523.120 493.415 0.000 0.000

1 -1 5 498.575 22.329 4 531.154 451.140 469.937 0.000 0.000 542.069 0.000

2 -1 5 3362.612 57.988 4 3747.689 2750.120 3601.970 0.000 0.000 3350.669 0.000

3 -1 5 3205.017 56.613 4 3690.858 3025.293 3124.950 0.000 0.000 2978.966 0.000

-4 0 5 2654.862 51.525 4 0.000 3053.169 3291.339 1905.787 2369.153 0.000 0.000

-3 0 5 11297.712 106.291 2 0.000 10826.420 11769.003 0.000 0.000 0.000 0.000

-2 0 5 6116.984 78.211 4 4072.271 0.000 5873.038 7061.659 7460.968 0.000 0.000

-1 0 5 1304.014 36.111 4 1099.954 0.000 0.000 1343.705 1116.333 0.000 1656.065

0 0 5 344.014 18.548 3 293.031 0.000 0.000 0.000 286.796 0.000 452.215

1 0 5 497.046 22.295 6 506.569 497.003 460.082 421.487 537.138 559.997 0.000

2 0 5 208.815 14.450 1 0.000 0.000 0.000 0.000 0.000 208.815 0.000

3 0 5 4166.389 64.548 4 4471.789 4698.309 3768.096 0.000 0.000 3727.361 0.000

4 0 5 4291.408 65.509 4 4853.827 2648.875 5633.396 0.000 0.000 4029.533 0.000

5 0 5 9979.824 99.899 2 9110.819 10848.829 0.000 0.000 0.000 0.000 0.000

6 0 5 1449.849 38.077 3 1482.626 1367.025 0.000 0.000 1499.895 0.000 0.000

-2 1 5 2398.064 48.970 2 0.000 0.000 0.000 2189.560 2606.568 0.000 0.000

1 1 5 1088.818 32.997 5 1087.686 0.000 0.000 800.721 1317.416 1126.796 1111.474

2 1 5 2449.894 49.496 4 2469.880 0.000 0.000 2118.050 3569.108 1642.538 0.000

3 1 5 241.307 15.534 2 298.868 0.000 0.000 0.000 0.000 183.747 0.000

4 1 5 2060.113 45.388 3 2024.809 0.000 2102.888 0.000 0.000 2052.642 0.000

5 1 5 8667.709 93.101 2 8097.285 9238.133 0.000 0.000 0.000 0.000 0.000

6 1 5 858.098 29.293 3 928.809 707.433 0.000 0.000 938.052 0.000 0.000

-3 2 5 931.967 30.528 2 0.000 0.000 0.000 939.269 924.664 0.000 0.000

-2 2 5 1072.900 32.755 2 0.000 0.000 0.000 1269.292 876.507 0.000 0.000

-1 2 5 1381.114 37.163 3 0.000 0.000 0.000 1426.327 1260.540 0.000 1456.476

0 2 5 704.680 26.546 4 593.375 0.000 0.000 666.035 788.713 0.000 770.596

1 2 5 2088.618 45.701 5 2462.954 0.000 0.000 1976.503 1769.726 1915.245 2318.664

2 2 5 5173.327 71.926 4 4923.936 0.000 0.000 4611.519 6585.883 4571.969 0.000

3 2 5 592.528 24.342 2 583.952 0.000 0.000 0.000 0.000 601.104 0.000

4 2 5 2089.992 45.716 3 2438.388 0.000 0.000 2077.980 0.000 1753.607 0.000

5 2 5 1766.926 42.035 3 2162.451 0.000 0.000 1744.978 0.000 1393.349 0.000

-2 3 5 7804.701 88.344 3 0.000 0.000 0.000 8174.645 7634.277 0.000 7605.182

0 3 5 8636.646 92.934 3 0.000 0.000 0.000 9533.372 7644.897 0.000 8731.665

2 3 5 609.658 24.691 4 576.559 0.000 0.000 532.737 0.000 549.191 780.145

3 3 5 1681.052 41.001 3 1319.903 0.000 0.000 1986.362 0.000 1736.892 0.000

5 3 5 4899.959 70.000 4 5216.253 0.000 0.000 3823.045 6835.000 3725.538 0.000

-2 4 5 7010.408 83.728 3 0.000 0.000 0.000 7118.847 6843.399 0.000 7068.977

0 4 5 1081.025 32.879 2 0.000 0.000 0.000 1083.465 0.000 0.000 1078.585

3 4 5 3408.048 58.378 3 3723.738 0.000 0.000 2935.490 0.000 3564.915 0.000

4 4 5 803.672 28.349 3 820.717 0.000 0.000 767.648 0.000 822.650 0.000

5 4 5 145.394 12.058 1 0.000 0.000 0.000 0.000 145.394 0.000 0.000

0 5 5 458.828 21.420 2 0.000 0.000 0.000 411.414 0.000 506.243 0.000

1 5 5 606.209 24.621 2 0.000 0.000 0.000 499.043 0.000 713.376 0.000

2 5 5 979.854 31.303 4 934.566 0.000 0.000 961.172 0.000 1033.438 990.241

4 5 5 418.072 20.447 2 341.409 0.000 0.000 494.735 0.000 0.000 0.000

-1 6 5 527.467 22.967 1 0.000 0.000 0.000 527.467 0.000 0.000 0.000

1 6 5 4654.578 68.224 3 0.000 0.000 0.000 4248.472 0.000 4957.784 4757.478

3 6 5 1879.253 43.350 4 1684.550 0.000 0.000 1487.232 0.000 2187.313 2157.919

0 -5 6 725.772 26.940 4 634.242 702.792 724.797 0.000 0.000 0.000 841.257

1 -5 6 1707.844 41.326 4 1219.234 1701.149 1731.763 0.000 0.000 0.000 2179.230

2 -5 6 2077.110 45.575 3 2013.077 2232.874 1985.379 0.000 0.000 0.000 0.000

-2 -4 6 319.321 17.870 2 274.253 0.000 0.000 0.000 0.000 0.000 364.390

0 -4 6 869.480 29.487 4 702.567 848.557 844.512 0.000 0.000 0.000 1082.283

-3 -3 6 555.033 23.559 3 517.083 470.042 0.000 0.000 677.975 0.000 0.000

-1 -3 6 1710.007 41.352 4 1653.494 1643.282 1807.708 1735.545 0.000 0.000 0.000

1 -3 6 2330.168 48.272 4 2206.161 2501.678 2222.229 0.000 0.000 2390.603 0.000

3 -3 6 265.692 16.300 2 0.000 0.000 239.097 0.000 0.000 292.288 0.000

4 -3 6 344.900 18.571 1 0.000 0.000 344.900 0.000 0.000 0.000 0.000

-3 -2 6 2528.310 50.282 5 2561.374 2223.344 3080.472 2076.894 2699.465 0.000 0.000

-1 -2 6 4288.663 65.488 5 4065.533 4603.395 4524.026 4413.250 3837.113 0.000 0.000

0 -2 6 2119.846 46.042 4 2364.761 2089.623 1964.586 2060.416 0.000 0.000 0.000

1 -2 6 273.177 16.528 1 0.000 0.000 0.000 0.000 0.000 273.177 0.000

3 -2 6 750.314 27.392 4 882.472 567.659 893.109 0.000 0.000 658.015 0.000

-2 -1 6 1357.088 36.839 5 1595.494 1311.613 1378.729 1199.344 1300.262 0.000 0.000

0 -1 6 558.450 23.632 5 613.700 519.372 587.770 580.753 490.657 0.000 0.000

1 -1 6 230.651 15.187 1 0.000 0.000 0.000 0.000 0.000 230.651 0.000

2 -1 6 5523.549 74.321 4 5364.716 3253.423 5694.426 0.000 0.000 7781.633 0.000

5 -1 6 995.969 31.559 3 1004.358 913.579 1069.970 0.000 0.000 0.000 0.000

-2 0 6 2131.561 46.169 5 1419.275 3000.598 3018.084 1630.273 1589.572 0.000 0.000

-1 0 6 6124.570 78.260 6 4985.265 6390.147 7100.868 6385.930 5213.672 0.000 6671.537

0 0 6 516.159 22.719 4 564.509 0.000 477.738 0.000 407.100 0.000 615.288

1 0 6 1902.944 43.623 7 1929.065 1862.209 2130.267 1665.820 1870.427 1957.987 1904.832

2 0 6 3999.626 63.243 6 4906.178 3272.274 3393.854 3954.199 4216.598 4254.650 0.000

4 0 6 3364.071 58.001 4 3585.680 3485.815 3620.445 0.000 0.000 2764.345 0.000

-2 1 6 296.617 17.223 2 0.000 0.000 0.000 243.947 0.000 0.000 349.287

0 1 6 3051.136 55.237 4 3083.243 0.000 0.000 3138.553 2690.859 0.000 3291.890

1 1 6 1430.251 37.819 5 1536.414 0.000 0.000 1302.772 1562.043 969.067 1780.961

2 1 6 3514.902 59.287 4 3964.527 0.000 0.000 2892.666 4119.493 3082.923 0.000

3 1 6 814.718 28.543 4 817.903 0.000 0.000 681.212 925.408 834.348 0.000

4 1 6 2205.084 46.958 4 1907.383 0.000 2051.156 2567.135 0.000 2294.662 0.000

5 1 6 237.073 15.397 1 0.000 0.000 237.073 0.000 0.000 0.000 0.000

-3 2 6 3045.615 55.187 1 0.000 0.000 0.000 3045.615 0.000 0.000 0.000

-2 2 6 1515.688 38.932 3 0.000 0.000 0.000 1738.990 1222.015 0.000 1586.060

0 2 6 4474.250 66.890 3 4702.034 0.000 0.000 4703.593 0.000 0.000 4017.121

1 2 6 169.101 13.004 1 0.000 0.000 0.000 0.000 169.101 0.000 0.000

3 2 6 1806.421 42.502 3 1855.140 0.000 0.000 1521.769 0.000 2042.353 0.000

-1 3 6 6887.094 82.989 3 0.000 0.000 0.000 8016.057 5654.675 0.000 6990.551

0 3 6 498.716 22.332 4 361.119 0.000 0.000 492.765 541.441 0.000 599.540

1 3 6 487.212 22.073 4 511.613 0.000 0.000 446.000 486.900 504.335 0.000

2 3 6 202.919 14.245 1 0.000 0.000 0.000 0.000 202.919 0.000 0.000

3 3 6 2986.667 54.650 4 3066.863 0.000 0.000 2819.340 3337.041 2723.426 0.000

4 3 6 2521.455 50.214 3 2588.229 0.000 0.000 2427.345 0.000 2548.793 0.000

5 3 6 1678.128 40.965 3 1831.705 0.000 0.000 1545.710 0.000 1656.970 0.000

-1 4 6 2421.181 49.205 3 0.000 0.000 0.000 2802.998 2007.734 0.000 2452.810

1 4 6 4106.989 64.086 5 3887.164 0.000 0.000 4084.246 4245.436 4214.407 4103.691

3 4 6 2293.771 47.893 4 2376.355 0.000 0.000 2325.129 0.000 2026.699 2446.901

0 5 6 612.364 24.746 2 0.000 0.000 0.000 631.546 0.000 0.000 593.181

2 5 6 1616.835 40.210 4 1502.772 0.000 0.000 1745.967 0.000 1800.775 1417.827

3 5 6 498.645 22.330 3 461.025 0.000 0.000 445.505 0.000 589.406 0.000

4 5 6 1359.459 36.871 3 1529.854 0.000 0.000 1158.256 0.000 1390.267 0.000

0 -4 7 5868.410 76.606 3 5442.277 6183.543 5979.408 0.000 0.000 0.000 0.000

1 -4 7 424.273 20.598 2 0.000 0.000 0.000 0.000 0.000 403.404 445.142

2 -4 7 3133.695 55.979 3 3800.665 3100.889 2499.531 0.000 0.000 0.000 0.000

1 -3 7 297.531 17.249 1 0.000 0.000 0.000 0.000 0.000 297.531 0.000

2 -3 7 759.193 27.553 3 848.305 708.128 721.145 0.000 0.000 0.000 0.000

-1 -2 7 795.021 28.196 5 790.949 738.090 857.838 744.251 843.979 0.000 0.000

1 -2 7 1500.457 38.736 4 1525.761 1435.764 1453.783 0.000 0.000 1586.520 0.000

2 -2 7 2170.677 46.591 4 2597.504 2064.023 2025.517 0.000 0.000 1995.665 0.000

3 -2 7 2519.455 50.194 4 2756.678 1913.199 2694.493 0.000 0.000 2713.447 0.000

4 -2 7 1259.074 35.483 4 1566.261 1214.909 1024.551 0.000 0.000 1230.574 0.000

2 -1 7 380.177 19.498 3 470.310 0.000 361.969 0.000 0.000 308.252 0.000

3 -1 7 1017.533 31.899 4 1096.288 1044.975 1131.426 0.000 0.000 797.441 0.000

4 -1 7 2147.521 46.341 4 2412.247 1974.226 2509.224 0.000 0.000 1694.385 0.000

-2 0 7 894.134 29.902 5 883.315 767.426 895.234 949.177 0.000 0.000 975.519

-1 0 7 927.457 30.454 6 922.189 802.496 952.127 959.512 825.431 0.000 1102.984

1 0 7 1289.092 35.904 7 1317.704 1301.437 1029.691 1184.652 1221.375 1345.279 1623.510

4 0 7 1433.440 37.861 4 1760.198 1466.730 1429.298 0.000 0.000 1077.533 0.000

-2 1 7 4045.684 63.606 4 3289.874 0.000 0.000 4922.445 3865.112 0.000 4105.304

0 1 7 3194.181 56.517 4 2863.423 0.000 0.000 3368.748 2712.184 0.000 3832.369

1 1 7 756.411 27.503 5 825.186 0.000 0.000 765.087 751.129 649.079 791.573

4 1 7 797.533 28.241 3 0.000 1659.113 306.209 0.000 427.276 0.000 0.000

-1 2 7 756.707 27.508 4 725.646 0.000 0.000 833.114 730.247 0.000 737.822

2 2 7 803.094 28.339 5 966.673 0.000 0.000 760.778 826.966 673.584 787.472

-1 3 7 2673.906 51.710 4 2782.790 0.000 0.000 3015.929 2331.248 0.000 2565.657

0 3 7 2659.041 51.566 4 2440.846 0.000 0.000 2922.312 2375.527 0.000 2897.480

1 3 7 523.093 22.871 4 582.287 0.000 0.000 463.157 534.737 512.190 0.000

2 3 7 3841.515 61.980 5 4298.087 0.000 0.000 4141.327 3313.287 3756.892 3697.981

3 3 7 2032.064 45.078 4 2285.536 0.000 0.000 1685.364 2043.388 2113.969 0.000

4 3 7 2864.563 53.522 3 3074.889 0.000 0.000 2933.589 0.000 2585.211 0.000

1 4 7 5450.259 73.826 5 5038.855 0.000 0.000 5389.260 5151.572 7162.264 4509.345

2 4 7 2212.288 47.035 4 0.000 0.000 0.000 1804.300 2061.380 2268.776 2714.698

3 4 7 3075.923 55.461 5 3027.106 0.000 0.000 2674.222 3316.554 3272.344 3089.386

1 -2 8 508.351 22.547 5 537.447 0.000 495.677 0.000 296.042 724.594 487.995

0 -1 8 1574.664 39.682 5 1506.369 1847.472 1493.375 1682.278 1343.824 0.000 0.000

1 -1 8 4181.569 64.665 7 4484.196 4676.625 2958.156 4961.087 2753.158 4412.379 5025.379

2 -1 8 2208.648 46.996 6 2922.899 2279.959 1909.768 2495.459 1380.646 2263.157 0.000

3 -1 8 2418.671 49.180 4 3235.789 2459.135 1862.831 0.000 0.000 2116.928 0.000

0 0 8 2641.717 51.398 5 2547.947 2867.831 2693.127 2970.445 2129.233 0.000 0.000

1 0 8 523.234 22.874 4 480.349 0.000 417.152 0.000 0.000 652.030 543.405

2 0 8 182.206 13.498 1 0.000 0.000 0.000 0.000 182.206 0.000 0.000

3 0 8 2532.895 50.328 6 3011.707 2337.975 2772.017 2237.228 2899.550 1938.895 0.000

0 1 8 952.574 30.864 3 1023.612 0.000 0.000 1082.097 752.014 0.000 0.000

2 1 8 2230.155 47.225 5 2746.165 0.000 0.000 2034.622 2436.177 1631.263 2302.548

3 1 8 1594.029 39.925 4 1214.666 0.000 3410.448 0.000 914.073 836.932 0.000

1 2 8 563.229 23.732 4 591.126 0.000 0.000 0.000 502.849 608.912 550.029

3 2 8 676.935 26.018 3 837.345 0.000 0.000 510.892 0.000 682.566 0.000

2 3 8 6155.149 78.455 4 6879.578 0.000 0.000 5714.374 5091.392 0.000 6935.256

+++++++++++++++++++++++++++++++++++++++++++++++++++++++++++++++++++++++

+ SHELXL-97 - CRYSTAL STRUCTURE REFINEMENT - W95/98/NT/2000 VERSION +

+ Copyright(C) George M. Sheldrick 1993-2001 Release 97-2 +

+ axi_sca started at 19:15:33 on 24-Jan-2015 +

+++++++++++++++++++++++++++++++++++++++++++++++++++++++++++++++++++++++

TITL AXINITE (SUERT) P-1 ALBA:12_DES_14 UNIQUE_FILE with merged AX2

CELL 0.4246 7.1233 8.8906 9.2070 87.7840 77.218 81.6510

ZERR 2 0.0005 0.0003 0.0006 0.001 0.001 0.001

LATT 1

SFAC FE CA SI AL O B

UNIT 2 4 8 4 32 2

V = 562.59 F(000) = 562.0 Mu = 1.51 mm-1 Cell Wt = 1138.28 Rho = 3.360

TEMP 20

L.S. 10

OMIT -3 0 5

OMIT 0 -1 3

BOND 0.5

FMAP 2

LIST 4

ACTA

WGHT 0.100000

FVAR 0.16559 1.000 1.000 1.000

Ca1 2 0.1855 0.9161 0.5981 21.0000 0.05

Ca2 2 0.2519 0.3924 0.1558 31.0000 0.05

X3 1 0.7663 0.8879 0.0938 41.0000 0.05

Al4 4 0.0596 0.7465 0.2977 11.0000 0.05

Al5 4 0.3621 0.5801 0.4315 11.0000 0.05

Si6 3 0.6926 0.9932 0.7565 11.0000 0.05

Si7 3 0.2124 0.7693 0.9563 11.0000 0.05

Si8 3 0.3597 0.2238 0.4817 11.0000 0.05

Si9 3 0.7899 0.5214 0.2172 11.0000 0.05

B10 6 0.4532 0.7142 0.1329 11.0000 0.05

O11 5 0.1289 0.9328 0.3422 11.0000 0.05

O12 5 0.3155 0.6493 0.2491 11.0000 0.05

O13 5 0.9881 0.5562 0.2498 11.0000 0.05

O14 5 0.7972 0.8251 0.3721 11.0000 0.05

O15 5 0.7029 0.6249 0.5987 11.0000 0.05

O16 5 0.0650 0.8019 0.0991 11.0000 0.05

O17 5 0.4469 0.8792 0.1620 11.0000 0.05

O18 5 0.4249 0.6828 0.9911 11.0000 0.05

O19 5 0.3856 0.0923 0.3678 11.0000 0.05

O20 5 0.6222 0.4967 0.3693 11.0000 0.05

O21 5 0.2478 0.1361 0.1295 11.0000 0.05

O22 5 0.5652 0.2423 0.5133 11.0000 0.05

O23 5 0.8634 0.3583 0.1270 11.0000 0.05

O24 5 0.7751 0.0958 0.1727 11.0000 0.05

O25 5 0.3252 0.3640 0.8850 11.0000 0.05

OH26 5 0.1106 0.6802 0.4970 11.0000 0.05

HKLF 4

Covalent radii and connectivity table for AXINITE (SUERT) P-1 ALBA:12_DES_14 UNIQUE_FILE with merged AX2

FE 1.240

CA 1.970

SI 1.170

AL 1.250

O 0.660

B 0.820

Ca1 - O22_$3 O24_$3 O14_$5 O11_$4 O11 OH26 O19_$10 O19_$3 Si8_$10 Si8_$3 Si6_$5 Al4_$4

Ca2 - O13_$8 O21 O15_$3 O18_$3 O25_$9 O12 O23_$8 B10_$7 Si9_$8 Al5 Al4 Si6_$3

X3 - O24_$10 O21_$7 O16_$2 O17 Ca2_$7 Ca1_$5

Al4 - O12 O16 O11 O14_$8 O13_$8 OH26 Al5 Ca1_$4 Ca2 Ca1

Al5 - O12 O22_$3 OH26 O20 O20_$3 O15_$3 Al5_$3 Al4 Ca1 Ca2

Si6 - O17_$5 O11_$5 O21_$3 O19_$3 Ca1_$5 Ca1_$2 Ca2_$3

Si7 - O16_$6 O23_$3 O24_$3 O18 Ca1

Si8 - O19 O15_$3 O22 O14_$3 Ca1_$1 Ca1_$3 Ca2

Si9 - O13 O25_$3 O23 O20 Ca2_$2 Ca2_$7

B10 - O18_$9 O12 O17 O25_$3 Ca2_$7

O11 - Si6_$5 Al4 Ca1_$4 Ca1

O12 - B10 Al5 Al4 Ca2

O13 - Si9 Al4_$2 Ca2_$2

O14 - Si8_$3 Al4_$2 Ca1_$5

O15 - Si8_$3 Al5_$3 Ca2_$3

O16 - Si7_$9 Al4 X3_$8

O17 - B10 Si6_$5 X3

O18 - B10_$6 Si7 Ca2_$3

O19 - Si8 Si6_$3 Ca1_$1 Ca1_$3

O20 - Si9 Al5 Al5_$3

O21 - Si6_$3 X3_$7 Ca2

O22 - Si8 Al5_$3 Ca1_$3

O23 - Si7_$3 Si9 Ca2_$2

O24 - Si7_$3 X3_$1 Ca1_$3

O25 - B10_$3 Si9_$3 Ca2_$6

OH26 - Al5 Al4 Ca1

Operators for generating equivalent atoms:

$1 x, y-1, z

$2 x+1, y, z

$3 -x+1, -y+1, -z+1

$4 -x, -y+2, -z+1

$5 -x+1, -y+2, -z+1

$6 x, y, z+1

$7 -x+1, -y+1, -z

$8 x-1, y, z

$9 x, y, z-1

$10 x, y+1, z

616 Reflections read, of which 2 rejected

-6 =< h =< 6, -8 =< k =< 8, 0 =< l =< 8, Max. 2-theta = 22.78

0 Systematic absence violations

0 Inconsistent equivalents

614 Unique reflections, of which 0 suppressed

R(int) = 0.0000 R(sigma) = 0.0184 Friedel opposites merged

Maximum memory for data reduction = 3584 / 6832

Least-squares cycle 1 Maximum vector length = 511 Memory required = 3842 / 141517

wR2 = 0.5024 before cycle 1 for 614 data and 108 / 108 parameters

GooF = S = 1.414; Restrained GooF = 1.414 for 0 restraints

Weight = 1 / [ sigma^2(Fo^2) + ( 0.1000 * P )^2 + 0.00 * P ] where P = ( Max ( Fo^2, 0 ) + 2 * Fc^2 ) / 3

N value esd shift/esd parameter

1 0.39710 0.01652 14.018 OSF

2 0.99535 0.06311 -0.074 FVAR 2

3 0.99291 0.06442 -0.110 FVAR 3

4 0.87160 0.05170 -2.484 FVAR 4

12 0.02334 0.00867 -3.075 U11 Ca2

20 0.01448 0.00774 -4.587 U11 Al4

21 0.34950 0.00385 -3.270 x Al5

24 0.02054 0.00809 -3.642 U11 Al5

28 0.02262 0.00777 -3.522 U11 Si6

32 0.01927 0.00737 -4.172 U11 Si7

36 0.02200 0.00768 -3.648 U11 Si8

39 0.22604 0.00268 3.294 z Si9

40 0.02156 0.00787 -3.615 U11 Si9

Mean shift/esd = 1.409 Maximum = 14.018 for OSF

Max. shift = 0.158 A for O15 Max. dU =-0.039 for O11

Least-squares cycle 2 Maximum vector length = 511 Memory required = 3842 / 141517

wR2 = 0.1962 before cycle 2 for 614 data and 108 / 108 parameters

GooF = S = 1.913; Restrained GooF = 1.913 for 0 restraints

Weight = 1 / [ sigma^2(Fo^2) + ( 0.1000 * P )^2 + 0.00 * P ] where P = ( Max ( Fo^2, 0 ) + 2 * Fc^2 ) / 3

N value esd shift/esd parameter

1 0.38186 0.00485 -3.143 OSF

2 0.99619 0.01890 0.045 FVAR 2

3 0.98237 0.01898 -0.556 FVAR 3

4 0.80858 0.01485 -4.243 FVAR 4

5 0.18230 0.00064 -3.028 x Ca1

16 0.02100 0.00207 -5.411 U11 X3

36 0.01506 0.00189 -3.673 U11 Si8

40 0.01518 0.00190 -3.350 U11 Si9

Mean shift/esd = 0.906 Maximum = -5.411 for U11 X3

Max. shift = 0.039 A for O21 Max. dU =-0.011 for X3

------------------------------------------------------

Least-squares cycle 9 Maximum vector length = 511 Memory required = 3842 / 141517

wR2 = 0.1542 before cycle 9 for 614 data and 108 / 108 parameters

GooF = S = 1.488; Restrained GooF = 1.488 for 0 restraints

Weight = 1 / [ sigma^2(Fo^2) + ( 0.1000 * P )^2 + 0.00 * P ] where P = ( Max ( Fo^2, 0 ) + 2 * Fc^2 ) / 3

N value esd shift/esd parameter

1 0.38212 0.00367 0.000 OSF

2 0.99345 0.01476 0.000 FVAR 2

3 0.98192 0.01492 0.000 FVAR 3

4 0.80624 0.01116 0.000 FVAR 4

Mean shift/esd = 0.000 Maximum = 0.000 for z Ca1

Max. shift = 0.000 A for O14 Max. dU = 0.000 for OH26

Least-squares cycle 10 Maximum vector length = 511 Memory required = 3842 / 141517

wR2 = 0.1542 before cycle 10 for 614 data and 108 / 108 parameters

GooF = S = 1.488; Restrained GooF = 1.488 for 0 restraints

Weight = 1 / [ sigma^2(Fo^2) + ( 0.1000 * P )^2 + 0.00 * P ] where P = ( Max ( Fo^2, 0 ) + 2 * Fc^2 ) / 3

N value esd shift/esd parameter

1 0.38212 0.00367 0.000 OSF

2 0.99345 0.01476 0.000 FVAR 2

3 0.98192 0.01492 0.000 FVAR 3

4 0.80624 0.01116 0.000 FVAR 4

Mean shift/esd = 0.000 Maximum = 0.000 for y Ca1

Max. shift = 0.000 A for Si7 Max. dU = 0.000 for O18

Largest correlation matrix elements

0.738 U11 Ca1 / FVAR 2 0.588 U11 Al4 / OSF 0.566 U11 Si7 / OSF

0.737 U11 Ca2 / FVAR 3 0.584 U11 Si9 / OSF 0.564 U11 Si6 / OSF

0.736 U11 X3 / FVAR 4 0.576 U11 Si8 / OSF 0.537 U11 Al5 / OSF

AXINITE (SUERT) P-1 ALBA:12_DES_14 UNIQUE_FILE with merged AX2

ATOM x y z sof U11 U22 U33 U23 U13 U12 Ueq

Ca1 0.18223 0.91611 0.60131 0.99345 0.01903

0.00505 0.00049 0.00040 0.00037 0.01476 0.00176

Ca2 0.25435 0.39391 0.15173 0.98192 0.01841

0.00500 0.00049 0.00041 0.00038 0.01492 0.00173

X3 0.76642 0.88755 0.09221 0.80624 0.02046

0.00434 0.00044 0.00036 0.00033 0.01116 0.00162

Al4 0.05231 0.74684 0.30036 1.00000 0.01223

0.00704 0.00069 0.00055 0.00054 0.00000 0.00145

Al5 0.35142 0.57972 0.43592 1.00000 0.01547

0.00702 0.00066 0.00058 0.00053 0.00000 0.00148

Si6 0.69826 0.98888 0.75679 1.00000 0.01810

0.00696 0.00068 0.00054 0.00051 0.00000 0.00144

Si7 0.21046 0.76735 0.95004 1.00000 0.01544

0.00700 0.00068 0.00055 0.00051 0.00000 0.00132

Si8 0.35867 0.23055 0.48087 1.00000 0.01522

0.00692 0.00065 0.00054 0.00051 0.00000 0.00142

Si9 0.78192 0.52232 0.22595 1.00000 0.01542

0.00662 0.00067 0.00053 0.00049 0.00000 0.00145

B10 0.46080 0.71183 0.13416 1.00000 0.01299

0.02727 0.00266 0.00228 0.00206 0.00000 0.00477

O11 0.12509 0.93271 0.34522 1.00000 0.01180

0.01445 0.00144 0.00115 0.00109 0.00000 0.00261

O12 0.32278 0.64670 0.25031 1.00000 0.01964

0.01503 0.00151 0.00123 0.00110 0.00000 0.00287

O13 0.97684 0.56241 0.25678 1.00000 0.01411

0.01486 0.00148 0.00119 0.00110 0.00000 0.00268

O14 0.79120 0.82354 0.37648 1.00000 0.01847

0.01529 0.00154 0.00123 0.00115 0.00000 0.00281

O15 0.72050 0.61495 0.59949 1.00000 0.01424

0.01455 0.00150 0.00117 0.00112 0.00000 0.00281

O16 0.05472 0.80954 0.10272 1.00000 0.01666

0.01468 0.00149 0.00119 0.00111 0.00000 0.00279

O17 0.46359 0.87543 0.15687 1.00000 0.01861

0.01574 0.00161 0.00127 0.00115 0.00000 0.00280

O18 0.41786 0.68722 0.98812 1.00000 0.01856

0.01466 0.00152 0.00121 0.00117 0.00000 0.00294

O19 0.39509 0.08711 0.36489 1.00000 0.02154

0.01559 0.00156 0.00127 0.00114 0.00000 0.00287

O20 0.61829 0.49610 0.37117 1.00000 0.01913

0.01509 0.00158 0.00124 0.00115 0.00000 0.00289

O21 0.23026 0.13791 0.13332 1.00000 0.02410

0.01597 0.00168 0.00131 0.00118 0.00000 0.00297

O22 0.56442 0.24439 0.51634 1.00000 0.01510

0.01492 0.00148 0.00117 0.00113 0.00000 0.00272

O23 0.86186 0.36848 0.12998 1.00000 0.01918

0.01534 0.00150 0.00120 0.00107 0.00000 0.00282

O24 0.77210 0.09395 0.15994 1.00000 0.02001

0.01463 0.00163 0.00120 0.00115 0.00000 0.00290

O25 0.32621 0.35416 0.87869 1.00000 0.01572

0.01496 0.00146 0.00124 0.00110 0.00000 0.00274

OH26 0.09548 0.67677 0.49609 1.00000 0.01912

0.01612 0.00163 0.00135 0.00119 0.00000 0.00280

Final Structure Factor Calculation for AXINITE (SUERT) P-1 ALBA:12_DES_14 UNIQUE_FILE with merged AX2

Total number of l.s. parameters = 108 Maximum vector length = 511 Memory required = 3734 / 25039

wR2 = 0.1542 before cycle 11 for 614 data and 0 / 108 parameters

GooF = S = 1.488; Restrained GooF = 1.488 for 0 restraints

Weight = 1 / [ sigma^2(Fo^2) + ( 0.1000 * P )^2 + 0.00 * P ] where P = ( Max ( Fo^2, 0 ) + 2 * Fc^2 ) / 3

R1 = 0.0643 for 602 Fo > 4sig(Fo) and 0.0648 for all 614 data

wR2 = 0.1542, GooF = S = 1.488, Restrained GooF = 1.488 for all data

Occupancy sum of asymmetric unit = 25.78 for non-hydrogen and 0.00 for hydrogen atoms

Analysis of variance for reflections employed in refinement K = Mean[Fo^2] / Mean[Fc^2] for group

Fc/Fc(max) 0.000 0.090 0.114 0.136 0.163 0.192 0.230 0.276 0.337 0.435 1.000

Number in group 63. 63. 62. 61. 58. 62. 61. 61. 62. 61.

GooF 1.106 1.353 1.306 1.418 1.242 1.448 1.478 1.499 1.623 2.165

K 0.896 0.906 0.920 0.927 0.983 1.018 1.005 1.039 1.083 1.145

Resolution(A) 1.07 1.12 1.16 1.24 1.30 1.39 1.49 1.64 1.89 2.31 inf

Number in group 63. 60. 62. 61. 61. 62. 62. 63. 59. 61.

GooF 1.473 1.449 0.982 1.168 1.326 1.292 1.660 1.497 2.038 1.747

K 1.053 1.015 1.032 1.051 1.049 1.075 1.158 1.111 1.135 1.024

R1 0.057 0.057 0.044 0.050 0.057 0.056 0.072 0.075 0.093 0.080

Recommended weighting scheme: WGHT 0.1449 0.0000

Note that in most cases convergence will be faster if fixed weights (e.g. the

default WGHT 0.1) are retained until the refinement is virtually complete, and

only then should the above recommended values be used.

Most Disagreeable Reflections (* if suppressed or used for Rfree)

h k l Fo^2 Fc^2 Delta(F^2)/esd Fc/Fc(max) Resolution(A)

4 3 2 6355.42 3841.73 3.97 0.402 1.60

-2 -2 2 849.22 1568.66 3.77 0.257 2.26

-4 0 5 1814.85 1117.07 3.72 0.217 1.13

2 -3 2 1164.25 1988.99 3.34 0.289 2.00

0 4 4 27757.01 18342.29 3.24 0.879 1.58

1 1 3 10471.37 16837.99 3.20 0.842 2.89

2 -2 2 1239.58 1986.58 3.10 0.289 2.38

4 1 7 541.03 294.31 2.86 0.111 1.16

0 4 1 7067.66 4974.31 2.72 0.458 2.14

-1 -5 4 17258.24 12216.33 2.69 0.717 1.35

-2 7 1 1362.85 967.08 2.65 0.202 1.11

1 -3 4 527.34 751.70 2.43 0.178 1.76

-5 0 3 205.46 319.43 2.41 0.116 1.16

-1 0 1 239.70 386.29 2.39 0.128 4.96

-1 3 1 12950.53 9530.66 2.37 0.633 2.42

4 -1 3 1890.19 1356.49 2.37 0.239 1.59

-5 3 2 5650.02 4166.18 2.36 0.419 1.10

2 3 4 16217.27 11985.55 2.34 0.710 1.79

1 4 2 6499.23 4796.30 2.33 0.449 2.03

-3 1 1 7019.72 5197.46 2.32 0.468 2.00

4 0 4 1082.06 796.58 2.31 0.183 1.54

-1 6 1 3129.77 2327.71 2.27 0.313 1.37

3 2 6 1232.73 1709.38 2.19 0.268 1.37

3 3 0 6718.39 5064.14 2.18 0.462 1.94

2 2 0 410.91 604.84 2.16 0.160 2.91

-1 -2 5 2554.49 3470.98 2.14 0.382 1.56

3 2 1 828.67 1160.08 2.14 0.221 2.21

2 6 2 10608.34 8052.88 2.12 0.582 1.40

0 -8 1 1725.82 1290.42 2.10 0.233 1.09

1 -1 1 308.18 436.09 2.04 0.135 4.76

-2 3 5 5341.84 4111.39 2.01 0.416 1.28

-1 -5 3 8362.03 6437.51 2.01 0.521 1.48

-5 -3 1 691.70 940.17 2.00 0.199 1.27

1 -1 3 9478.34 7305.61 2.00 0.555 2.78

6 -2 3 109.58 173.46 2.00 0.085 1.08

3 1 8 1088.91 831.71 1.99 0.187 1.11

3 1 2 178.06 348.61 1.97 0.121 2.26

1 -4 1 4780.26 3697.38 1.95 0.395 1.99

-2 -3 2 376.67 488.21 1.82 0.143 1.99

3 -5 3 2876.37 2273.31 1.80 0.309 1.26

-4 -2 5 362.97 470.71 1.77 0.141 1.11

4 2 1 3568.07 2839.86 1.75 0.346 1.72

4 5 4 739.64 987.60 1.74 0.204 1.24

3 -6 2 1198.49 947.59 1.73 0.200 1.16

-1 4 0 2561.34 2044.85 1.72 0.293 2.01

-1 1 4 143.82 303.34 1.71 0.113 1.94

-1 5 1 16676.12 13342.39 1.71 0.749 1.61

0 4 0 2047.70 1639.52 1.70 0.263 2.20

2 -1 6 3780.38 3027.05 1.70 0.357 1.46

4 -2 3 7567.60 6061.10 1.69 0.505 1.48

Bond lengths and angles

Ca1 - Distance Angles

O22_$3 2.2301 (0.0120)

O24_$3 2.2888 (0.0106) 102.16 (0.42)

O14_$5 2.3698 (0.0112) 120.57 (0.41) 82.44 (0.37)

O11_$4 2.3582 (0.0114) 160.54 (0.38) 96.12 (0.41) 68.21 (0.37)

O11 2.4752 (0.0101) 79.44 (0.35) 178.40 (0.42) 96.63 (0.35) 82.32 (0.39)

OH26 2.5796 (0.0119) 64.85 (0.38) 118.09 (0.38) 158.26 (0.38) 100.58 (0.37) 62.66 (0.33)

O19_$10 2.8812 (0.0101) 73.42 (0.36) 123.69 (0.34) 57.12 (0.35) 101.89 (0.33) 56.50 (0.29) 110.26 (0.34)

O19_$3 3.0906 (0.0109) 55.61 (0.34) 63.93 (0.35) 76.13 (0.34) 141.29 (0.34) 117.15 (0.33) 117.99 (0.36) 69.09 (0.33)

Si8_$10 3.2846 (0.0053) 96.64 (0.28) 100.66 (0.27) 27.34 (0.29) 86.40 (0.26) 78.94 (0.24) 139.28 (0.27) 30.13 (0.25)

Si8_$3 3.2727 (0.0060) 25.75 (0.26) 83.55 (0.32) 100.79 (0.30) 168.89 (0.27) 97.92 (0.27) 89.27 (0.31) 69.55 (0.25)

Si6_$5 3.3256 (0.0053) 69.70 (0.27) 152.59 (0.28) 80.04 (0.28) 96.73 (0.26) 27.87 (0.23) 83.04 (0.26) 29.44 (0.23)

Al4_$4 3.2675 (0.0066) 154.08 (0.30) 83.51 (0.31) 34.36 (0.28) 34.69 (0.24) 94.99 (0.29) 134.67 (0.31) 82.36 (0.27)

Ca1 - O22_$3 O24_$3 O14_$5 O11_$4 O11 OH26 O19_$10

Ca2 - Distance Angles

O13_$8 2.3438 (0.0118)

O21 2.3225 (0.0115) 119.44 (0.41)

O15_$3 2.3342 (0.0102) 79.77 (0.37) 96.27 (0.36)

O18_$3 2.4254 (0.0115) 157.86 (0.40) 81.99 (0.39) 104.81 (0.38)

O25_$9 2.4806 (0.0107) 117.78 (0.38) 77.14 (0.34) 162.35 (0.41) 58.35 (0.34)

O12 2.6046 (0.0111) 64.94 (0.34) 160.92 (0.37) 65.50 (0.33) 96.80 (0.34) 118.51 (0.35)

O23_$8 2.8884 (0.0106) 54.61 (0.32) 71.85 (0.37) 110.49 (0.34) 137.70 (0.32) 83.30 (0.31) 118.50 (0.34)

B10_$7 3.0337 (0.0201) 145.45 (0.42) 74.76 (0.45) 132.57 (0.45) 28.66 (0.39) 30.04 (0.41) 112.75 (0.43) 110.37 (0.40)

Si9_$8 3.3200 (0.0061) 25.43 (0.25) 97.60 (0.32) 94.73 (0.29) 160.40 (0.27) 102.32 (0.27) 89.83 (0.27) 29.18 (0.23)

Al5 3.3941 (0.0055) 71.44 (0.26) 128.97 (0.28) 33.39 (0.26) 100.21 (0.25) 146.30 (0.24) 32.29 (0.24) 122.09 (0.25)

Al4 3.4553 (0.0064) 30.95 (0.25) 148.85 (0.34) 73.24 (0.29) 128.78 (0.26) 120.47 (0.29) 34.32 (0.25) 84.28 (0.24)

Si6_$3 3.4606 (0.0059) 121.81 (0.28) 22.57 (0.27) 75.18 (0.28) 80.02 (0.27) 95.25 (0.28) 138.38 (0.26) 87.07 (0.23)

Ca2 - O13_$8 O21 O15_$3 O18_$3 O25_$9 O12 O23_$8

X3 - Distance Angles

O24_$10 1.9695 (0.0108)

O21_$7 2.0921 (0.0108) 117.03 (0.44)

O16_$2 2.0922 (0.0109) 94.34 (0.43) 102.58 (0.45)

O17 2.1245 (0.0106) 100.21 (0.45) 92.51 (0.44) 151.46 (0.42)

Ca2_$7 3.4666 (0.0047) 157.61 (0.33) 40.68 (0.30) 93.87 (0.31) 81.87 (0.32)

Ca1_$5 3.4880 (0.0045) 38.14 (0.30) 154.21 (0.30) 77.99 (0.30) 98.69 (0.31) 164.10 (0.13)

X3 - O24_$10 O21_$7 O16_$2 O17 Ca2_$7

Al4 - Distance Angles

O12 1.9641 (0.0118)

O16 1.8795 (0.0109) 93.04 (0.50)

O11 1.8885 (0.0107) 92.91 (0.48) 91.45 (0.46)

O14_$8 1.8728 (0.0117) 170.72 (0.52) 95.82 (0.51) 89.63 (0.51)

O13_$8 1.8818 (0.0113) 87.73 (0.49) 88.69 (0.46) 179.34 (0.58) 89.72 (0.50)

OH26 1.9578 (0.0116) 78.17 (0.51) 170.77 (0.59) 86.26 (0.48) 93.10 (0.51) 93.70 (0.50)

Al5 2.9015 (0.0063) 38.50 (0.32) 131.53 (0.41) 90.44 (0.36) 132.62 (0.39) 89.95 (0.39) 39.68 (0.39)

Ca1_$4 3.2675 (0.0066) 138.12 (0.36) 86.72 (0.39) 45.29 (0.35) 45.57 (0.33) 134.09 (0.40) 97.92 (0.43) 125.73 (0.19)

Ca2 3.4553 (0.0064) 48.39 (0.32) 85.75 (0.39) 140.82 (0.40) 129.55 (0.37) 39.83 (0.35) 90.37 (0.42) 63.85 (0.14)

Ca1 3.5581 (0.0056) 86.46 (0.33) 132.49 (0.35) 41.28 (0.31) 89.58 (0.36) 138.64 (0.36) 45.07 (0.35) 61.45 (0.15)

Al4 - O12 O16 O11 O14_$8 O13_$8 OH26 Al5

Al5 - Distance Angles

O12 1.8321 (0.0112)

O22_$3 1.8547 (0.0112) 94.67 (0.49)

OH26 1.8731 (0.0132) 83.71 (0.51) 88.26 (0.52)

O20 1.9090 (0.0122) 94.62 (0.50) 87.75 (0.49) 175.54 (0.51)

O20_$3 1.9210 (0.0110) 178.44 (0.57) 86.80 (0.46) 96.87 (0.53) 84.90 (0.52)

O15_$3 1.9337 (0.0109) 90.76 (0.48) 173.88 (0.50) 95.15 (0.52) 89.00 (0.48) 87.75 (0.49)

Al5_$3 2.8261 (0.0094) 137.22 (0.46) 86.30 (0.37) 139.01 (0.47) 42.61 (0.32) 42.28 (0.37) 87.80 (0.40)

Al4 2.9015 (0.0063) 41.87 (0.37) 92.98 (0.38) 41.87 (0.35) 136.43 (0.38) 138.66 (0.42) 92.95 (0.35) 178.80 (0.28)

Ca1 3.3480 (0.0067) 95.09 (0.40) 38.82 (0.36) 49.99 (0.35) 126.26 (0.36) 86.39 (0.40) 143.44 (0.40) 110.90 (0.24)

Ca2 3.3941 (0.0055) 49.41 (0.35) 143.33 (0.37) 93.75 (0.38) 88.26 (0.35) 129.06 (0.38) 41.63 (0.31) 114.07 (0.27)

Al5 - O12 O22_$3 OH26 O20 O20_$3 O15_$3 Al5_$3

Si6 - Distance Angles

O17_$5 1.6375 (0.0132)

O11_$5 1.6225 (0.0104) 107.84 (0.59)

O21_$3 1.5896 (0.0120) 113.09 (0.63) 112.95 (0.60)

O19_$3 1.6348 (0.0118) 108.44 (0.57) 103.64 (0.54) 110.37 (0.60)

Ca1_$5 3.3256 (0.0053) 108.33 (0.40) 45.49 (0.35) 138.11 (0.54) 60.04 (0.38)

Ca1_$2 3.4048 (0.0059) 140.41 (0.42) 38.35 (0.39) 78.58 (0.46) 101.46 (0.45) 65.46 (0.13)

Ca2_$3 3.4606 (0.0059) 127.09 (0.40) 122.49 (0.44) 34.12 (0.42) 76.25 (0.40) 117.81 (0.17) 84.51 (0.14)

Si6 - O17_$5 O11_$5 O21_$3 O19_$3 Ca1_$5 Ca1_$2

Si7 - Distance Angles

O16_$6 1.6054 (0.0118)

O23_$3 1.6383 (0.0118) 107.86 (0.54)

O24_$3 1.5665 (0.0111) 112.22 (0.63) 108.49 (0.56)

O18 1.6485 (0.0120) 109.38 (0.58) 103.74 (0.57) 114.60 (0.58)

Ca1 3.4611 (0.0055) 126.02 (0.44) 76.55 (0.36) 32.01 (0.39) 121.95 (0.41)

Si7 - O16_$6 O23_$3 O24_$3 O18

Si8 - Distance Angles

O19 1.6494 (0.0125)

O15_$3 1.6227 (0.0112) 109.23 (0.58)

O22 1.5928 (0.0109) 106.38 (0.65) 110.00 (0.54)

O14_$3 1.6050 (0.0115) 103.19 (0.57) 115.21 (0.61) 112.23 (0.54)

Ca1_$1 3.2846 (0.0053) 61.27 (0.37) 134.97 (0.41) 114.90 (0.39) 42.70 (0.42)

Ca1_$3 3.2727 (0.0060) 68.94 (0.42) 121.32 (0.43) 37.47 (0.43) 122.31 (0.43) 97.24 (0.15)

Ca2 3.4910 (0.0053) 75.17 (0.39) 34.19 (0.39) 118.31 (0.38) 127.73 (0.41) 118.09 (0.14) 106.05 (0.15)

Si8 - O19 O15_$3 O22 O14_$3 Ca1_$1 Ca1_$3

Si9 - Distance Angles

O13 1.5689 (0.0111)

O25_$3 1.6517 (0.0115) 115.50 (0.54)

O23 1.6187 (0.0120) 100.36 (0.62) 107.48 (0.59)

O20 1.6019 (0.0119) 115.34 (0.57) 105.79 (0.63) 112.22 (0.59)

Ca2_$2 3.3200 (0.0061) 39.91 (0.44) 124.56 (0.42) 60.45 (0.39) 129.35 (0.45)

Ca2_$7 3.5853 (0.0056) 111.57 (0.40) 37.24 (0.42) 72.03 (0.36) 130.75 (0.43) 96.53 (0.13)

Si9 - O13 O25_$3 O23 O20 Ca2_$2

B10 - Distance Angles

O18_$9 1.4743 (0.0207)

O12 1.4483 (0.0208) 109.49 (1.17)

O17 1.4810 (0.0230) 110.60 (1.32) 112.85 (1.52)

O25_$3 1.5257 (0.0220) 105.75 (1.38) 115.73 (1.28) 102.03 (1.11)

Ca2_$7 3.0337 (0.0200) 52.10 (0.75) 137.30 (1.19) 109.84 (0.96) 54.48 (0.73)

B10 - O18_$9 O12 O17 O25_$3

O11 - Distance Angles

Si6_$5 1.6225 (0.0103)

Al4 1.8885 (0.0107) 124.36 (0.67)

Ca1_$4 2.3582 (0.0113) 116.37 (0.52) 100.02 (0.43)

Ca1 2.4752 (0.0101) 106.64 (0.46) 108.50 (0.44) 97.68 (0.39)

O11 - Si6_$5 Al4 Ca1_$4

O12 - Distance Angles

B10 1.4483 (0.0209)

Al5 1.8321 (0.0112) 128.54 (0.91)

Al4 1.9641 (0.0117) 118.94 (0.98) 99.63 (0.52)

Ca2 2.6046 (0.0110) 108.25 (0.96) 98.31 (0.46) 97.28 (0.41)

O12 - B10 Al5 Al4

O13 - Distance Angles

Si9 1.5689 (0.0110)

Al4_$2 1.8818 (0.0113) 132.21 (0.72)

Ca2_$2 2.3438 (0.0118) 114.65 (0.58) 109.22 (0.47)

O13 - Si9 Al4_$2

O14 - Distance Angles

Si8_$3 1.6050 (0.0115)

Al4_$2 1.8728 (0.0117) 129.65 (0.64)

Ca1_$5 2.3698 (0.0112) 109.96 (0.60) 100.08 (0.45)

O14 - Si8_$3 Al4_$2

O15 - Distance Angles

Si8_$3 1.6227 (0.0112)

Al5_$3 1.9337 (0.0109) 121.74 (0.58)

Ca2_$3 2.3342 (0.0102) 122.82 (0.61) 104.97 (0.45)

O15 - Si8_$3 Al5_$3

O16 - Distance Angles

Si7_$9 1.6054 (0.0118)

Al4 1.8795 (0.0109) 131.54 (0.69)

X3_$8 2.0922 (0.0109) 118.45 (0.56) 106.83 (0.51)

O16 - Si7_$9 Al4

O17 - Distance Angles

B10 1.4810 (0.0230)

Si6_$5 1.6375 (0.0131) 133.28 (1.01)

X3 2.1245 (0.0106) 100.91 (0.99) 124.36 (0.59)

O17 - B10 Si6_$5

O18 - Distance Angles

B10_$6 1.4743 (0.0208)

Si7 1.6485 (0.0119) 119.91 (1.06)

Ca2_$3 2.4254 (0.0115) 99.23 (0.87) 136.53 (0.58)

O18 - B10_$6 Si7

O19 - Distance Angles

Si8 1.6494 (0.0125)

Si6_$3 1.6348 (0.0118) 143.20 (0.78)

Ca1_$1 2.8812 (0.0100) 88.59 (0.45) 90.52 (0.42)

Ca1_$3 3.0906 (0.0108) 81.19 (0.40) 132.65 (0.60) 110.91 (0.33)

O19 - Si8 Si6_$3 Ca1_$1

O20 - Distance Angles

Si9 1.6019 (0.0119)

Al5 1.9090 (0.0121) 133.24 (0.66)

Al5_$3 1.9210 (0.0110) 129.14 (0.68) 95.10 (0.52)

O20 - Si9 Al5

O21 - Distance Angles

Si6_$3 1.5896 (0.0120)

X3_$7 2.0921 (0.0108) 127.00 (0.74)

Ca2 2.3225 (0.0115) 123.31 (0.60) 103.36 (0.43)

O21 - Si6_$3 X3_$7

O22 - Distance Angles

Si8 1.5928 (0.0109)

Al5_$3 1.8547 (0.0112) 126.71 (0.69)

Ca1_$3 2.2301 (0.0119) 116.77 (0.58) 109.75 (0.48)

O22 - Si8 Al5_$3

O23 - Distance Angles

Si7_$3 1.6383 (0.0117)

Si9 1.6187 (0.0120) 142.36 (0.71)

Ca2_$2 2.8884 (0.0106) 127.22 (0.57) 90.37 (0.44)

O23 - Si7_$3 Si9

O24 - Distance Angles

Si7_$3 1.5665 (0.0111)

X3_$1 1.9695 (0.0108) 122.17 (0.62)

Ca1_$3 2.2888 (0.0105) 126.71 (0.58) 109.76 (0.47)

O24 - Si7_$3 X3_$1

O25 - Distance Angles

B10_$3 1.5257 (0.0220)

Si9_$3 1.6517 (0.0115) 131.74 (0.91)

Ca2_$6 2.4806 (0.0107) 95.48 (0.82) 119.00 (0.64)

O25 - B10_$3 Si9_$3

OH26 - Distance Angles

Al5 1.8731 (0.0132)

Al4 1.9578 (0.0116) 98.45 (0.57)

Ca1 2.5796 (0.0119) 96.21 (0.43) 102.42 (0.48)

OH26 - Al5 Al4

FMAP and GRID set by program

FMAP 2 1 15

GRID -4.167 -2 -2 4.167 2 2

R1 = 0.0648 for 614 unique reflections after merging for Fourier

Electron density synthesis with coefficients Fo-Fc

Highest peak 0.86 at 0.2066 0.9287 0.5858 [ 0.24 A from CA1 ]

Deepest hole -0.99 at 0.1365 0.0439 0.8793 [ 1.06 A from X3 ]

Mean = 0.00, Rms deviation from mean = 0.24 e/A^3, Highest memory used = 3757 / 11805

Fourier peaks appended to .res file

x y z sof U Peak Distances to nearest atoms (including symmetry equivalents)

Q1 1 0.7218 0.0912 0.1526 1.00000 0.05 0.84 0.38 O24 1.61 SI7 1.88 X3 2.50 CA1

Q2 1 0.3824 0.8543 0.1165 1.00000 0.05 0.72 0.80 O17 1.33 B10 1.80 SI6 1.89 O18

Q3 1 0.9073 0.3604 0.1169 1.00000 0.05 0.72 0.32 O23 1.71 SI7 1.79 SI9 2.42 O13

Q4 1 0.1771 0.7206 0.5443 1.00000 0.05 0.71 0.94 OH26 1.79 AL5 1.84 CA1 1.87 O22

Q5 1 0.7764 0.6153 0.6323 1.00000 0.05 0.70 0.55 O15 1.95 CA2 1.96 SI8 2.24 AL5

Q6 1 0.8632 0.5158 0.4773 1.00000 0.05 0.69 1.55 O15 1.71 OH26 1.87 AL5 2.06 O13

Q7 1 0.9838 0.5473 0.2186 1.00000 0.05 0.65 0.37 O13 1.47 SI9 2.11 AL4 2.17 CA2

Q8 1 0.5845 0.7746 0.2680 1.00000 0.05 0.64 1.64 O17 1.75 O25 1.81 B10 2.05 O22

Q9 1 0.7886 0.8210 0.3388 1.00000 0.05 0.60 0.35 O14 1.84 SI8 1.86 AL4 2.36 X3

Q10 1 0.3291 0.6570 0.2834 1.00000 0.05 0.59 0.34 O12 1.57 AL5 1.59 B10 1.99 AL4

Q11 1 0.3290 0.1255 0.1199 1.00000 0.05 0.59 0.68 O21 1.49 SI6 2.22 X3 2.33 O17

Q12 1 0.4595 0.0815 0.3721 1.00000 0.05 0.59 0.47 O19 1.67 SI8 1.98 SI6 2.31 O22

Q13 1 0.4420 0.5011 0.9645 1.00000 0.05 0.58 1.66 O18 1.91 O18 1.92 O25 2.09 B10

Q14 1 0.2292 0.6033 0.1907 1.00000 0.05 0.54 1.07 O12 1.82 AL4 1.85 O13 1.88 CA2

Q15 1 -0.0413 0.8881 0.1741 1.00000 0.05 0.53 1.04 O16 1.70 X3 1.83 AL4 2.09 O14

Q16 1 0.6250 0.5097 0.4263 1.00000 0.05 0.53 0.54 O20 1.50 AL5 1.94 AL5 1.94 SI9

Q17 1 0.2093 0.2258 0.2746 1.00000 0.05 0.52 1.51 O21 1.86 CA2 1.94 SI6 1.97 O19

Q18 1 0.8825 0.1013 0.1574 1.00000 0.05 0.51 0.79 O24 1.66 SI7 2.17 CA1 2.33 X3

Q19 1 0.8450 0.9731 0.7876 1.00000 0.05 0.51 1.13 SI6 1.31 O21 1.45 O11 2.37 O17

Q20 1 0.0486 0.9156 0.5694 1.00000 0.05 0.51 1.06 CA1 1.77 O11 2.02 O11 2.21 OH26

Shortest distances between peaks (including symmetry equivalents)

13 13 1.16 1 18 1.17 10 14 1.36 5 6 1.66 11 19 1.68 11 17 1.70 5 17 1.71

8 9 1.82 4 20 1.84 7 14 1.85 9 15 1.86 6 16 1.87 15 18 1.90 17 19 1.99

16 16 2.01 6 6 2.06 20 20 2.08 15 19 2.09 18 19 2.12 2 19 2.12 3 7 2.13

4 6 2.18 8 10 2.20 19 20 2.23 2 8 2.24 2 10 2.30 1 15 2.32 3 18 2.35

4 16 2.35 12 17 2.36 6 7 2.37 2 11 2.39 13 14 2.41 1 12 2.44 4 10 2.46

5 14 2.49 17 20 2.53 13 14 2.53 2 14 2.63 5 16 2.64 5 6 2.65 5 7 2.66

18 20 2.66 2 11 2.66 5 10 2.67 11 12 2.68 8 16 2.73 1 2 2.75 7 9 2.76

10 13 2.79 12 12 2.84 6 10 2.85 1 11 2.85 3 13 2.85 10 16 2.86 4 17 2.87

1 3 2.87 8 12 2.88 7 16 2.89 2 15 2.92 8 15 2.93 9 12 2.93 1 9 2.94

3 17 2.94 7 10 2.96 5 12 2.97 14 15 2.97 15 20 2.98 12 19 2.98 6 9 2.98

9 20 2.99

Time profile in seconds

-----------------------

0.03: Read and process instructions

0.00: Fit rigid groups

0.00: Interpret restraints etc.

0.00: Generate connectivity array

0.00: Analyse DFIX/DANG restraints

0.00: Analyse SAME/SADI restraints

0.00: Generate CHIV restraints

0.00: Check if bonds in residues restrained

0.00: Generate DELU restraints

0.00: Generate SIMU restraints

0.00: Generate ISOR restraints

0.00: Generate NCSY restraints

0.00: Analyse other restraints etc.

0.02: Read intensity data, sort/merge etc.

0.00: Set up constraints

0.00: OSF, H-atoms from difference map

0.00: Set up l.s. refinement

0.00: Generate idealized H-atoms

0.09: Structure factors and derivatives

0.05: Sum l.s. matrices

0.00: Generate and apply antibumping restraints

0.00: Apply other restraints

0.00: Solve l.s. equations

0.00: Generate HTAB table

0.00: Other dependent quantities, CIF, tables

0.02: Analysis of variance

0.00: Merge reflections for Fourier and .fcf

0.00: Fourier summations

0.00: Peaksearch

0.00: Analyse peaklist

+++++++++++++++++++++++++++++++++++++++++++++++++++++++++++++++++++++++++++++

+ axi_sca finished at 19:15:33 Total CPU time: 0.2 secs +

+++++++++++++++++++++++++++++++++++++++++++++++++++++++++++++++++++++++++++++

#

**# h,k,l, Fc-squared, Fo-squared, sigma(Fo-squared) and status flag**

**#**

**data_axi_sca**

**_shelx_title ' AXINITE (SUERT) P-1 ALBA:12_DES_14 UNIQUE_FILE with merged AX2'**

**_shelx_refln_list_code 4**

**_shelx_F_calc_maximum 154.12**

**_exptl_crystal_F_000 562.00**

**_reflns_d_resolution_high 1.0749**

**loop_**

**_symmetry_equiv_pos_as_xyz**

**'x, y, z'**

**'-x, -y, -z'**

**_cell_length_a 7.1233**

**_cell_length_b 8.8906**

**_cell_length_c 9.2070**

**_cell_angle_alpha 87.784**

**_cell_angle_beta 77.218**

**_cell_angle_gamma 81.651**

**_shelx_F_squared_multiplier 1.000**

**loop_**

**_refln_index_h**

**_refln_index_k**

**_refln_index_l**

**_refln_F_squared_calc**

**_refln_F_squared_meas**

**_refln_F_squared_sigma**

**_refln_observed_status**

4 0 0 1237.92 1184.79 44.93 o

5 0 0 1639.53 1705.28 58.62 o

6 0 0 266.43 232.85 28.83 o

-4 1 0 577.48 534.18 42.73 o

1 1 0 133.31 82.18 36.71 o

2 1 0 782.54 698.55 20.96 o

3 1 0 802.09 828.67 16.64 o

5 1 0 184.90 157.52 30.13 o

-5 2 0 403.71 335.58 10.34 o

2 2 0 604.84 410.91 38.83 o

3 2 0 636.83 534.18 42.73 o

4 2 0 41.10 61.64 31.37 o

5 2 0 285.03 280.79 34.79 o

0 3 0 131.39 184.91 28.70 o

1 3 0 2879.92 2554.49 40.82 o

3 3 0 5064.14 6718.39 21.02 o

4 3 0 1194.48 1075.22 46.09 o

5 3 0 23754.45 21826.20 9.79 o

-1 4 0 2044.85 2561.34 5.68 o

0 4 0 1639.52 2047.70 5.62 o

1 4 0 1958.85 1944.98 11.92 o

2 4 0 10734.65 12806.71 31.37 o

3 4 0 323.15 294.49 26.57 o

4 4 0 3109.70 3020.19 56.71 o

5 4 0 293.35 273.94 11.51 o

6 4 0 95.22 68.49 44.24 o

-1 5 0 1252.72 1260.13 1.58 o

1 5 0 1084.63 1109.46 55.95 o

2 5 0 191.83 116.42 61.29 o

3 5 0 1497.94 1492.97 6.44 o

4 5 0 267.53 260.24 33.15 o

5 5 0 306.94 205.46 48.15 o

-2 6 0 862.23 958.79 32.05 o

-1 6 0 284.69 164.36 57.05 o

0 6 0 3273.92 3472.19 50.75 o

2 6 0 3124.94 3218.80 37.46 o

4 6 0 656.06 719.09 55.47 o

5 6 0 495.77 362.97 51.36 o

-2 7 0 1471.12 1472.43 15.96 o

0 7 0 790.29 678.00 19.59 o

4 7 0 1364.72 1397.10 67.46 o

0 8 0 1874.14 1965.52 50.06 o

1 8 0 635.36 506.79 9.18 o

2 8 0 3694.96 4088.56 50.88 o

-2 -8 1 1558.58 1499.82 55.68 o

0 -8 1 1290.42 1725.82 54.31 o

-3 -7 1 348.40 328.73 43.15 o

-2 -7 1 1055.05 938.25 56.36 o

-1 -7 1 1012.88 1034.12 29.24 o

1 -7 1 2776.15 3157.16 63.49 o

2 -7 1 770.21 746.49 5.48 o

-3 -6 1 650.53 575.27 7.67 o

0 -6 1 4612.61 4780.26 49.99 o

1 -6 1 1814.10 1876.49 44.24 o

-3 -5 1 7893.63 9464.64 67.25 o

-2 -5 1 650.70 541.03 58.49 o

-1 -5 1 5878.73 6862.21 37.60 o

0 -5 1 239.14 212.30 6.16 o

1 -5 1 5146.62 6245.84 34.72 o

-5 -4 1 559.07 554.73 11.51 o

-4 -4 1 321.90 356.12 65.47 o

-2 -4 1 348.90 294.49 4.25 o

0 -4 1 416.20 362.97 25.00 o

1 -4 1 3697.38 4780.26 66.50 o

-6 -3 1 901.27 808.12 52.53 o

-5 -3 1 940.17 691.70 33.49 o

-3 -3 1 137.54 116.42 14.45 o

-2 -3 1 512.00 588.97 25.20 o

-1 -3 1 2818.50 2759.95 36.84 o

0 -3 1 805.47 910.85 5.27 o

-5 -2 1 804.15 917.70 1.64 o

-4 -2 1 623.61 575.27 10.07 o

-3 -2 1 2115.56 2164.13 25.54 o

-2 -2 1 2486.92 2335.34 67.12 o

-1 -2 1 780.23 787.58 58.83 o

0 -2 1 258.80 226.00 48.69 o

6 -2 1 2132.71 2643.52 46.71 o

-6 -1 1 93.29 109.58 53.97 o

-5 -1 1 192.02 143.82 46.02 o

-4 -1 1 175.94 150.67 28.97 o

-3 -1 1 3535.86 4184.44 44.45 o

-2 -1 1 6453.55 5965.05 49.24 o

-1 -1 1 826.50 814.97 8.29 o

0 -1 1 589.61 534.18 53.83 o

1 -1 1 436.09 308.18 24.65 o

2 -1 1 314.12 294.49 30.27 o

3 -1 1 2849.05 3225.65 20.00 o

4 -1 1 92.21 89.03 37.05 o

5 -1 1 485.20 486.24 68.35 o

6 -1 1 293.47 246.55 65.54 o

-6 0 1 4661.14 4841.90 20.55 o

-5 0 1 322.46 267.09 4.25 o

-2 0 1 8791.05 9629.00 49.10 o

-1 0 1 386.29 239.70 30.41 o

1 0 1 190.38 89.03 62.73 o

2 0 1 194.38 130.12 57.73 o

3 0 1 5175.25 5677.41 20.34 o

4 0 1 134.20 109.58 5.41 o

5 0 1 107.33 102.73 4.04 o

-4 1 1 1157.94 1164.25 30.06 o

-3 1 1 5197.46 7019.72 8.42 o

-2 1 1 2751.39 3232.50 12.40 o

0 1 1 1602.37 1780.61 41.43 o

2 1 1 1279.47 1520.37 43.15 o

3 1 1 676.48 541.03 30.75 o

5 1 1 392.68 356.12 21.78 o

6 1 1 176.78 157.52 7.33 o

-5 2 1 1676.53 1582.01 68.42 o

0 2 1 471.67 650.61 61.23 o

2 2 1 74.63 82.18 66.09 o

3 2 1 1160.08 828.67 46.57 o

4 2 1 2839.86 3568.07 4.25 o

5 2 1 232.93 232.85 5.27 o

-1 3 1 9530.66 12950.53 16.23 o

0 3 1 955.28 1191.64 47.73 o

2 3 1 710.18 698.55 16.64 o

4 3 1 497.63 595.82 15.07 o

6 3 1 275.89 239.70 9.59 o

-1 4 1 578.16 513.64 46.98 o

0 4 1 4974.31 7067.66 50.27 o

2 4 1 5076.44 6225.29 56.02 o

3 4 1 407.99 376.67 10.41 o

4 4 1 909.26 1068.37 51.09 o

5 4 1 212.13 212.30 44.58 o

-1 5 1 13342.39 16676.12 43.01 o

1 5 1 194.45 130.12 41.16 o

2 5 1 786.93 842.37 19.45 o

-2 6 1 712.29 773.88 56.77 o

-1 6 1 2327.71 3129.77 36.16 o

1 6 1 310.25 260.24 2.81 o

2 6 1 2159.84 2492.86 57.73 o

3 6 1 1102.28 1047.82 42.19 o

4 6 1 3594.47 3821.47 54.86 o

-2 7 1 967.08 1362.85 11.92 o

0 7 1 1152.39 1225.88 23.76 o

1 7 1 442.12 472.55 32.32 o

2 7 1 488.18 534.18 36.09 o

3 7 1 420.23 390.37 68.42 o

0 -7 2 310.94 315.03 53.69 o

1 -7 2 1468.94 1513.52 3.42 o

-4 -6 2 754.50 691.70 21.09 o

-3 -6 2 999.15 1116.31 23.70 o

-2 -6 2 387.62 404.06 26.50 o

-1 -6 2 2259.62 2314.80 3.36 o

1 -6 2 193.68 157.52 3.08 o

2 -6 2 716.15 712.24 5.41 o

3 -6 2 947.59 1198.49 30.20 o

-5 -5 2 2149.94 1986.07 12.88 o

-4 -5 2 328.80 280.79 58.42 o

-3 -5 2 1813.75 1986.07 39.86 o

-2 -5 2 354.95 342.43 17.94 o

-1 -5 2 1228.81 1321.76 13.22 o

0 -5 2 180.30 150.67 16.78 o

1 -5 2 2141.80 2198.37 25.48 o

3 -5 2 5034.88 5266.50 0.14 o

-5 -4 2 1396.46 1342.31 34.04 o

-4 -4 2 168.93 198.61 40.13 o

-3 -4 2 863.81 842.37 32.87 o

-2 -4 2 249.22 226.00 8.63 o

-1 -4 2 495.72 431.46 18.63 o

0 -4 2 2809.71 2903.77 59.72 o

2 -4 2 5715.40 5389.78 3.42 o

3 -4 2 432.11 362.97 64.79 o

5 -4 2 235.95 191.76 23.08 o

-5 -3 2 158.22 164.36 24.45 o

-4 -3 2 117.80 109.58 4.25 o

-3 -3 2 251.93 226.00 37.32 o

-2 -3 2 488.21 376.67 5.62 o

0 -3 2 1362.05 1445.04 65.81 o

1 -3 2 199.46 294.49 36.50 o

2 -3 2 1988.99 1164.25 63.14 o

4 -3 2 1524.00 1499.82 14.66 o

-5 -2 2 1935.29 1924.43 25.41 o

-4 -2 2 100.36 68.49 40.13 o

-3 -2 2 148.94 143.82 61.02 o

-2 -2 2 1568.66 849.22 48.01 o

1 -2 2 1519.14 1438.19 36.78 o

2 -2 2 1986.58 1239.58 39.04 o

3 -2 2 370.43 376.67 18.15 o

4 -2 2 245.71 239.70 10.00 o

5 -2 2 253.99 212.30 56.50 o

-5 -1 2 331.99 273.94 49.79 o

-3 -1 2 283.82 184.91 35.41 o

-1 -1 2 4712.03 5506.20 23.90 o

0 -1 2 268.03 246.55 33.69 o

1 -1 2 2998.91 3663.95 6.78 o

2 -1 2 4164.73 4280.32 64.92 o

3 -1 2 1900.79 1650.49 36.50 o

4 -1 2 4718.47 5355.53 45.75 o

5 -1 2 2982.00 3348.92 53.76 o

6 -1 2 124.99 95.88 45.20 o

-5 0 2 1115.46 1109.46 51.43 o

-4 0 2 3691.26 4095.41 12.53 o

-3 0 2 540.24 404.06 67.32 o

-2 0 2 256.25 212.30 29.79 o

-1 0 2 5041.78 5766.44 43.35 o

0 0 2 63.59 68.49 5.07 o

2 0 2 106.89 95.88 36.71 o

3 0 2 204.69 178.06 57.60 o

4 0 2 338.04 315.03 39.31 o

5 0 2 158.25 191.76 50.13 o

6 0 2 89.45 82.18 31.30 o

-4 1 2 2706.15 2931.16 0.48 o

-1 1 2 67.25 75.33 0.68 o

1 1 2 83.16 75.33 67.53 o

3 1 2 348.61 178.06 56.98 o

4 1 2 77.53 54.79 54.03 o

5 1 2 5158.47 6355.42 44.52 o

-2 2 2 188.53 232.85 32.87 o

-1 2 2 3052.74 3561.22 9.45 o

0 2 2 411.69 472.55 12.19 o

1 2 2 263.05 246.55 16.16 o

2 2 2 652.66 602.67 51.50 o

3 2 2 664.84 630.06 48.83 o

6 2 2 583.24 534.18 15.00 o

-5 3 2 4166.18 5650.02 0.27 o

-4 3 2 562.23 602.67 8.29 o

0 3 2 372.45 383.52 19.66 o

1 3 2 1631.07 1547.76 44.99 o

2 3 2 41.09 47.94 43.56 o

3 3 2 76.36 54.79 6.92 o

4 3 2 3841.73 6355.42 15.75 o

5 3 2 358.05 390.36 38.35 o

-1 4 2 707.65 890.31 55.27 o

0 4 2 1285.69 1499.82 21.23 o

1 4 2 4796.30 6499.23 66.23 o

4 4 2 519.64 452.00 68.21 o

5 4 2 1917.67 1890.19 55.95 o

0 5 2 294.04 349.27 21.09 o

2 5 2 835.24 719.09 3.01 o

3 5 2 481.28 404.06 57.87 o

4 5 2 2025.72 2177.83 36.16 o

-2 6 2 1530.89 1705.28 14.66 o

-1 6 2 2557.52 3068.13 62.32 o

0 6 2 4229.59 5198.02 45.82 o

2 6 2 8052.88 10608.34 49.17 o

4 6 2 983.57 1061.52 50.06 o

1 7 2 382.24 321.88 31.43 o

1 8 2 461.45 397.21 3.70 o

2 8 2 437.70 438.30 35.34 o

-1 -7 3 212.02 184.91 4.73 o

0 -7 3 507.39 506.79 27.39 o

2 -7 3 153.02 171.21 44.24 o

-3 -6 3 185.47 150.67 36.84 o

-2 -6 3 151.50 164.36 60.47 o

-1 -6 3 944.03 1047.82 44.31 o

1 -6 3 3262.24 3732.44 59.45 o

2 -6 3 4878.58 4951.47 67.73 o

-4 -5 3 1766.94 1547.76 56.23 o

-3 -5 3 673.16 719.09 32.67 o

-2 -5 3 586.84 527.34 16.57 o

-1 -5 3 6437.51 8362.03 55.75 o

2 -5 3 954.28 787.58 59.86 o

3 -5 3 2273.31 2876.37 0.82 o

-5 -4 3 164.07 109.58 27.80 o

-4 -4 3 833.38 773.88 31.91 o

-2 -4 3 121.37 89.03 56.84 o

-1 -4 3 460.89 321.88 51.23 o

0 -4 3 2086.92 2554.49 48.90 o

1 -4 3 228.12 246.55 24.52 o

2 -4 3 1098.20 1027.28 44.79 o

5 -4 3 213.97 178.06 36.50 o

-5 -3 3 1127.76 993.03 58.01 o

-4 -3 3 869.26 753.34 66.16 o

-3 -3 3 3430.23 3369.47 59.03 o

-2 -3 3 2425.53 2698.31 36.43 o

-1 -3 3 179.77 171.21 33.56 o

1 -3 3 321.06 376.67 60.34 o

2 -3 3 145.80 143.82 58.14 o

4 -3 3 2261.59 2595.58 32.39 o

-5 -2 3 196.68 219.15 54.65 o

-4 -2 3 5915.18 5855.47 15.89 o

-3 -2 3 148.99 143.82 42.46 o

-2 -2 3 7743.50 9204.40 3.01 o

-1 -2 3 2231.16 2143.58 65.54 o

0 -2 3 754.20 773.88 55.47 o

1 -2 3 2105.59 1773.76 51.71 o

2 -2 3 179.01 157.52 66.23 o

4 -2 3 6061.10 7567.60 45.95 o

5 -2 3 304.22 267.09 43.69 o

6 -2 3 173.46 109.58 18.15 o

-4 -1 3 230.26 226.00 22.46 o

-3 -1 3 397.70 356.12 24.52 o

-2 -1 3 3345.01 3013.34 17.81 o

1 -1 3 7305.61 9478.34 59.45 o

2 -1 3 227.41 171.21 20.00 o

3 -1 3 3053.85 3800.92 10.14 o

4 -1 3 1356.49 1890.19 65.54 o

5 -1 3 603.80 684.85 36.84 o

-5 0 3 319.43 205.46 20.82 o

-3 0 3 803.83 691.70 60.27 o

-1 0 3 619.88 520.49 14.38 o

0 0 3 3615.92 4129.65 10.48 o

1 0 3 8028.74 9040.03 1.16 o

2 0 3 4814.09 5567.84 63.21 o

3 0 3 2387.66 2780.49 62.05 o

4 0 3 2091.90 2513.40 67.66 o

5 0 3 287.68 294.49 61.09 o

-5 1 3 693.41 643.76 4.66 o

-3 1 3 4475.34 4403.59 66.98 o

-2 1 3 460.79 486.24 46.43 o

-1 1 3 11327.95 11108.28 17.81 o

1 1 3 16837.99 10471.37 31.85 o

3 1 3 9516.85 11834.22 30.27 o

6 1 3 1447.89 1321.76 19.86 o

-5 2 3 445.93 506.79 57.32 o

-1 2 3 105.48 116.42 54.45 o

0 2 3 1803.42 1821.70 39.65 o

1 2 3 626.17 452.00 57.73 o

2 2 3 660.32 630.06 37.67 o

3 2 3 1675.18 1534.07 51.98 o

4 2 3 413.02 417.76 23.08 o

5 2 3 4434.47 5047.35 0.68 o

6 2 3 2197.48 2239.46 1.10 o

-3 3 3 1449.23 1739.52 42.05 o

-1 3 3 2106.11 2232.61 28.56 o

2 3 3 181.04 178.06 3.56 o

4 3 3 328.02 321.88 0.27 o

5 3 3 126.97 143.82 27.60 o

6 3 3 1897.32 1780.61 52.87 o

-4 4 3 425.61 561.58 44.17 o

-3 4 3 635.00 732.79 61.16 o

-1 4 3 1778.33 1883.34 39.93 o

0 4 3 1159.37 1102.61 59.45 o

2 4 3 1654.15 1328.61 64.10 o

3 4 3 656.30 609.52 45.82 o

4 4 3 8263.68 8457.91 32.05 o

5 4 3 285.05 246.55 65.40 o

6 4 3 2333.45 2335.34 44.45 o

0 5 3 3292.67 3643.41 28.97 o

1 5 3 349.41 267.09 42.87 o

2 5 3 1362.65 1410.79 42.05 o

4 5 3 105.93 109.58 66.77 o

5 5 3 1789.87 2157.28 4.31 o

-2 6 3 4537.51 5643.17 46.09 o

-1 6 3 1412.79 1540.91 12.67 o

2 6 3 3457.49 4020.07 49.04 o

3 6 3 1417.21 1520.37 14.04 o

4 6 3 1472.54 1369.70 66.09 o

5 6 3 306.04 356.12 0.14 o

-1 7 3 1724.61 2061.40 16.03 o

1 7 3 371.68 397.21 54.93 o

3 7 3 597.71 657.46 46.64 o

4 7 3 261.22 253.39 47.32 o

0 -7 4 1822.97 1910.73 52.80 o

1 -7 4 3423.77 3835.16 6.57 o

-3 -6 4 2892.75 2944.86 19.38 o

-2 -6 4 4089.61 4629.59 44.31 o

-1 -6 4 959.07 917.70 42.12 o

0 -6 4 299.56 294.49 6.92 o

2 -6 4 219.96 191.76 34.45 o

-3 -5 4 753.46 794.43 14.24 o

-2 -5 4 682.78 636.91 43.69 o

-1 -5 4 12216.33 17258.24 19.59 o

0 -5 4 970.42 965.64 36.30 o

1 -5 4 2688.67 3033.89 67.59 o

2 -5 4 2160.50 2232.61 57.12 o

3 -5 4 1414.20 1376.55 23.35 o

-4 -4 4 4315.54 3972.14 25.48 o

-3 -4 4 524.68 465.70 22.19 o

-2 -4 4 2925.15 2958.56 50.61 o

0 -4 4 5989.35 7232.03 54.10 o

1 -4 4 312.98 301.33 8.42 o

2 -4 4 310.99 308.18 7.60 o

3 -4 4 188.86 157.52 4.86 o

-4 -3 4 260.61 280.79 63.62 o

-3 -3 4 1282.19 1109.46 13.35 o

-2 -3 4 184.98 123.27 40.54 o

-1 -3 4 193.01 150.67 15.41 o

1 -3 4 751.70 527.34 8.90 o

2 -3 4 10172.72 12628.65 44.58 o

3 -3 4 5665.83 6191.05 44.24 o

4 -3 4 3019.75 3095.53 50.47 o

5 -3 4 540.84 486.24 6.64 o

-4 -2 4 225.10 164.36 28.08 o

-3 -2 4 189.95 123.27 30.89 o

-2 -2 4 710.70 588.97 66.43 o

0 -2 4 3807.62 4424.14 13.22 o

1 -2 4 298.56 280.79 59.38 o

3 -2 4 1146.43 1225.88 18.76 o

4 -2 4 12013.97 10991.86 14.38 o

5 -2 4 974.14 1198.49 42.87 o

-4 -1 4 154.53 130.12 9.11 o

0 -1 4 5666.35 6718.39 16.09 o

2 -1 4 984.20 869.76 14.18 o

3 -1 4 2106.89 2266.86 24.38 o

4 -1 4 1246.38 1369.70 62.80 o

5 -1 4 2468.21 2965.40 40.82 o

-4 0 4 196.53 191.76 17.19 o

-3 0 4 5366.72 5129.53 62.46 o

-2 0 4 9184.75 10834.34 43.35 o

-1 0 4 2896.82 2396.98 4.11 o

0 0 4 3018.37 3116.07 3.56 o

1 0 4 1201.06 1047.82 32.05 o

3 0 4 352.76 321.88 19.31 o

4 0 4 796.58 1082.06 20.20 o

5 0 4 106.73 130.12 38.01 o

6 0 4 144.81 116.42 3.15 o

-3 1 4 7096.59 8067.54 60.13 o

-1 1 4 303.34 143.82 64.24 o

1 1 4 444.63 404.06 22.12 o

2 1 4 529.02 527.34 61.36 o

3 1 4 457.23 404.06 62.73 o

4 1 4 149.78 150.67 18.29 o

-3 2 4 209.75 212.30 16.98 o

-2 2 4 927.40 1061.52 41.98 o

-1 2 4 5300.25 5999.29 38.83 o

1 2 4 304.80 239.70 65.95 o

2 2 4 631.09 547.88 10.55 o

4 2 4 2262.81 2191.52 45.54 o

5 2 4 324.64 308.18 3.70 o

6 2 4 813.47 739.64 46.30 o

0 3 4 3492.36 4013.23 14.11 o

1 3 4 240.48 212.30 23.56 o

2 3 4 11985.55 16217.27 7.33 o

3 3 4 4884.14 4451.53 48.49 o

4 3 4 5661.84 6492.39 30.96 o

5 3 4 624.95 623.21 2.47 o

6 3 4 856.69 814.97 42.94 o

-3 4 4 2171.80 2492.86 43.35 o

-2 4 4 2866.54 3253.04 42.67 o

-1 4 4 626.58 705.40 5.48 o

0 4 4 18342.29 27757.01 11.03 o

1 4 4 662.02 561.58 41.30 o

2 4 4 14478.64 17087.03 26.91 o

3 4 4 431.43 342.43 24.86 o

4 4 4 1173.49 1034.12 45.13 o

0 5 4 338.70 308.18 55.88 o

1 5 4 357.39 342.43 8.15 o

2 5 4 724.45 725.94 68.07 o

3 5 4 381.91 369.82 8.22 o

4 5 4 987.60 739.64 54.79 o

-2 6 4 1603.80 2006.61 56.29 o

0 6 4 1129.57 1294.37 34.93 o

1 6 4 637.57 664.31 45.41 o

2 6 4 186.45 136.97 38.63 o

2 7 4 465.88 438.30 0.75 o

3 7 4 992.96 1164.25 65.20 o

0 -6 5 700.25 760.18 31.23 o

2 -6 5 44.61 41.09 1.30 o

-3 -5 5 242.47 184.91 23.76 o

-2 -5 5 2431.46 2855.83 62.05 o

-1 -5 5 422.84 397.21 10.34 o

-3 -4 5 364.42 315.03 52.60 o

-2 -4 5 1107.51 1027.28 63.49 o

-1 -4 5 954.22 904.00 44.24 o

1 -4 5 851.49 917.70 46.16 o

2 -4 5 351.96 321.88 8.08 o

4 -4 5 725.61 760.18 66.29 o

-3 -3 5 300.65 280.79 36.64 o

-2 -3 5 4897.28 4746.02 63.83 o

-1 -3 5 2452.17 2506.55 2.05 o

0 -3 5 4798.87 5848.63 14.11 o

1 -3 5 3493.41 3588.62 44.58 o

2 -3 5 115.22 116.42 22.67 o

3 -3 5 433.70 452.00 36.84 o

5 -3 5 2552.17 2609.28 57.66 o

-4 -2 5 470.71 362.97 12.19 o

-3 -2 5 393.01 342.43 47.19 o

-1 -2 5 3470.98 2554.49 19.31 o

0 -2 5 12285.83 15210.54 22.46 o

1 -2 5 1580.91 1650.49 37.39 o

2 -2 5 922.54 965.64 15.27 o

3 -2 5 4285.42 4211.83 21.23 o

5 -2 5 214.84 171.21 18.70 o

-4 -1 5 1267.81 1417.64 40.68 o

-3 -1 5 7065.92 7485.42 3.84 o

-1 -1 5 1157.76 1171.09 23.28 o

0 -1 5 461.99 362.97 23.28 o

1 -1 5 464.86 335.58 58.76 o

2 -1 5 2168.13 2301.10 17.87 o

3 -1 5 2182.90 2191.52 34.38 o

-4 0 5 1117.07 1814.85 33.28 o

-2 0 5 4673.69 4184.44 47.80 o

-1 0 5 954.07 890.31 27.46 o

0 0 5 313.29 232.85 27.46 o

1 0 5 305.84 335.58 48.28 o

2 0 5 223.92 136.97 60.34 o

3 0 5 2784.68 2848.98 43.76 o

4 0 5 2586.33 2938.01 9.66 o

5 0 5 6277.63 6827.96 67.25 o

6 0 5 897.98 986.19 67.46 o

-2 1 5 1612.45 1636.79 55.20 o

1 1 5 818.74 739.64 60.40 o

2 1 5 1632.86 1671.04 67.73 o

3 1 5 191.77 164.36 8.97 o

4 1 5 1500.25 1410.79 0.75 o

5 1 5 5164.83 5930.81 52.80 o

6 1 5 536.70 582.12 55.47 o

-3 2 5 663.90 636.91 13.49 o

-2 2 5 683.13 732.79 19.86 o

-1 2 5 1006.67 945.09 7.60 o

0 2 5 523.75 479.40 32.05 o

1 2 5 1743.18 1424.49 59.03 o

2 2 5 3463.35 3540.68 22.81 o

3 2 5 501.05 404.06 17.33 o

4 2 5 1539.61 1424.49 68.42 o

5 2 5 1247.36 1205.34 47.46 o

-2 3 5 4111.39 5341.84 32.19 o

0 3 5 5350.37 5910.26 45.54 o

2 3 5 390.08 410.91 66.16 o

3 3 5 1453.85 1150.55 7.19 o

5 3 5 3001.09 3348.92 68.21 o

-2 4 5 4380.44 4800.80 2.81 o

0 4 5 744.47 739.64 6.99 o

3 4 5 2419.59 2328.49 55.13 o

4 4 5 622.91 547.88 25.13 o

5 4 5 105.68 95.88 36.91 o

0 5 5 312.05 308.18 60.47 o

1 5 5 443.21 410.91 42.53 o

2 5 5 723.74 664.31 67.46 o

4 5 5 334.72 280.79 55.27 o

-1 6 5 295.72 356.12 51.16 o

1 6 5 2674.46 3184.56 31.37 o

3 6 5 1143.16 1280.67 63.35 o

0 -5 6 490.87 493.09 39.52 o

1 -5 6 1236.94 1164.25 53.69 o

2 -5 6 1507.95 1417.64 48.69 o

-2 -4 6 310.37 212.30 63.83 o

0 -4 6 645.59 588.97 64.92 o

-3 -3 6 386.22 376.67 34.45 o

-1 -3 6 1149.55 1171.09 0.07 o

1 -3 6 1720.80 1595.70 1.16 o

3 -3 6 238.56 178.06 38.97 o

4 -3 6 248.92 232.85 33.56 o

-3 -2 6 2005.85 1725.82 56.91 o

-1 -2 6 3260.24 2931.16 59.31 o

0 -2 6 1376.82 1445.04 67.46 o

1 -2 6 212.74 184.91 21.78 o

3 -2 6 610.86 513.64 2.12 o

-2 -1 6 932.15 924.55 48.56 o

0 -1 6 469.76 376.67 57.87 o

1 -1 6 167.96 157.52 4.45 o

2 -1 6 3027.05 3780.38 24.31 o

5 -1 6 632.15 678.00 40.89 o

-2 0 6 1224.90 1458.73 10.68 o

-1 0 6 4395.87 4191.29 31.30 o

0 0 6 475.54 349.27 42.19 o

1 0 6 1216.95 1301.22 20.13 o

2 0 6 2939.20 2732.55 65.95 o

4 0 6 2111.21 2301.10 27.87 o

-2 1 6 278.46 198.61 45.34 o

0 1 6 2152.21 2088.80 7.81 o

1 1 6 1023.20 979.34 1.71 o

2 1 6 2428.95 2403.83 33.56 o

3 1 6 490.90 554.73 32.32 o

4 1 6 1593.80 1506.67 34.79 o

5 1 6 254.14 157.52 48.42 o

-3 2 6 1929.51 2081.95 38.42 o

-2 2 6 965.43 1034.12 38.97 o

0 2 6 3187.58 3061.28 29.11 o

1 2 6 125.97 109.58 62.32 o

3 2 6 1709.38 1232.73 43.97 o

-1 3 6 4619.40 4711.77 48.56 o

0 3 6 377.96 335.58 59.72 o

1 3 6 454.08 328.73 49.38 o

2 3 6 178.47 136.97 20.00 o

3 3 6 2173.62 2040.86 45.68 o

4 3 6 1905.23 1725.82 10.00 o

5 3 6 1149.09 1143.70 55.68 o

-1 4 6 1622.28 1657.34 8.08 o

1 4 6 2627.04 2807.89 47.87 o

3 4 6 1752.15 1568.31 25.82 o

0 5 6 431.32 417.76 16.16 o

2 5 6 1014.16 1102.61 46.84 o

3 5 6 349.52 335.58 59.24 o

4 5 6 966.93 924.55 64.79 o

0 -4 7 3853.55 4013.23 57.60 o

1 -4 7 336.59 287.64 29.24 o

2 -4 7 2223.02 2143.58 25.34 o

1 -3 7 212.17 198.61 51.57 o

2 -3 7 540.30 513.64 62.94 o

-1 -2 7 524.61 541.03 34.38 o

1 -2 7 1025.08 1027.28 3.15 o

2 -2 7 1525.65 1486.13 4.66 o

3 -2 7 1549.95 1718.98 64.72 o

4 -2 7 895.02 856.06 62.12 o

2 -1 7 298.40 260.24 1.23 o

3 -1 7 706.25 691.70 51.57 o

4 -1 7 1436.47 1465.58 51.50 o

-2 0 7 607.17 609.52 28.28 o

-1 0 7 752.73 630.06 51.09 o

1 0 7 990.45 876.61 62.25 o

4 0 7 988.60 979.34 23.56 o

-2 1 7 3032.05 2766.80 38.90 o

0 1 7 2319.41 2184.67 28.63 o

1 1 7 525.04 513.64 43.90 o

4 1 7 294.31 541.03 51.57 o

-1 2 7 548.29 513.64 45.95 o

2 2 7 581.05 547.88 21.16 o

-1 3 7 1970.52 1828.55 26.78 o

0 3 7 1732.54 1814.85 61.91 o

1 3 7 403.74 356.12 21.16 o

2 3 7 2833.30 2629.83 10.34 o

3 3 7 1383.33 1390.25 14.11 o

4 3 7 2082.75 1958.67 31.23 o

1 4 7 3591.60 3732.44 1.78 o

2 4 7 1629.82 1513.52 15.68 o

3 4 7 2176.14 2102.49 40.54 o

1 -2 8 458.54 342.43 57.19 o

0 -1 8 1020.91 1075.22 31.91 o

1 -1 8 2991.08 2862.68 10.75 o

2 -1 8 1652.64 1506.67 59.24 o

3 -1 8 1765.58 1650.49 59.38 o

0 0 8 1988.33 1808.01 11.78 o

1 0 8 437.17 356.12 22.12 o

2 0 8 137.14 123.27 15.14 o

3 0 8 1667.83 1732.67 19.86 o

0 1 8 654.30 650.61 17.60 o

2 1 8 1542.84 1527.22 1.03 o

3 1 8 831.71 1088.91 27.60 o

1 2 8 358.06 383.52 22.12 o

3 2 8 552.60 458.85 47.46 o

2 3 8 3784.44 4211.83 35.27 o
