# Supplementary material for: Application of synchrotron through-the-substrate microdiffraction to crystals in polished thin sections
Source: IUCrJ. 2015 Jun 11;2(Pt 4):452–63. doi: 10.1107/S2052252515007794 (PMC4491317; doi:10.1107/S2052252515007794)
Supplement: Supplementary file 1 [file m-02-00452-sup1.zip › Suppl_files_Rius/Diopside_merging&scaling&LS/Suppl_Diopside.docx]

**Diopside – Multicrystal merging**

**J.Rius ICMAB_CSIC 2014**

CRYSTAL DATA:

=============

DIOPSID MSPD 25-01-14 + 3-12-14 C2/C cella LS (diops1,2,4,8)

A= 9.735 B= 8.911 C= 5.245

ALPHA= 90.00 BETA= 106.39 GAMMA= 90.00

VOLUME (A3)= 436.54

(SINT/L)2= 0.002866*H2 + 0.003148*K2 + 0.009873*L2 +

0.000000*HK + 0.003001*HL + 0.000000*KL

BRAVAIS LATTICE IS C CENTRED AT:

1) 0.000000 0.000000 0.000000

2) 0.500000 0.500000 0.000000

LAUE SYMMETRY OPERATIONS:

R11 R12 R13 R21 R22 R23 R31 R32 R33 T1 T2 T3

1) 1 0 0 0 1 0 0 0 1 0.00 0.00 0.00

2) 1 0 0 0 -1 0 0 0 1 0.00 0.00 0.00

TYPE OF RADIATION IS X-RAYS

UNIT CELL CONTENTS

SYMBOL ATOMIC_NUMBER NUMBER IN CELL TYPE SCAT_POWER

CA 20 4 1 20.00

SI 14 7 2 14.00

AL 13 2 3 13.00

MG 12 3 4 12.00

O 8 24 5 8.00

DSMIN OF INPUT REFLECTIONS IS: 1.0452 ANGS

RESIDUAL: 0.0302

------------------------------------------------------------------------------

FACTOR DE ESCALA (F"S) N.PATRO NOM_PATRO

1.0284184 1 DIOPS1_01.HKL

1.0187055 2 DIOPS2_01.HKL

1.0064324 3 DIOPS4_01.HKL

0.9442927 4 DIOPS8_01.HKL

------------------------------------------------------------------------------

MATRIU DE CORRELACIO:

37 16 3 15

16 41 10 21

3 10 44 18

15 21 18 56

------------------------------------------------------------------------------

MATRIU DE RESIDUALS:

0.0000 0.0089 0.0032 0.0620

0.0089 0.0000 0.0253 0.0399

0.0032 0.0253 0.0000 0.0259

0.0620 0.0399 0.0259 0.0000

------------------------------------------------------------------------------

HKL F2AV DF2AV N.CONT F2SCA(IMAG=1), F2SCA(IMAG=2)... FINS NIMAG:

========================================================================

2 0 0 61.185 7.822 1 0.000 0.000 61.185 0.000

4 0 0 54.120 7.357 1 0.000 0.000 54.120 0.000

6 0 0 2460.410 49.603 1 0.000 0.000 2460.410 0.000

1 1 0 3.790 1.947 1 0.000 0.000 0.000 3.790

5 1 0 1251.810 35.381 1 0.000 0.000 1251.810 0.000

7 1 0 575.716 23.994 1 0.000 0.000 575.716 0.000

0 2 0 143.073 11.961 1 143.073 0.000 0.000 0.000

2 2 0 1597.851 39.973 1 0.000 1597.851 0.000 0.000

6 2 0 118.343 10.879 1 0.000 0.000 118.343 0.000

8 2 0 249.919 15.809 1 0.000 0.000 249.919 0.000

1 3 0 33.961 5.828 1 33.961 0.000 0.000 0.000

3 3 0 1094.838 33.088 1 0.000 1094.838 0.000 0.000

2 4 0 156.588 12.514 1 0.000 156.588 0.000 0.000

4 4 0 1262.426 35.531 1 0.000 1262.426 0.000 0.000

6 4 0 226.730 15.058 1 0.000 226.730 0.000 0.000

1 5 0 3146.242 56.091 2 3010.887 3281.597 0.000 0.000

3 5 0 1688.732 41.094 2 0.000 1490.401 0.000 1887.063

0 6 0 4441.758 66.647 1 4441.758 0.000 0.000 0.000

2 6 0 182.502 13.509 2 184.485 180.518 0.000 0.000

4 6 0 143.657 11.986 1 0.000 143.657 0.000 0.000

3 7 0 41.223 6.420 1 0.000 0.000 0.000 41.223

2 8 0 143.706 11.988 2 128.594 158.819 0.000 0.000

-7 1 1 479.398 21.895 1 0.000 0.000 479.398 0.000

-5 1 1 60.957 7.808 3 0.000 62.100 76.393 44.379

-3 1 1 2286.654 47.819 3 0.000 2413.604 2092.836 2353.523

-1 1 1 38.093 6.172 1 0.000 0.000 0.000 38.093

3 1 1 2054.589 45.328 1 0.000 0.000 2054.589 0.000

7 1 1 246.982 15.716 1 0.000 0.000 246.982 0.000

-8 2 1 513.229 22.655 1 0.000 0.000 513.229 0.000

-6 2 1 576.656 24.014 3 0.000 571.568 635.426 522.975

-4 2 1 1414.645 37.612 3 0.000 1540.162 1286.300 1417.473

-2 2 1 7430.548 86.201 1 0.000 0.000 0.000 7430.548

0 2 1 522.291 22.854 2 600.181 0.000 0.000 444.400

2 2 1 3724.983 61.033 1 0.000 0.000 3724.983 0.000

4 2 1 261.806 16.180 1 0.000 0.000 261.806 0.000

6 2 1 1198.390 34.618 1 0.000 0.000 1198.390 0.000

-7 3 1 654.927 25.592 2 0.000 647.210 662.643 0.000

-5 3 1 5185.594 72.011 1 0.000 0.000 5185.594 0.000

-1 3 1 2648.990 51.468 3 2409.451 2663.320 0.000 2874.198

1 3 1 118.284 10.876 1 0.000 118.284 0.000 0.000

5 3 1 6428.348 80.177 1 0.000 0.000 6428.348 0.000

-2 4 1 166.529 12.905 1 0.000 166.529 0.000 0.000

0 4 1 2550.307 50.501 3 2894.548 2746.839 0.000 2009.536

2 4 1 218.721 14.789 2 0.000 210.738 0.000 226.703

-1 5 1 200.561 14.162 2 189.366 211.755 0.000 0.000

3 5 1 217.148 14.736 2 0.000 194.539 0.000 239.757

5 5 1 73.511 8.574 1 0.000 0.000 0.000 73.511

-6 6 1 193.293 13.903 1 0.000 193.293 0.000 0.000

-4 6 1 313.518 17.706 1 0.000 313.518 0.000 0.000

0 6 1 670.569 25.895 3 716.681 658.584 0.000 636.443

2 6 1 209.743 14.483 2 213.792 205.695 0.000 0.000

-1 7 1 491.298 22.165 2 496.374 486.222 0.000 0.000

1 7 1 899.394 29.990 3 825.872 987.139 0.000 885.170

0 8 1 164.044 12.808 2 170.688 0.000 0.000 157.401

-8 0 2 293.528 17.133 2 0.000 0.000 352.917 234.140

-6 0 2 2953.504 54.346 1 0.000 0.000 0.000 2953.504

-4 0 2 3414.632 58.435 1 0.000 0.000 0.000 3414.632

-9 1 2 199.203 14.114 1 0.000 0.000 0.000 199.203

-7 1 2 2752.358 52.463 2 0.000 0.000 3048.149 2456.566

-5 1 2 331.820 18.216 1 0.000 0.000 0.000 331.820

5 1 2 413.681 20.339 1 0.000 0.000 413.681 0.000

-8 2 2 274.926 16.581 2 0.000 0.000 329.427 220.425

-6 2 2 862.554 29.369 3 0.000 940.538 784.790 862.334

-4 2 2 102.666 10.132 3 0.000 99.324 112.524 96.151

-2 2 2 147.000 12.124 2 0.000 0.000 141.057 152.942

0 2 2 1259.401 35.488 1 1259.401 0.000 0.000 0.000

2 2 2 807.783 28.422 1 0.000 0.000 807.783 0.000

4 2 2 309.453 17.591 1 0.000 0.000 309.453 0.000

6 2 2 265.614 16.298 1 0.000 0.000 265.614 0.000

-5 3 2 275.429 16.596 3 0.000 337.376 194.468 294.444

-1 3 2 1011.120 31.798 2 1099.913 0.000 0.000 922.327

1 3 2 530.514 23.033 1 530.514 0.000 0.000 0.000

-6 4 2 57.430 7.578 1 0.000 57.430 0.000 0.000

-2 4 2 282.866 16.819 4 291.296 228.266 302.332 309.568

0 4 2 999.417 31.614 2 1109.036 0.000 0.000 889.798

2 4 2 750.401 27.393 1 0.000 0.000 750.401 0.000

4 4 2 258.220 16.069 1 0.000 0.000 258.220 0.000

-7 5 2 2261.973 47.560 1 0.000 0.000 0.000 2261.973

-3 5 2 3279.036 57.263 2 0.000 2853.178 0.000 3704.895

-1 5 2 90.535 9.515 2 91.285 0.000 0.000 89.784

1 5 2 1439.640 37.943 2 1298.925 1580.354 0.000 0.000

-2 6 2 1133.883 33.673 3 1254.462 1028.940 0.000 1118.249

0 6 2 3106.870 55.739 2 3035.514 3178.226 0.000 0.000

4 6 2 166.202 12.892 1 0.000 0.000 0.000 166.202

-1 7 2 417.270 20.427 2 478.489 0.000 0.000 356.051

-7 1 3 399.708 19.993 1 0.000 0.000 0.000 399.708

-5 1 3 172.675 13.141 1 0.000 0.000 0.000 172.675

-8 2 3 1060.370 32.563 2 0.000 0.000 1079.849 1040.890

-4 2 3 168.110 12.966 1 0.000 0.000 0.000 168.110

-7 3 3 170.708 13.066 2 0.000 0.000 177.714 163.701

-5 3 3 1495.932 38.677 3 0.000 1321.858 1544.348 1621.589

-3 3 3 303.110 17.410 2 0.000 0.000 301.147 305.073

1 3 3 379.243 19.474 2 381.989 0.000 376.497 0.000

-6 4 3 423.998 20.591 1 0.000 0.000 0.000 423.998

-4 4 3 327.076 18.085 2 0.000 299.705 0.000 354.446

-2 4 3 674.883 25.979 2 684.968 0.000 0.000 664.798

0 4 3 640.843 25.315 1 640.843 0.000 0.000 0.000

-5 5 3 318.796 17.855 1 0.000 0.000 0.000 318.796

-3 5 3 207.200 14.394 3 220.455 195.691 0.000 205.454

1 5 3 123.258 11.102 1 123.258 0.000 0.000 0.000

-4 6 3 93.856 9.688 1 0.000 0.000 93.856 0.000

-2 6 3 99.805 9.990 2 112.216 0.000 87.394 0.000

0 6 3 357.028 18.895 3 375.517 340.541 0.000 355.026

-6 0 4 2367.799 48.660 1 0.000 0.000 0.000 2367.799

0 0 4 4329.891 65.802 1 4329.891 0.000 0.000 0.000

-7 1 4 200.799 14.170 1 0.000 0.000 0.000 200.799

-2 2 4 217.018 14.732 1 217.018 0.000 0.000 0.000

0 2 4 105.860 10.289 1 105.860 0.000 0.000 0.000

-5 3 4 244.042 15.622 2 0.000 0.000 222.637 265.447

-3 3 4 139.302 11.803 1 139.302 0.000 0.000 0.000

-4 4 4 152.583 12.352 2 0.000 0.000 129.004 176.162

0 4 4 56.700 7.530 1 56.700 0.000 0.000 0.000

-3 5 4 1824.118 42.710 1 0.000 0.000 0.000 1824.118

+++++++++++++++++++++++++++++++++++++++++++++++++++++++++++++++++++++++

+ SHELXL-97 - CRYSTAL STRUCTURE REFINEMENT - W95/98/NT/2000 VERSION +

+ Copyright(C) George M. Sheldrick 1993-2001 Release 97-2 +

+ diops_1248 started at 11:47:26 on 24-Jan-2015 +

+++++++++++++++++++++++++++++++++++++++++++++++++++++++++++++++++++++++

TITLE DIOPSID MSPD C2/c (15) DIOPS1+2+4+8 PATTERNS

CELL 0.4246 9.7354 8.9109 5.2451 90. 106.385 90.

ZERR 2 0.0004 0.0006 0.0003 0.000 0.001 0.000

LATT 7

SYMM X,-Y,1/2+Z

SFAC SI O Al Mg Ca Fe

UNIT 8 24 1 3 3 1

V = 436.54 F(000) = 439.0 Mu = 1.33 mm-1 Cell Wt = 884.72 Rho = 3.365

L.S. 10

BOND 0.5

FMAP 2

LIST 5

OMIT -5 7 1

WGHT 0.100000

FVAR 0.30000 0.05 0.9080 0.3019 0.75 0.05

T1 1 0.2874 0.0935 0.22880 11.00000 21

O1 2 0.1140 0.0870 0.13860 11.00000 21

O2 2 0.3652 0.2526 0.32060 11.00000 21

O3 2 0.3517 0.0186 0.99410 11.00000 21

M1Mg 4 10.0000 31 10.25000 50.50000 61

M1Fe 6 10.0000 31 10.25000 -50.50000 61

M2Ca 5 10.0000 41 10.25000 0.45000 0.02

HKLF 4

Covalent radii and connectivity table for E DIOPSID MSPD C2/c (15) DIOPS1+2+4+8 PATTERNS

SI 1.170

O 0.660

AL 1.250

MG 1.600

CA 1.970

FE 1.240

T1 - O2 O1 O3_$12 O3_$14 M2CA_$4 M1MG_$3 M2CA_$17 M2CA

O1 - T1 M1FE_$3 M1MG_$3 M1FE_$16 M1MG_$16 M2CA

O2 - T1 M1FE_$17 M1MG_$17 M2CA_$4

O3 - T1_$1 T1_$5 M2CA_$21 M2CA_$4

M1MG - O2_$2 O2_$9 O1_$3 O1_$6 O1_$10 O1_$11 M1FE_$20 M1MG_$20 M1FE_$18 M1MG_$18 T1_$3 T1_$6

M1FE - O2_$2 O2_$9 O1_$3 O1_$6 O1_$10 O1_$11 M1MG_$20 M1MG_$18 M2CA_$3 M2CA_$19 M2CA_$10

M2CA - O2_$7 O2_$4 O1 O1_$8 O3_$15 O3_$13 O3_$4 O3_$7 T1_$4 T1_$7 M1FE_$3 M1MG_$3

Operators for generating equivalent atoms:

$1 x, y, z+1

$2 x-1/2, y+1/2, z

$3 -x, -y+1, -z

$4 -x+1/2, -y+1/2, -z+1

$5 x, -y, z+1/2

$6 x, -y+1, z+1/2

$7 x-1/2, -y+1/2, z-1/2

$8 -x, y, -z+1/2

$9 -x+1/2, y+1/2, -z+1/2

$10 x, y+1, z

$11 -x, y+1, -z+1/2

$12 x, y, z-1

$13 x-1/2, y+1/2, z-1

$14 x, -y, z-1/2

$15 -x+1/2, y+1/2, -z+3/2

$16 x, y-1, z

$17 x+1/2, y-1/2, z

$18 -x, -y+2, -z

$19 -x, -y+1, -z+1

$20 -x, -y+2, -z+1

$21 x+1/2, y-1/2, z+1

113 Reflections read, of which 0 rejected

-9 =< h =< 8, 0 =< k =< 8, 0 =< l =< 4, Max. 2-theta = 23.31

0 Systematic absence violations

0 Inconsistent equivalents

113 Unique reflections, of which 0 suppressed

R(int) = 0.0000 R(sigma) = 0.0532 Friedel opposites merged

Maximum memory for data reduction = 2164 / 1649

Special position constraints for M1MG

x = 0.0000 z = 0.2500 sof = 0.50000

Input constraints retained (at least in part) for xyz sof and Uij

Special position constraints for M1FE

x = 0.0000 z = 0.2500 sof = 0.50000

Input constraints retained (at least in part) for xyz sof and Uij

Special position constraints for M2CA

x = 0.0000 z = 0.2500 sof = 0.50000

Input constraints retained (at least in part) for xyz and sof

** Cell contents from UNIT instruction and atom list do not agree **

Unit-cell contents from UNIT instruction and atom list resp.

SI 8.00 8.00

O 24.00 24.00

AL 1.00 0.00

MG 3.00 3.00

CA 3.00 3.60

FE 1.00 1.00

Least-squares cycle 1 Maximum vector length = 511 Memory required = 2223 / 45729

wR2 = 0.4688 before cycle 1 for 113 data and 20 / 20 parameters

GooF = S = 5.132; Restrained GooF = 5.132 for 0 restraints

Weight = 1 / [ sigma^2(Fo^2) + ( 0.1000 * P )^2 + 0.00 * P ] where P = ( Max ( Fo^2, 0 ) + 2 * Fc^2 ) / 3

N value esd shift/esd parameter

1 0.18218 0.01598 -7.371 OSF

2 0.01498 0.00804 -4.354 FVAR 2

3 0.90720 0.00272 -0.292 FVAR 3

4 0.29720 0.00196 -2.399 FVAR 4

5 0.81515 0.08803 0.740 FVAR 5

6 0.02553 0.01271 -1.926 FVAR 6

Mean shift/esd = 1.227 Maximum = -7.371 for OSF

Max. shift = 0.046 A for T1 Max. dU =-0.035 for T1

-----------------------------

Least-squares cycle 9 Maximum vector length = 511 Memory required = 2223 / 45729

wR2 = 0.1335 before cycle 9 for 113 data and 20 / 20 parameters

GooF = S = 1.033; Restrained GooF = 1.033 for 0 restraints

Weight = 1 / [ sigma^2(Fo^2) + ( 0.1000 * P )^2 + 0.00 * P ] where P = ( Max ( Fo^2, 0 ) + 2 * Fc^2 ) / 3

N value esd shift/esd parameter

1 0.18264 0.00336 0.000 OSF

2 0.01321 0.00184 0.000 FVAR 2

3 0.90613 0.00075 0.000 FVAR 3

4 0.29520 0.00055 0.000 FVAR 4

5 0.90908 0.02574 0.000 FVAR 5

6 0.01286 0.00350 0.000 FVAR 6

Mean shift/esd = 0.000 Maximum = 0.000 for x T1

Max. shift = 0.000 A for O3 Max. dU = 0.000 for M1MG

Least-squares cycle 10 Maximum vector length = 511 Memory required = 2223 / 45729

wR2 = 0.1335 before cycle 10 for 113 data and 20 / 20 parameters

GooF = S = 1.033; Restrained GooF = 1.033 for 0 restraints

Weight = 1 / [ sigma^2(Fo^2) + ( 0.1000 * P )^2 + 0.00 * P ] where P = ( Max ( Fo^2, 0 ) + 2 * Fc^2 ) / 3

N value esd shift/esd parameter

1 0.18264 0.00336 0.000 OSF

2 0.01321 0.00184 0.000 FVAR 2

3 0.90613 0.00075 0.000 FVAR 3

4 0.29520 0.00055 0.000 FVAR 4

5 0.90908 0.02574 0.000 FVAR 5

6 0.01286 0.00350 0.000 FVAR 6

Mean shift/esd = 0.000 Maximum = 0.000 for x T1

Max. shift = 0.000 A for O2 Max. dU = 0.000 for M1MG

Largest correlation matrix elements

0.885 FVAR 2 / OSF 0.606 z O1 / x O1 0.566 z O2 / x O2

-0.771 FVAR 6 / FVAR 5 0.604 U11 M2CA / sof M2CA 0.552 z T1 / x T1

E DIOPSID MSPD C2/c (15) DIOPS1+2+4+8 PATTERNS

ATOM x y z sof U11 U22 U33 U23 U13 U12 Ueq

T1 0.28981 0.09237 0.23723 1.00000 0.01321

0.01170 0.00057 0.00057 0.00125 0.00000 0.00184

O1 0.11500 0.08887 0.14035 1.00000 0.01321

0.02270 0.00106 0.00120 0.00233 0.00000 0.00184

O2 0.36479 0.24930 0.32559 1.00000 0.01321

0.02200 0.00100 0.00107 0.00240 0.00000 0.00184

O3 0.35086 0.01958 0.99434 1.00000 0.01321

0.02197 0.00103 0.00130 0.00219 0.00000 0.00184

M1MG 0.00000 0.90613 0.25000 0.45454 0.01286

0.00671 0.00000 0.00075 0.00000 0.01287 0.00350

M1FE 0.00000 0.90613 0.25000 0.04546 0.01286

0.00671 0.00000 0.00075 0.00000 0.01287 0.00350

M2CA 0.00000 0.29520 0.25000 0.49821 0.02069

0.00493 0.00000 0.00055 0.00000 0.01138 0.00266

Final Structure Factor Calculation for E DIOPSID MSPD C2/c (15) DIOPS1+2+4+8 PATTERNS

Total number of l.s. parameters = 20 Maximum vector length = 511 Memory required = 2203 / 25039

wR2 = 0.1335 before cycle 11 for 113 data and 0 / 20 parameters

GooF = S = 1.033; Restrained GooF = 1.033 for 0 restraints

Weight = 1 / [ sigma^2(Fo^2) + ( 0.1000 * P )^2 + 0.00 * P ] where P = ( Max ( Fo^2, 0 ) + 2 * Fc^2 ) / 3

R1 = 0.0567 for 99 Fo > 4sig(Fo) and 0.0611 for all 113 data

wR2 = 0.1335, GooF = S = 1.033, Restrained GooF = 1.033 for all data

Occupancy sum of asymmetric unit = 5.00 for non-hydrogen and 0.00 for hydrogen atoms

Analysis of variance for reflections employed in refinement K = Mean[Fo^2] / Mean[Fc^2] for group

Fc/Fc(max) 0.000 0.104 0.130 0.159 0.170 0.214 0.262 0.374 0.452 0.637 1.000

Number in group 12. 11. 11. 12. 11. 11. 12. 11. 11. 11.

GooF 0.619 1.069 0.824 0.794 0.703 1.143 1.031 0.793 1.367 1.607

K 1.166 1.257 1.019 1.013 0.975 1.008 1.015 0.993 1.037 0.980

Resolution(A) 1.05 1.11 1.17 1.23 1.32 1.41 1.48 1.68 1.96 2.50 inf

Number in group 12. 11. 11. 12. 11. 11. 12. 11. 11. 11.

GooF 0.885 1.139 1.069 1.555 0.735 0.937 1.380 0.723 0.840 0.559

K 1.005 0.861 0.927 1.144 1.012 0.918 1.082 1.024 0.952 0.999

R1 0.065 0.085 0.102 0.086 0.041 0.051 0.063 0.043 0.061 0.038

Recommended weighting scheme: WGHT 0.0865 0.0000

Note that in most cases convergence will be faster if fixed weights (e.g. the

default WGHT 0.1) are retained until the refinement is virtually complete, and

only then should the above recommended values be used.

Most Disagreeable Reflections (* if suppressed or used for Rfree)

h k l Fo^2 Fc^2 Delta(F^2)/esd Fc/Fc(max) Resolution(A)

0 0 4 12950.24 9142.45 3.75 0.637 1.26

-6 0 4 7074.67 9652.73 3.02 0.654 1.15

2 4 2 2248.31 1853.65 2.12 0.287 1.49

-5 3 1 15528.30 12813.60 2.10 0.754 1.62

-1 7 1 1468.89 1207.68 2.06 0.231 1.23

1 5 3 359.73 562.57 1.98 0.158 1.18

-4 0 2 10222.30 12368.66 1.95 0.740 2.02

2 6 0 539.59 381.23 1.95 0.130 1.42

0 6 0 13309.97 11184.46 1.90 0.704 1.49

-2 4 2 839.37 641.28 1.90 0.169 1.67

-8 2 1 1528.85 1247.77 1.81 0.235 1.17

0 8 1 479.64 285.35 1.64 0.112 1.09

5 3 1 19245.50 22562.74 1.64 1.000 1.42

-7 3 1 1948.53 1602.11 1.63 0.266 1.26

4 6 0 419.68 259.24 1.50 0.107 1.25

-3 5 4 5455.89 4768.65 1.42 0.460 1.05

4 2 1 779.41 912.96 1.39 0.201 1.76

4 4 0 3777.15 3319.26 1.38 0.384 1.61

-6 0 2 8843.34 10057.09 1.33 0.668 1.53

-2 2 4 629.53 363.86 1.32 0.127 1.26

0 4 3 1918.55 1704.69 1.27 0.275 1.34

3 7 0 119.91 164.09 1.19 0.085 1.18

-7 1 1 1408.94 1070.64 1.18 0.218 1.37

-8 2 3 3177.61 3554.69 1.17 0.397 1.08

6 2 2 779.41 973.37 1.08 0.208 1.14

-4 6 1 929.30 1074.29 1.05 0.218 1.26

1 3 1 329.75 94.61 1.01 0.065 2.38

-4 4 4 449.66 536.20 1.00 0.154 1.10

-5 3 4 719.46 593.62 0.98 0.162 1.12

-1 5 2 269.80 242.95 0.96 0.104 1.47

-2 2 1 22273.22 20385.88 0.96 0.951 2.99

0 6 3 1049.21 843.07 0.96 0.193 1.11

-3 3 4 389.71 636.21 0.93 0.168 1.19

-2 6 2 3387.45 3715.59 0.92 0.406 1.28

-3 1 1 6834.85 7478.99 0.91 0.576 2.90

0 4 2 2967.76 3303.83 0.84 0.383 1.67

1 3 2 1588.80 1720.18 0.83 0.276 1.81

-1 7 2 1229.07 1425.60 0.82 0.251 1.15

0 2 2 3747.18 3412.78 0.79 0.389 2.19

4 2 2 899.32 1119.77 0.78 0.223 1.43

3 5 0 5036.21 4642.38 0.77 0.454 1.55

7 1 0 1708.71 1539.76 0.77 0.261 1.32

-7 1 3 1169.12 1397.70 0.76 0.249 1.21

-2 2 2 419.68 570.73 0.75 0.159 2.20

0 2 1 1558.83 1681.74 0.74 0.273 3.34

5 1 2 1229.07 1347.29 0.74 0.244 1.32

2 2 2 2398.19 2175.45 0.73 0.311 1.82

-6 6 1 569.57 648.96 0.73 0.170 1.10

-1 3 1 7914.04 8506.14 0.72 0.614 2.56

2 4 1 629.53 449.64 0.72 0.141 1.79

Bond lengths and angles

T1 - Distance Angles

O2 1.5849 (0.0112)

O1 1.6332 (0.0129) 117.47 (0.55)

O3_$12 1.6805 (0.0116) 109.27 (0.53) 108.48 (0.71)

O3_$14 1.6477 (0.0146) 105.36 (0.68) 110.28 (0.62) 105.29 (0.44)

M2CA_$4 3.0528 (0.0065) 46.68 (0.44) 131.38 (0.50) 120.13 (0.49) 58.68 (0.48)

M1MG_$3 3.2286 (0.0063) 117.09 (0.47) 32.07 (0.45) 79.54 (0.48) 133.10 (0.46) 156.20 (0.20)

M2CA_$17 3.3356 (0.0061) 117.72 (0.39) 124.79 (0.46) 50.41 (0.39) 54.92 (0.43) 89.31 (0.19) 114.42 (0.18)

M2CA 3.3672 (0.0054) 80.79 (0.36) 37.91 (0.39) 134.41 (0.53) 114.80 (0.43) 99.41 (0.14) 57.55 (0.12) 159.79 (0.20)

T1 - O2 O1 O3_$12 O3_$14 M2CA_$4 M1MG_$3 M2CA_$17

O1 - Distance Angles

T1 1.6332 (0.0129)

M1FE_$3 2.0382 (0.0122) 122.75 (0.58)

M1MG_$3 2.0382 (0.0122) 122.75 (0.58) 0.00 (0.38)

M1FE_$16 2.1446 (0.0113) 120.54 (0.69) 96.06 (0.47) 96.06 (0.47)

M1MG_$16 2.1446 (0.0113) 120.54 (0.69) 96.06 (0.47) 96.06 (0.47) 0.00 (0.23)

M2CA 2.3083 (0.0102) 116.32 (0.61) 93.74 (0.44) 93.74 (0.44) 102.20 (0.35) 102.20 (0.35)

O1 - T1 M1FE_$3 M1MG_$3 M1FE_$16 M1MG_$16

O2 - Distance Angles

T1 1.5849 (0.0112)

M1FE_$17 2.0340 (0.0099) 145.00 (0.58)

M1MG_$17 2.0340 (0.0099) 145.00 (0.58) 0.00 (0.28)

M2CA_$4 2.2787 (0.0122) 102.92 (0.54) 94.74 (0.45) 94.74 (0.45)

O2 - T1 M1FE_$17 M1MG_$17

O3 - Distance Angles

T1_$1 1.6805 (0.0116)

T1_$5 1.6477 (0.0147) 136.98 (0.76)

M2CA_$21 2.6087 (0.0123) 99.83 (0.54) 88.66 (0.55)

M2CA_$4 2.7431 (0.0104) 117.96 (0.50) 95.63 (0.50) 114.21 (0.34)

O3 - T1_$1 T1_$5 M2CA_$21

M1MG - Distance Angles

O2_$2 2.0340 (0.0099)

O2_$9 2.0340 (0.0099) 93.21 (0.51)

O1_$3 2.0382 (0.0122) 90.49 (0.42) 91.24 (0.48)

O1_$6 2.0382 (0.0122) 91.24 (0.48) 90.49 (0.42) 177.49 (0.66)

O1_$10 2.1446 (0.0114) 171.68 (0.43) 93.09 (0.36) 83.94 (0.47) 94.15 (0.44)

O1_$11 2.1446 (0.0114) 93.09 (0.36) 171.68 (0.43) 94.15 (0.44) 83.94 (0.47) 81.19 (0.56)

M1FE_$20 3.1107 (0.0072) 92.94 (0.32) 133.45 (0.36) 134.79 (0.38) 43.28 (0.26) 86.67 (0.38) 40.66 (0.36)

M1MG_$20 3.1107 (0.0072) 92.94 (0.32) 133.45 (0.36) 134.79 (0.38) 43.28 (0.26) 86.67 (0.38) 40.66 (0.36) 0.00

M1FE_$18 3.1107 (0.0072) 133.45 (0.36) 92.94 (0.32) 43.28 (0.26) 134.79 (0.38) 40.66 (0.36) 86.67 (0.38) 114.93 (0.42)

M1MG_$18 3.1107 (0.0072) 133.45 (0.36) 92.94 (0.32) 43.28 (0.26) 134.79 (0.38) 40.66 (0.36) 86.67 (0.38) 114.93 (0.42)

T1_$3 3.2286 (0.0064) 71.82 (0.36) 108.52 (0.37) 25.18 (0.26) 154.80 (0.26) 100.97 (0.38) 78.66 (0.33) 117.21 (0.15)

T1_$6 3.2286 (0.0064) 108.52 (0.37) 71.82 (0.36) 154.80 (0.26) 25.18 (0.26) 78.66 (0.33) 100.97 (0.38) 62.50 (0.09)

M1MG - O2_$2 O2_$9 O1_$3 O1_$6 O1_$10 O1_$11 M1FE_$20

M1FE - Distance Angles

O2_$2 2.0340 (0.0099)

O2_$9 2.0340 (0.0099) 93.21 (0.51)

O1_$3 2.0382 (0.0122) 90.49 (0.42) 91.24 (0.48)

O1_$6 2.0382 (0.0122) 91.24 (0.48) 90.49 (0.42) 177.49 (0.66)

O1_$10 2.1446 (0.0114) 171.68 (0.43) 93.09 (0.36) 83.94 (0.47) 94.15 (0.44)

O1_$11 2.1446 (0.0114) 93.09 (0.36) 171.68 (0.43) 94.15 (0.44) 83.94 (0.47) 81.19 (0.56)

M1MG_$20 3.1107 (0.0072) 92.94 (0.32) 133.45 (0.36) 134.79 (0.38) 43.28 (0.26) 86.67 (0.38) 40.66 (0.36)

M1MG_$18 3.1107 (0.0072) 133.45 (0.36) 92.94 (0.32) 43.28 (0.26) 134.79 (0.38) 40.66 (0.36) 86.67 (0.38) 114.93 (0.42)

M2CA_$3 3.1775 (0.0047) 85.62 (0.32) 45.62 (0.36) 46.46 (0.28) 135.53 (0.32) 94.92 (0.30) 140.50 (0.33) 178.16 (0.32)

M2CA_$19 3.1775 (0.0047) 45.62 (0.36) 85.62 (0.32) 135.53 (0.32) 46.46 (0.28) 140.50 (0.33) 94.92 (0.30) 66.91 (0.13)

M2CA_$10 3.4670 (0.0083) 133.40 (0.25) 133.40 (0.25) 88.75 (0.33) 88.75 (0.33) 40.60 (0.28) 40.60 (0.28) 57.46 (0.21)

M1FE - O2_$2 O2_$9 O1_$3 O1_$6 O1_$10 O1_$11 M1MG_$20

M2CA - Distance Angles

O2_$7 2.2787 (0.0122)

O2_$4 2.2787 (0.0122) 159.96 (0.57)

O1 2.3083 (0.0102) 78.76 (0.40) 85.28 (0.41)

O1_$8 2.3083 (0.0102) 85.28 (0.41) 78.76 (0.40) 74.40 (0.46)

O3_$15 2.6087 (0.0123) 136.05 (0.41) 63.05 (0.35) 119.99 (0.33) 136.15 (0.34)

O3_$13 2.6087 (0.0123) 63.05 (0.35) 136.05 (0.41) 136.15 (0.34) 119.99 (0.34) 79.93 (0.54)

O3_$4 2.7431 (0.0104) 83.36 (0.33) 108.97 (0.33) 90.29 (0.32) 162.47 (0.33) 59.19 (0.15) 65.79 (0.34)

O3_$7 2.7431 (0.0104) 108.97 (0.33) 83.36 (0.33) 162.47 (0.33) 90.29 (0.32) 65.79 (0.34) 59.19 (0.15) 106.02 (0.44)

T1_$4 3.0528 (0.0065) 167.61 (0.29) 30.40 (0.26) 103.25 (0.34) 107.07 (0.30) 32.66 (0.30) 109.81 (0.30) 84.41 (0.28)

T1_$7 3.0528 (0.0065) 30.40 (0.26) 167.61 (0.29) 107.07 (0.30) 103.25 (0.34) 109.81 (0.30) 32.66 (0.30) 72.69 (0.25)

M1FE_$3 3.1775 (0.0047) 39.64 (0.23) 125.00 (0.31) 39.80 (0.31) 82.46 (0.31) 136.67 (0.22) 97.94 (0.22) 80.26 (0.27)

M1MG_$3 3.1775 (0.0047) 39.64 (0.23) 125.00 (0.31) 39.80 (0.31) 82.46 (0.31) 136.67 (0.22) 97.94 (0.22) 80.26 (0.27)

M2CA - O2_$7 O2_$4 O1 O1_$8 O3_$15 O3_$13 O3_$4

FMAP and GRID set by program

FMAP 2 3 8

GRID -5.000 -2 -1 5.000 2 1

R1 = 0.0611 for 113 unique reflections after merging for Fourier

Electron density synthesis with coefficients Fo-Fc

Highest peak 0.63 at 0.2625 0.0957 -0.0089 [ 1.09 A from O3 ]

Deepest hole -0.44 at 0.9429 0.1343 0.1293 [ 1.52 A from O1 ]

Mean = 0.01, Rms deviation from mean = 0.15 e/A^3, Highest memory used = 2357 / 9424

Fourier peaks appended to .res file

x y z sof U Peak Distances to nearest atoms (including symmetry equivalents)

Q1 1 0.2625 0.0957 0.9911 1.00000 0.05 0.63 1.09 O3 1.24 T1 1.82 O1 2.20 T1

Q2 1 0.3282 -0.0798 1.0105 1.00000 0.05 0.50 0.92 O3 1.38 T1 1.88 O2 2.04 T1

Q3 1 0.0428 0.1145 0.0009 1.00000 0.05 0.50 0.89 O1 1.28 M1MG 1.28 M1FE 1.28 M1MG

Q4 1 0.1425 0.2369 0.0384 1.00000 0.05 0.44 1.48 O1 1.89 O2 1.99 T1 2.07 M2CA

Q5 1 0.2500 0.2500 0.5000 0.50000 0.05 0.43 1.63 O2 2.08 T1 2.44 O1 2.45 M2CA

Q6 1 0.4125 -0.0256 1.2506 1.00000 0.05 0.38 1.37 O3 1.56 O3 1.58 T1 1.81 M2CA

Q7 1 0.3313 0.2476 0.0804 1.00000 0.05 0.38 1.23 O2 1.71 T1 2.02 O1 2.10 O3

Q8 1 0.4274 0.0890 1.0096 1.00000 0.05 0.38 0.95 O3 1.99 M2CA 2.03 T1 2.32 T1

Q9 1 0.3825 -0.0361 1.2055 1.00000 0.05 0.37 1.17 O3 1.50 T1 1.64 O3 1.86 M2CA

Q10 1 -0.0794 0.2612 0.2538 1.00000 0.05 0.37 0.84 M2CA 1.70 O1 2.16 O2 2.52 O3

Q11 1 0.4227 0.2607 0.2559 1.00000 0.05 0.32 0.76 O2 1.50 M1MG 1.50 M1FE 1.50 M1MG

Q12 1 -0.0878 0.9183 0.2241 1.00000 0.05 0.31 0.83 M1MG 0.83 M1FE 0.83 M1MG 0.83 M1FE

Q13 1 0.4069 0.2963 0.2482 1.00000 0.05 0.31 0.78 O2 1.33 M1MG 1.33 M1FE 1.33 M1MG

Q14 1 -0.0187 0.3677 0.0500 1.00000 0.05 0.31 1.20 M2CA 1.73 O2 1.82 O3 1.99 O3

Q15 1 -0.0278 0.3197 0.0021 1.00000 0.05 0.29 1.27 M2CA 1.34 O2 2.07 T1 2.13 O3

Q16 1 0.4575 0.2093 0.4629 1.00000 0.05 0.28 1.05 O2 1.45 M2CA 2.02 T1 2.18 M1MG

Q17 1 0.1701 0.0537 0.4661 1.00000 0.05 0.27 1.67 O1 1.74 O1 1.84 O3 1.93 T1

Q18 1 0.1547 0.0908 0.3525 1.00000 0.05 0.26 1.07 O1 1.60 T1 2.09 O3 2.19 M1MG

Q19 1 0.0424 0.0818 0.1810 1.00000 0.05 0.26 0.80 O1 1.68 M1MG 1.68 M1FE 1.68 M1MG

Q20 1 0.2491 0.2444 0.2561 1.00000 0.05 0.22 1.08 O2 1.42 T1 1.88 O1 2.46 M2CA

Shortest distances between peaks (including symmetry equivalents)

6 9 0.33 15 16 0.33 11 13 0.35 14 15 0.49 17 18 0.66 4 7 0.75 14 16 0.82

3 19 0.99 7 13 1.07 2 9 1.08 7 11 1.09 8 14 1.09 12 13 1.10 11 16 1.14

18 19 1.21 19 19 1.24 5 20 1.28 8 15 1.29 4 20 1.31 13 16 1.34 7 20 1.38

2 6 1.38 3 12 1.40 4 10 1.41 11 12 1.41 3 4 1.44 11 15 1.44 6 8 1.44

4 13 1.47 4 11 1.49 10 15 1.50 2 17 1.51 2 14 1.52 10 14 1.52 1 7 1.52

11 11 1.52 8 16 1.53 10 10 1.56 12 19 1.56 6 14 1.57 7 7 1.57 1 8 1.58

1 17 1.59 8 9 1.60 13 20 1.62 10 15 1.62 12 17 1.62 10 16 1.64 10 20 1.64

1 7 1.65 10 19 1.65 12 12 1.65 8 9 1.66 10 14 1.66 2 18 1.66 6 8 1.66

13 15 1.66 3 12 1.66 9 14 1.66 17 19 1.67 12 18 1.67 2 15 1.67 1 2 1.68

10 16 1.68 4 8 1.68 6 14 1.69 2 5 1.69 11 13 1.69 11 20 1.70 6 6 1.70

10 18 1.71 3 13 1.71 7 20 1.72 5 18 1.75 1 12 1.76 3 16 1.76 3 13 1.77

4 15 1.77 2 16 1.78 7 12 1.78 1 4 1.78 9 14 1.78 1 4 1.78 2 8 1.79

4 7 1.79 7 8 1.79 4 16 1.80 3 10 1.80 18 20 1.80 13 13 1.81 1 9 1.81

5 10 1.81 3 11 1.85 9 10 1.85 3 11 1.85 3 7 1.87 3 18 1.87 11 14 1.88

18 19 1.88 8 10 1.88 6 10 1.90 5 17 1.90 1 20 1.91 11 16 1.91 4 12 1.92

6 9 1.95 2 10 1.95 1 20 1.95 3 15 1.95 4 19 1.96 4 14 1.97 3 17 1.99

12 19 1.99 2 20 1.99 6 8 1.99 1 18 1.99 6 15 2.01 1 6 2.01 17 19 2.01

2 6 2.01 8 11 2.01 1 13 2.01 9 15 2.02 12 17 2.02 14 14 2.03 8 14 2.04

16 20 2.04 12 13 2.04 11 15 2.04 3 19 2.05 7 16 2.06 6 14 2.07 4 18 2.08

3 19 2.08 10 19 2.09 6 15 2.09 2 9 2.09 13 16 2.10 9 17 2.10 4 20 2.11

5 16 2.11 11 12 2.11 13 14 2.13 7 10 2.13 8 8 2.15 7 16 2.16 1 3 2.16

7 15 2.16 9 15 2.19 3 17 2.20 9 9 2.20 3 3 2.20 1 9 2.21 12 16 2.22

16 19 2.22 10 17 2.23 3 12 2.23 8 11 2.23 4 11 2.23 6 15 2.24 2 20 2.24

13 15 2.25 5 15 2.25 9 16 2.25 17 19 2.25 4 4 2.25 12 20 2.26 15 20 2.26

1 6 2.27 8 13 2.27 3 18 2.27 17 20 2.27 13 17 2.29 12 19 2.29 1 11 2.29

1 11 2.30 14 15 2.30 7 11 2.31 17 20 2.31 8 9 2.31 5 13 2.32 7 15 2.33

9 14 2.34 6 16 2.34 15 19 2.34 6 16 2.35 19 19 2.35 6 16 2.35 4 5 2.35

7 14 2.36 3 14 2.37 11 14 2.37 3 18 2.37 12 19 2.37 3 20 2.38 9 20 2.38

5 9 2.38 5 11 2.38 4 13 2.39 8 20 2.40 3 10 2.40 6 8 2.41 1 18 2.41

2 8 2.41 19 20 2.42 3 19 2.42 6 17 2.42 16 18 2.43 5 14 2.43 1 13 2.43

2 11 2.43 8 15 2.44 17 18 2.44 3 17 2.46 14 14 2.46 8 9 2.47 9 15 2.48

4 9 2.48 18 20 2.48 12 18 2.49 7 13 2.49 7 13 2.50 15 15 2.50 6 10 2.50

9 18 2.50 9 16 2.51 4 6 2.52 8 8 2.52 2 8 2.52 13 19 2.53 2 6 2.53

16 17 2.53 11 17 2.53 5 7 2.54 6 14 2.54 13 18 2.54 8 13 2.54 6 9 2.54

12 15 2.55 14 16 2.55 8 16 2.55 6 11 2.55 14 20 2.55 4 13 2.55 20 20 2.55

2 14 2.56 9 16 2.57 13 17 2.57 1 10 2.57 6 20 2.58 7 19 2.58 1 14 2.58

16 16 2.59 13 14 2.59 12 16 2.60 9 10 2.60 13 19 2.60 10 11 2.60 7 11 2.60

2 13 2.61 15 18 2.61 1 19 2.61 9 14 2.61 11 19 2.61 3 12 2.62 7 9 2.62

10 11 2.62 8 15 2.62 6 7 2.64 8 14 2.64 16 19 2.64 4 17 2.64 4 11 2.65

8 10 2.65 5 6 2.66 3 8 2.66 14 19 2.66 6 6 2.66 10 18 2.66 13 19 2.67

10 20 2.67 9 11 2.68 1 15 2.68 10 13 2.68 6 15 2.69 5 19 2.69 7 14 2.69

8 16 2.69 9 9 2.70 13 18 2.71 2 7 2.71 4 10 2.71 11 18 2.72 15 19 2.72

7 18 2.72 15 16 2.72 4 17 2.73 10 13 2.73 2 12 2.73 6 6 2.73 2 14 2.73

15 20 2.73 12 18 2.74 7 17 2.74 4 12 2.75 12 17 2.75 14 20 2.75 18 19 2.76

8 17 2.76 1 1 2.77 1 2 2.77 15 17 2.77 9 18 2.79 1 19 2.79 9 15 2.79

11 19 2.79 17 17 2.79 2 10 2.79 8 20 2.81 12 15 2.82 2 9 2.82 2 4 2.82

7 10 2.83 7 8 2.83 2 19 2.83 6 16 2.83 6 18 2.84 3 7 2.84 16 20 2.84

1 16 2.85 14 15 2.85 5 12 2.85 16 20 2.85 1 16 2.85 7 12 2.86 9 20 2.86

7 12 2.87 7 9 2.87 6 13 2.87 15 20 2.87 6 7 2.88 2 15 2.88 7 18 2.89

6 20 2.89 11 19 2.89 1 5 2.89 3 16 2.89 9 17 2.89 1 14 2.90 11 17 2.90

18 18 2.90 6 18 2.90 1 17 2.90 3 18 2.91 14 18 2.91 1 10 2.91 2 10 2.92

14 20 2.93 13 19 2.93 4 9 2.94 2 7 2.94 12 20 2.95 14 19 2.95 4 6 2.96

3 3 2.96 10 12 2.96 9 16 2.96 3 7 2.97 4 19 2.97 7 17 2.97 16 18 2.97

2 4 2.97 9 13 2.97 15 18 2.97 6 17 2.98 8 14 2.98 10 17 2.98 2 2 2.98

4 14 2.99 9 20 2.99 12 12 3.00

Time profile in seconds

-----------------------

0.00: Read and process instructions

0.00: Fit rigid groups

0.00: Interpret restraints etc.

0.00: Generate connectivity array

0.00: Analyse DFIX/DANG restraints

0.00: Analyse SAME/SADI restraints

0.00: Generate CHIV restraints

0.00: Check if bonds in residues restrained

0.00: Generate DELU restraints

0.00: Generate SIMU restraints

0.00: Generate ISOR restraints

0.00: Generate NCSY restraints

0.00: Analyse other restraints etc.

0.02: Read intensity data, sort/merge etc.

0.00: Set up constraints

0.00: OSF, H-atoms from difference map

0.02: Set up l.s. refinement

0.00: Generate idealized H-atoms

0.02: Structure factors and derivatives

0.00: Sum l.s. matrices

0.00: Generate and apply antibumping restraints

0.00: Apply other restraints

0.00: Solve l.s. equations

0.00: Generate HTAB table

0.02: Other dependent quantities, CIF, tables

0.00: Analysis of variance

0.00: Merge reflections for Fourier and .fcf

0.00: Fourier summations

0.00: Peaksearch

0.00: Analyse peaklist

+++++++++++++++++++++++++++++++++++++++++++++++++++++++++++++++++++++++++++++

+ diops_1248 finished at 11:47:26 Total CPU time: 0.1 secs +

+++++++++++++++++++++++++++++++++++++++++++++++++++++++++++++++++++++++++++++

H K L Fo Fc Phase

2 0 0 13.39 13.06 0.00

4 0 0 12.21 14.34 0.00

6 0 0 85.87 85.17 0.00

1 1 0 0.00 1.89 0.00

5 1 0 61.21 59.97 180.00

7 1 0 41.34 39.24 0.00

0 2 0 20.48 21.13 0.00

2 2 0 69.04 69.60 180.00

6 2 0 18.15 15.63 180.00

8 2 0 26.82 24.33 180.00

1 3 0 9.47 9.60 0.00

3 3 0 57.16 58.07 0.00

2 4 0 21.20 18.86 0.00

4 4 0 61.46 57.61 180.00

6 4 0 25.68 25.53 0.00

1 5 0 97.02 96.06 180.00

3 5 0 70.96 68.13 180.00

0 6 0 115.37 105.76 180.00

2 6 0 23.23 19.52 0.00

4 6 0 20.48 16.10 180.00

3 7 0 10.95 12.81 0.00

2 8 0 20.48 18.74 180.00

-7 1 1 37.53 32.72 180.00

-5 1 1 13.41 12.78 0.00

-3 1 1 82.67 86.48 180.00

-1 1 1 9.46 7.88 180.00

3 1 1 78.39 76.45 180.00

7 1 1 26.82 24.80 180.00

-8 2 1 39.09 35.32 0.00

-6 2 1 41.33 42.62 0.00

-4 2 1 65.01 65.03 0.00

-2 2 1 149.24 142.78 0.00

0 2 1 39.48 41.01 180.00

2 2 1 105.60 107.99 0.00

4 2 1 27.92 30.21 0.00

6 2 1 59.72 61.63 0.00

-7 3 1 44.14 40.03 180.00

-5 3 1 124.61 113.19 0.00

-1 3 1 88.96 92.22 0.00

1 3 1 18.11 9.70 180.00

5 3 1 138.73 150.21 0.00

-2 4 1 21.90 20.35 0.00

0 4 1 87.43 89.44 180.00

2 4 1 25.09 21.20 0.00

-1 5 1 24.49 23.70 0.00

3 5 1 25.09 24.80 180.00

5 5 1 14.49 12.31 0.00

-6 6 1 23.86 25.47 0.00

-4 6 1 30.48 32.77 0.00

0 6 1 44.81 46.31 0.00

2 6 1 24.48 23.97 0.00

-1 7 1 38.32 34.75 180.00

1 7 1 51.65 54.04 180.00

0 8 1 21.90 16.89 0.00

-8 0 2 29.48 30.97 180.00

-6 0 2 94.03 100.28 180.00

-4 0 2 101.10 111.21 180.00

-9 1 2 23.87 22.16 0.00

-7 1 2 90.79 92.99 180.00

-5 1 2 31.45 32.50 0.00

5 1 2 35.06 36.70 0.00

-8 2 2 28.45 29.04 0.00

-6 2 2 50.77 50.78 0.00

-4 2 2 17.31 18.41 180.00

-2 2 2 20.48 23.88 0.00

0 2 2 61.21 58.42 0.00

2 2 2 48.97 46.64 0.00

4 2 2 29.99 33.46 180.00

6 2 2 27.92 31.20 0.00

-5 3 2 28.45 28.89 180.00

-1 3 2 55.02 56.14 180.00

1 3 2 39.86 41.47 180.00

-6 4 2 12.24 11.31 0.00

-2 4 2 28.97 25.32 180.00

0 4 2 54.48 57.48 0.00

2 4 2 47.42 43.05 180.00

4 4 2 27.38 28.86 0.00

-7 5 2 82.30 84.70 0.00

-3 5 2 99.00 98.54 0.00

-1 5 2 16.41 15.57 0.00

1 5 2 65.47 63.30 0.00

-2 6 2 58.20 60.96 180.00

0 6 2 96.40 96.57 0.00

4 6 2 21.90 20.22 0.00

-1 7 2 35.06 37.75 180.00

-7 1 3 34.19 37.38 0.00

-5 1 3 22.57 23.81 180.00

-8 2 3 56.37 59.62 180.00

-4 2 3 21.89 20.31 180.00

-7 3 3 22.57 23.09 0.00

-5 3 3 66.83 67.83 180.00

-3 3 3 29.99 29.97 180.00

1 3 3 33.30 30.93 180.00

-6 4 3 35.48 34.90 0.00

-4 4 3 30.97 32.09 0.00

-2 4 3 44.82 43.96 180.00

0 4 3 43.80 41.28 0.00

-5 5 3 30.48 30.16 180.00

-3 5 3 24.49 24.64 0.00

1 5 3 18.97 23.72 180.00

-4 6 3 16.42 17.29 180.00

-2 6 3 16.42 15.78 180.00

0 6 3 32.39 29.03 180.00

-6 0 4 84.11 98.24 0.00

0 0 4 113.79 95.61 0.00

-7 1 4 24.48 24.90 0.00

-2 2 4 25.08 19.07 180.00

0 2 4 17.31 19.39 0.00

-5 3 4 26.82 24.36 0.00

-3 3 4 19.74 25.22 0.00

-4 4 4 21.21 23.16 180.00

0 4 4 12.24 12.82 180.00

-3 5 4 73.86 69.05 180.00
