# Supplementary material for: Application of synchrotron through-the-substrate microdiffraction to crystals in polished thin sections
Source: IUCrJ. 2015 Jun 11;2(Pt 4):452–63. doi: 10.1107/S2052252515007794 (PMC4491317; doi:10.1107/S2052252515007794)
Supplement: Supplementary file 1 [file m-02-00452-sup1.zip › Suppl_files_Rius/Garnet_merging&scaling&LS/Suppl_Garnet.docx]

**Frame-merging for Grossular garnet**

**J. Rius ICMAB_CSIC 2014**

CRYSTAL DATA:

=============

granat_corneana mspd_12DEC2014-14 Ca3Al2Si3O12 Z=8 0.4246Angs

A= 11.847 B= 11.847 C= 11.847

ALPHA= 90.00 BETA= 90.00 GAMMA= 90.00

VOLUME (A3)= 1662.87

(SINT/L)2= 0.001781*H2 + 0.001781*K2 + 0.001781*L2 +

0.000000*HK + 0.000000*HL + 0.000000*KL

BRAVAIS LATTICE IS I CENTRED AT:

1) 0.000000 0.000000 0.000000

2) 0.500000 0.500000 0.500000

LAUE SYMMETRY OPERATIONS:

R11 R12 R13 R21 R22 R23 R31 R32 R33 T1 T2 T3

1) 1 0 0 0 1 0 0 0 1 0.00 0.00 0.00

2) 0 0 1 1 0 0 0 1 0 0.00 0.00 0.00

3) 0 1 0 0 0 1 1 0 0 0.00 0.00 0.00

4) 0 1 0 1 0 0 0 0 -1 0.00 0.00 0.00

5) 1 0 0 0 0 1 0 -1 0 0.00 0.00 0.00

6) 0 0 1 0 1 0 -1 0 0 0.00 0.00 0.00

7) -1 0 0 0 -1 0 0 0 1 0.00 0.00 0.00

8) 0 0 1 -1 0 0 0 -1 0 0.00 0.00 0.00

9) 0 -1 0 0 0 1 -1 0 0 0.00 0.00 0.00

10) 0 -1 0 -1 0 0 0 0 -1 0.00 0.00 0.00

11) -1 0 0 0 0 1 0 1 0 0.00 0.00 0.00

12) 0 0 1 0 -1 0 1 0 0 0.00 0.00 0.00

13) -1 0 0 0 1 0 0 0 -1 0.00 0.00 0.00

14) 0 0 -1 -1 0 0 0 1 0 0.00 0.00 0.00

15) 0 1 0 0 0 -1 -1 0 0 0.00 0.00 0.00

16) 0 1 0 -1 0 0 0 0 1 0.00 0.00 0.00

17) -1 0 0 0 0 -1 0 -1 0 0.00 0.00 0.00

18) 0 0 -1 0 1 0 1 0 0 0.00 0.00 0.00

19) 1 0 0 0 -1 0 0 0 -1 0.00 0.00 0.00

20) 0 0 -1 1 0 0 0 -1 0 0.00 0.00 0.00

21) 0 -1 0 0 0 -1 1 0 0 0.00 0.00 0.00

22) 0 -1 0 1 0 0 0 0 1 0.00 0.00 0.00

23) 1 0 0 0 0 -1 0 1 0 0.00 0.00 0.00

24) 0 0 -1 0 -1 0 -1 0 0 0.00 0.00 0.00

TYPE OF RADIATION IS X-RAYS

UNIT CELL CONTENTS

SYMBOL ATOMIC_NUMBER NUMBER IN CELL TYPE SCAT_POWER

CA 20 24 1 20.00

SI 14 24 2 14.00

AL 13 16 3 13.00

O 8 96 4 8.00

DSMIN OF INPUT REFLECTIONS IS: 1.0815 ANGS

RESIDUAL: 0.0214

------------------------------------------------------------------------------

FACTOR DE ESCALA (F"S) N.PATRO NOM_PATRO

0.9332517 1 GRANAT1_01.HKL

0.9518655 2 GRANAT1_02.HKL

1.0805082 3 GRANAT1_03.HKL

1.0052203 4 GRANAT1_04.HKL

1.0222658 5 GRANAT1_05.HKL

------------------------------------------------------------------------------

MATRIU DE CORRELACIO:

41 32 34 32 22

32 34 32 29 22

34 32 37 33 23

32 29 33 36 25

22 22 23 25 25

------------------------------------------------------------------------------

MATRIU DE RESIDUALS:

0.0000 0.0313 0.0484 0.0468 0.0438

0.0313 0.0000 0.0045 0.0082 0.0074

0.0484 0.0045 0.0000 0.0051 0.0134

0.0468 0.0082 0.0051 0.0000 0.0144

0.0438 0.0074 0.0134 0.0144 0.0000

------------------------------------------------------------------------------

HKL F2AV DF2AV N.CONT F2SCA(IMAG=1), F2SCA(IMAG=2)... FINS NIMAG:

========================================================================

0 2 2 791.078 28.126 4 0.000 814.401 836.255 707.499 806.155

1 2 3 74.119 8.609 1 74.119 0.000 0.000 0.000 0.000

2 3 3 3416.367 58.450 3 3670.243 3781.790 2797.068 0.000 0.000

0 2 4 706.286 26.576 1 706.286 0.000 0.000 0.000 0.000

2 2 4 18548.617 136.193 3 18381.521 17049.852 20214.480 0.000 0.000

1 3 4 4362.835 66.052 5 4705.895 4093.452 3781.378 4569.931 4663.521

0 4 4 1347.965 36.715 4 1490.724 1380.264 0.000 1257.658 1263.214

4 4 4 35049.715 187.216 2 36020.379 34079.055 0.000 0.000 0.000

1 2 5 4503.773 67.110 5 5087.768 4467.777 4172.782 4177.314 4613.227

2 3 5 4528.514 67.294 4 5047.866 4378.755 4290.878 4396.555 0.000

3 4 5 313.004 17.692 3 364.237 320.617 254.158 0.000 0.000

2 5 5 1155.841 33.998 5 1091.651 1204.813 1184.824 1093.862 1204.054

1 1 6 10421.464 102.086 4 0.000 10039.962 10691.752 10095.786 10858.354

0 2 6 3896.200 62.420 5 3939.825 3349.922 4142.711 4063.303 3985.240

3 3 6 574.711 23.973 4 591.125 590.933 540.283 576.502 0.000

0 4 6 32060.355 179.054 5 26432.604 34599.707 36024.410 31588.385 31656.678

2 4 6 36349.195 190.655 5 32218.697 36371.871 36227.824 38645.664 38281.914

1 5 6 434.249 20.839 5 466.918 467.222 394.376 430.639 412.088

3 5 6 629.801 25.096 4 690.633 652.683 593.731 582.156 0.000

5 5 6 277.061 16.645 1 277.061 0.000 0.000 0.000 0.000

4 6 6 17270.711 131.418 4 18453.779 16817.369 16454.051 17357.645 0.000

1 4 7 1419.922 37.682 5 1505.149 1436.061 1397.744 1281.007 1479.647

3 4 7 326.254 18.063 3 382.756 306.117 289.890 0.000 0.000

2 5 7 252.679 15.896 5 287.425 178.374 272.074 273.387 252.134

4 5 7 871.833 29.527 4 1006.201 786.151 949.728 745.250 0.000

1 6 7 682.525 26.125 5 806.669 700.901 616.785 638.707 649.563

5 6 7 650.064 25.496 4 665.918 531.971 734.286 668.081 0.000

2 7 7 777.004 27.875 4 1102.015 0.000 690.307 509.811 805.883

0 0 8 63877.988 252.741 2 0.000 0.000 62425.699 65330.277 0.000

0 2 8 207.763 14.414 2 0.000 0.000 0.000 209.409 206.116

2 2 8 1041.211 32.268 5 954.793 934.963 1062.949 1175.720 1077.630

0 4 8 26237.250 161.979 5 31366.314 25525.496 25506.486 26208.807 22579.143

2 4 8 16206.800 127.306 5 21738.361 15857.389 13729.268 12023.650 17685.336

4 4 8 761.419 27.594 4 816.654 792.819 722.039 714.163 0.000

1 5 8 1031.780 32.121 5 1092.423 1059.536 971.484 1131.709 903.747

3 5 8 1987.565 44.582 5 2054.505 1886.667 2033.892 1946.681 2016.077

4 6 8 12265.148 110.748 3 12356.146 0.000 12412.641 12026.658 0.000

1 2 9 139.798 11.824 1 139.798 0.000 0.000 0.000 0.000

2 3 9 542.076 23.283 5 615.408 432.783 577.771 554.569 529.850

1 4 9 2115.695 45.997 5 2206.324 1863.428 1858.708 2531.235 2118.779

3 4 9 338.019 18.385 5 334.252 345.250 252.331 308.890 449.372

1 6 9 118.498 10.886 2 62.378 0.000 0.000 174.619 0.000

0 2 10 2266.527 47.608 5 2180.628 2017.162 2020.022 2579.492 2535.330

0 4 10 15211.144 123.333 3 16672.605 0.000 13762.764 15198.062 0.000

2 4 10 18044.469 134.330 4 19044.365 0.000 17493.393 16403.229 19236.885

+++++++++++++++++++++++++++++++++++++++++++++++++++++++++++++++++++++++

+ SHELXL-97 - CRYSTAL STRUCTURE REFINEMENT - W95/98/NT/2000 VERSION +

+ Copyright(C) George M. Sheldrick 1993-2001 Release 97-2 +

+ granat1_12345 started at 16:21:23 on 11-Feb-2015 +

+++++++++++++++++++++++++++++++++++++++++++++++++++++++++++++++++++++++

TITLE granat_corneana mspd_12DEC2014-14 Ca3Al2Si3O12 Z=8 0.4246A

CELL 0.4246 11.84730 11.84730 11.84730 90.0000 90.0000 90.0000

ZERR 2 0.0003 0.0003 0.0003 0.000 0.000 0.000

LATT 2

SYMM X+1/2,-Y+1/2,-Z

SYMM -X,Y+1/2,-Z+1/2

SYMM -X+1/2,-Y,Z+1/2

SYMM Y,Z,X

SYMM -Y+1/2,-Z,X+1/2

SYMM Y+1/2,-Z+1/2,-X

SYMM -Y,Z+1/2,-X+1/2

SYMM Z,X,Y

SYMM -Z,X+1/2,-Y+1/2

SYMM -Z+1/2,-X,Y+1/2

SYMM Z+1/2,-X+1/2,-Y

SYMM Y+1/4,X+1/4,Z+1/4

SYMM -Y+3/4,X+3/4,-Z+1/4

SYMM Y+3/4,-X+1/4,-Z+3/4

SYMM -Y+1/4,-X+3/4,Z+3/4

SYMM Z+1/4,Y+1/4,X+1/4

SYMM -Z+1/4,-Y+3/4,X+3/4

SYMM -Z+3/4,Y+3/4,-X+1/4

SYMM Z+3/4,-Y+1/4,-X+3/4

SYMM X+1/4,Z+1/4,Y+1/4

SYMM X+3/4,-Z+1/4,-Y+3/4

SYMM -X+1/4,-Z+3/4,Y+3/4

SYMM -X+3/4,Z+3/4,-Y+1/4

SFAC SI O Al Ca Mg Fe

UNIT 24 96 16 24 1 1

V = 1662.87 F(000) = 1830.0 Mu = 1.50 mm-1 Cell Wt = 3683.92 Rho = 3.679

OMIT 0 2 4

L.S. 10

BOND 0.5

FMAP 2

LIST 5

WGHT 0.100000

FVAR 0.21913 0.95 0.05 0.95 0.05

Si 1 10.25000 10.37500 10.00000 10.25000 0.05

O 2 0.05024 0.63250 0.05209 11.00000 0.05

AlB 3 10.00000 10.00000 10.00000 20.16667 31

FeB 6 10.00000 10.00000 10.00000 -20.16667 31

CaA 4 10.25000 10.12500 10.00000 40.25000 51

FeA 6 10.25000 10.12500 10.00000 -40.25000 51

HKLF 4

Covalent radii and connectivity table for E granat_corneana mspd_12DEC2014-14 Ca3Al2Si3O12 Z=8 0.4246A

SI 1.170

O 0.660

AL 1.250

CA 1.970

MG 1.600

FE 1.240

Si - O_$10 O_$18 O_$19 O_$11 CaA_$24 CaA CaA_$26 CaA_$28 CaA_$27 CaA_$31

O - Si_$5 FeB_$7 AlB_$7 CaA_$24 CaA_$30

AlB - O_$13 O_$12 O_$15 O_$8 O_$7 O_$14 CaA_$29 CaA CaA_$23 CaA_$2 CaA_$25 CaA_$28

FeB - O_$13 O_$12 O_$15 O_$8 O_$7 O_$14 CaA_$29 CaA CaA_$23 CaA_$2 CaA_$25 CaA_$28

CaA - O_$1 O_$8 O_$16 O_$17 O_$19 O_$13 O_$18 O_$9 Si_$1 Si FeB_$21 AlB_$21

Operators for generating equivalent atoms:

$1 -x+1/2, y-1/2, z

$2 y, z, x

$3 -y+1/2, -z, x-1/2

$4 -z+1/2, -x+1/2, -y+1/2

$5 -z, x+1/2, -y+1/2

$6 z, -x+1/2, y-1/2

$7 -x, -y+1/2, z

$8 x, y-1/2, -z

$9 -y+1, -z, -x

$10 -y+1, -z+1/2, x

$11 y-1/2, -z+1/2, -x

$12 -y+1/2, z, -x

$13 y-1/2, -z, x

$14 z, -x, -y+1/2

$15 -z, x, y-1/2

$16 -z+1/4, -y+3/4, x-1/4

$17 z+1/4, -y+3/4, -x+1/4

$18 x+1/4, z+1/4, y-3/4

$19 -x+1/4, z+1/4, -y+3/4

$20 -x+1/2, y, -z

$21 y+1/4, x+1/4, z+1/4

$22 -y+1/4, x+1/4, -z-1/4

$23 -x, -y, -z

$24 -x+1/2, y+1/2, z

$25 -y, -z, -x

$26 -y+1/2, -z+1/2, -x+1/2

$27 y, z+1/2, -x

$28 z, x, y

$29 -z, -x, -y

$30 -z, -x+1, -y

$31 -z+1/2, x, -y

45 Reflections read, of which 1 rejected

0 =< h =< 5, 0 =< k =< 7, 2 =< l =< 10, Max. 2-theta = 22.64

0 Systematic absence violations

0 Inconsistent equivalents

44 Unique reflections, of which 0 suppressed

R(int) = 0.0000 R(sigma) = 0.0067 Friedel opposites merged

Maximum memory for data reduction = 2295 / 763

Special position constraints for Si

x = 0.2500 y = 0.3750 z = 0.0000 sof = 0.25000

Input constraints retained (at least in part) for xyz and sof

Special position constraints for AlB

x = 0.0000 y = 0.0000 z = 0.0000 sof = 0.16667

Input constraints retained (at least in part) for xyz sof and Uij

Special position constraints for FeB

x = 0.0000 y = 0.0000 z = 0.0000 sof = 0.16667

Input constraints retained (at least in part) for xyz sof and Uij

Special position constraints for CaA

x = 0.2500 y = 0.1250 z = 0.0000 sof = 0.25000

Input constraints retained (at least in part) for xyz sof and Uij

** Cell contents from UNIT instruction and atom list do not agree **

Unit-cell contents from UNIT instruction and atom list resp.

SI 24.00 24.00

O 96.00 96.00

AL 16.00 15.20

CA 24.00 22.80

MG 1.00 0.00

FE 1.00 0.80

Least-squares cycle 1 Maximum vector length = 511 Memory required = 2332 / 35334

wR2 = 0.7060 before cycle 1 for 44 data and 10 / 10 parameters

GooF = S = 8.565; Restrained GooF = 8.565 for 0 restraints

Weight = 1 / [ sigma^2(Fo^2) + ( 0.1000 * P )^2 + 0.00 * P ] where P = ( Max ( Fo^2, 0 ) + 2 * Fc^2 ) / 3

N value esd shift/esd parameter

1 0.16753 0.05491 -0.940 OSF

2 0.92640 0.22367 -0.105 FVAR 2

3 0.00094 0.03207 -1.530 FVAR 3

4 0.98219 0.17526 0.184 FVAR 4

5 0.01616 0.02554 -1.325 FVAR 5

Mean shift/esd = 0.802 Maximum = -1.530 for FVAR 3

Max. shift = 0.081 A for O Max. dU =-0.049 for AlB

-------------------------------------------------

Max. shift = 0.000 A for O Max. dU = 0.000 for AlB

Least-squares cycle 10 Maximum vector length = 511 Memory required = 2332 / 35334

wR2 = 0.0874 before cycle 10 for 44 data and 10 / 10 parameters

GooF = S = 0.863; Restrained GooF = 0.863 for 0 restraints

Weight = 1 / [ sigma^2(Fo^2) + ( 0.1000 * P )^2 + 0.00 * P ] where P = ( Max ( Fo^2, 0 ) + 2 * Fc^2 ) / 3

N value esd shift/esd parameter

1 0.17673 0.00386 0.000 OSF

2 0.63913 0.02174 0.000 FVAR 2

3 0.01178 0.00212 0.000 FVAR 3

4 1.02127 0.02520 0.000 FVAR 4

5 0.01486 0.00243 0.000 FVAR 5

Mean shift/esd = 0.000 Maximum = 0.000 for y O

Max. shift = 0.000 A for O Max. dU = 0.000 for Si

Largest correlation matrix elements

0.890 U11 O / OSF 0.734 FVAR 5 / FVAR 3 0.579 FVAR 5 / FVAR 4

-0.805 FVAR 4 / FVAR 2 0.693 U11 O / U11 Si 0.504 FVAR 3 / OSF

0.771 U11 Si / OSF 0.625 U11 Si / FVAR 3

E granat_corneana mspd_12DEC2014-14 Ca3Al2Si3O12 Z=8 0.4246A

ATOM x y z sof U11 U22 U33 U23 U13 U12 Ueq

Si 0.25000 0.37500 0.00000 0.25000 0.01134

0.00000 0.00000 0.00000 0.00000 0.00000 0.00235

O 0.04662 0.65237 0.03797 1.00000 0.01463

0.00794 0.00047 0.00031 0.00040 0.00000 0.00217

AlB 0.00000 0.00000 0.00000 0.10652 0.01178

0.00000 0.00000 0.00000 0.00000 0.00362 0.00212

FeB 0.00000 0.00000 0.00000 0.06015 0.01178

0.00000 0.00000 0.00000 0.00000 0.00362 0.00212

CaA 0.25000 0.12500 0.00000 0.25532 0.01486

0.00000 0.00000 0.00000 0.00000 0.00630 0.00243

Final Structure Factor Calculation for E granat_corneana mspd_12DEC2014-14 Ca3Al2Si3O12 Z=8 0.4246A

Total number of l.s. parameters = 10 Maximum vector length = 511 Memory required = 2322 / 25039

wR2 = 0.0874 before cycle 11 for 44 data and 0 / 10 parameters

GooF = S = 0.863; Restrained GooF = 0.863 for 0 restraints

Weight = 1 / [ sigma^2(Fo^2) + ( 0.1000 * P )^2 + 0.00 * P ] where P = ( Max ( Fo^2, 0 ) + 2 * Fc^2 ) / 3

R1 = 0.0316 for 41 Fo > 4sig(Fo) and 0.0337 for all 44 data

wR2 = 0.0874, GooF = S = 0.863, Restrained GooF = 0.863 for all data

Occupancy sum of asymmetric unit = 1.67 for non-hydrogen and 0.00 for hydrogen atoms

Analysis of variance for reflections employed in refinement K = Mean[Fo^2] / Mean[Fc^2] for group

Fc/Fc(max) 0.000 0.058 0.076 0.098 0.108 0.131 0.174 0.251 0.489 0.594 1.000

Number in group 5. 4. 5. 4. 4. 5. 4. 5. 4. 4.

GooF 0.841 0.767 1.307 0.591 0.948 1.042 0.598 0.678 0.717 0.730

K 0.909 1.022 0.977 1.004 1.008 1.018 1.026 1.063 0.990 0.938

Resolution(A) 1.08 1.15 1.21 1.26 1.29 1.39 1.50 1.64 2.09 2.42 inf

Number in group 5. 5. 4. 4. 4. 5. 4. 6. 3. 4.

GooF 0.920 0.746 1.012 0.660 0.640 0.716 0.932 1.232 0.933 0.365

K 1.061 1.010 0.988 0.906 1.024 0.921 0.978 0.958 1.062 1.008

R1 0.041 0.039 0.041 0.046 0.030 0.043 0.022 0.033 0.033 0.011

** Extinction (EXTI) or solvent water (SWAT) correction may be required **

Recommended weighting scheme: WGHT 0.0636 0.0000

Note that in most cases convergence will be faster if fixed weights (e.g. the

default WGHT 0.1) are retained until the refinement is virtually complete, and

only then should the above recommended values be used.

Most Disagreeable Reflections (* if suppressed or used for Rfree)

h k l Fo^2 Fc^2 Delta(F^2)/esd Fc/Fc(max) Resolution(A)

3 4 5 992.53 1357.47 3.07 0.078 1.68

1 6 9 352.19 745.47 1.86 0.058 1.09

2 5 5 3681.98 4263.81 1.71 0.138 1.61

4 5 7 2785.50 3166.11 1.62 0.119 1.25

0 4 4 4290.31 3785.74 1.41 0.130 2.09

2 3 9 1728.93 1546.65 1.38 0.083 1.22

1 4 7 4514.43 3987.40 1.33 0.133 1.46

4 6 6 55293.78 61175.53 1.31 0.523 1.26

1 5 6 1376.74 1196.01 1.29 0.073 1.50

0 2 10 7235.90 6555.97 1.26 0.171 1.16

1 3 4 13959.52 12742.91 1.22 0.239 2.32

0 0 8 204494.14 223761.19 1.17 1.000 1.48

2 4 10 57759.11 53316.27 1.07 0.488 1.08

4 4 4 112188.42 121771.55 1.06 0.738 1.71

3 4 9 1056.57 1269.26 0.99 0.075 1.15

1 1 6 33361.97 31034.56 0.96 0.372 1.92

4 4 8 2433.31 2268.20 0.92 0.101 1.21

0 4 8 83981.23 78707.27 0.86 0.593 1.32

0 4 10 48698.23 45826.06 0.81 0.453 1.10

3 5 8 6339.41 6770.14 0.80 0.174 1.20

2 5 7 800.43 731.87 0.79 0.057 1.34

1 6 7 2177.17 2310.31 0.73 0.102 1.28

3 4 7 1024.55 902.58 0.73 0.064 1.38

1 4 9 6755.64 6450.00 0.59 0.170 1.20

1 2 3 224.12 164.30 0.59 0.027 3.17

0 2 2 2529.36 2419.38 0.58 0.104 4.19

4 6 8 39253.14 37594.50 0.57 0.410 1.10

2 4 8 51867.93 54076.52 0.55 0.492 1.29

0 4 6 102647.28 106996.34 0.54 0.691 1.64

0 2 6 12454.71 12927.50 0.48 0.240 1.87

0 2 8 640.34 547.14 0.48 0.049 1.44

2 3 5 14471.79 13995.48 0.44 0.250 1.92

2 7 7 2465.33 2567.70 0.40 0.107 1.17

1 5 8 3297.78 3388.81 0.35 0.123 1.25

5 6 7 2081.12 2134.73 0.33 0.098 1.13

1 2 9 416.22 489.46 0.30 0.047 1.28

2 2 8 3329.79 3258.38 0.28 0.121 1.40

2 4 6 116350.66 118695.54 0.26 0.728 1.58

1 2 5 14407.76 14218.88 0.17 0.252 2.16

2 3 3 10917.88 10800.66 0.14 0.220 2.53

3 3 6 1824.98 1808.45 0.09 0.090 1.61

2 2 4 59359.97 59082.30 0.06 0.514 2.42

3 5 6 1985.07 1969.75 0.05 0.094 1.42

5 5 6 864.47 861.84 0.01 0.062 1.28

Bond lengths and angles

Si - Distance Angles

O_$10 1.6450 (0.0045)

O_$18 1.6450 (0.0045) 113.13 (0.16)

O_$19 1.6450 (0.0045) 113.13 (0.16) 102.38 (0.30)

O_$11 1.6450 (0.0045) 102.38 (0.30) 113.13 (0.16) 113.13 (0.16)

CaA_$24 2.9618 51.19 (0.15) 128.81 (0.15) 128.81 (0.15) 51.19 (0.15)

CaA 2.9618 128.81 (0.15) 51.19 (0.15) 51.19 (0.15) 128.81 (0.15) 180.00

CaA_$26 3.6275 35.20 (0.19) 133.86 (0.18) 79.56 (0.16) 107.78 (0.17) 65.91 114.09

CaA_$28 3.6275 133.86 (0.18) 107.78 (0.17) 35.20 (0.19) 79.56 (0.16) 114.09 65.91 99.59

CaA_$27 3.6275 107.78 (0.17) 79.56 (0.16) 133.86 (0.18) 35.20 (0.19) 65.91 114.09 131.81

CaA_$31 3.6275 79.56 (0.16) 35.20 (0.19) 107.78 (0.17) 133.86 (0.18) 114.09 65.91 99.59

Si - O_$10 O_$18 O_$19 O_$11 CaA_$24 CaA CaA_$26

O - Distance Angles

Si_$5 1.6450 (0.0045)

FeB_$7 1.9406 (0.0039) 134.73 (0.31)

AlB_$7 1.9406 (0.0039) 134.73 (0.31) 0.00

CaA_$24 2.4724 (0.0055) 122.24 (0.25) 96.50 (0.18) 96.50 (0.18)

CaA_$30 2.3175 (0.0044) 95.23 (0.18) 101.73 (0.20) 101.73 (0.20) 98.40 (0.18)

O - Si_$5 FeB_$7 AlB_$7 CaA_$24

AlB - Distance Angles

O_$13 1.9406 (0.0039)

O_$12 1.9406 (0.0039) 180.00 (0.41)

O_$15 1.9406 (0.0039) 90.97 (0.21) 89.03 (0.21)

O_$8 1.9406 (0.0039) 90.97 (0.21) 89.03 (0.21) 90.97 (0.21)

O_$7 1.9406 (0.0039) 89.03 (0.21) 90.97 (0.21) 89.03 (0.21) 180.00 (0.28)

O_$14 1.9406 (0.0039) 89.03 (0.21) 90.97 (0.21) 180.00 (0.08) 89.03 (0.21) 90.97 (0.21)

CaA_$29 3.3114 85.41 (0.15) 94.59 (0.15) 132.11 (0.16) 136.74 (0.13) 43.26 (0.13) 47.89 (0.16)

CaA 3.3114 43.26 (0.13) 136.74 (0.13) 94.59 (0.15) 47.89 (0.16) 132.11 (0.16) 85.41 (0.15) 113.58

CaA_$23 3.3114 136.74 (0.13) 43.26 (0.13) 85.41 (0.15) 132.11 (0.16) 47.89 (0.16) 94.59 (0.15) 66.42

CaA_$2 3.3114 47.89 (0.16) 132.11 (0.16) 43.26 (0.13) 94.59 (0.15) 85.41 (0.15) 136.74 (0.13) 113.58

CaA_$25 3.3114 132.11 (0.16) 47.89 (0.16) 136.74 (0.13) 85.41 (0.15) 94.59 (0.15) 43.26 (0.13) 66.42

CaA_$28 3.3114 94.59 (0.15) 85.41 (0.15) 47.89 (0.16) 43.26 (0.13) 136.74 (0.13) 132.11 (0.16) 180.00

AlB - O_$13 O_$12 O_$15 O_$8 O_$7 O_$14 CaA_$29

FeB - Distance Angles

O_$13 1.9406 (0.0039)

O_$12 1.9406 (0.0039) 180.00 (0.41)

O_$15 1.9406 (0.0039) 90.97 (0.21) 89.03 (0.21)

O_$8 1.9406 (0.0039) 90.97 (0.21) 89.03 (0.21) 90.97 (0.21)

O_$7 1.9406 (0.0039) 89.03 (0.21) 90.97 (0.21) 89.03 (0.21) 180.00 (0.28)

O_$14 1.9406 (0.0039) 89.03 (0.21) 90.97 (0.21) 180.00 (0.08) 89.03 (0.21) 90.97 (0.21)

CaA_$29 3.3114 85.41 (0.15) 94.59 (0.15) 132.11 (0.16) 136.74 (0.13) 43.26 (0.13) 47.89 (0.16)

CaA 3.3114 43.26 (0.13) 136.74 (0.13) 94.59 (0.15) 47.89 (0.16) 132.11 (0.16) 85.41 (0.15) 113.58

CaA_$23 3.3114 136.74 (0.13) 43.26 (0.13) 85.41 (0.15) 132.11 (0.16) 47.89 (0.16) 94.59 (0.15) 66.42

CaA_$2 3.3114 47.89 (0.16) 132.11 (0.16) 43.26 (0.13) 94.59 (0.15) 85.41 (0.15) 136.74 (0.13) 113.58

CaA_$25 3.3114 132.11 (0.16) 47.89 (0.16) 136.74 (0.13) 85.41 (0.15) 94.59 (0.15) 43.26 (0.13) 66.42

CaA_$28 3.3114 94.59 (0.15) 85.41 (0.15) 47.89 (0.16) 43.26 (0.13) 136.74 (0.13) 132.11 (0.16) 180.00

FeB - O_$13 O_$12 O_$15 O_$8 O_$7 O_$14 CaA_$29

CaA - Distance Angles

O_$1 2.4724 (0.0055)

O_$8 2.4724 (0.0055) 164.93 (0.17)

O_$16 2.4724 (0.0055) 111.83 (0.22) 70.28 (0.21)

O_$17 2.4724 (0.0055) 70.28 (0.21) 111.83 (0.22) 164.93 (0.17)

O_$19 2.3175 (0.0044) 91.85 (0.14) 75.48 (0.18) 123.52 (0.10) 70.50 (0.19)

O_$13 2.3175 (0.0044) 123.52 (0.10) 70.50 (0.19) 91.85 (0.14) 75.48 (0.18) 117.14 (0.25)

O_$18 2.3175 (0.0044) 75.48 (0.18) 91.85 (0.14) 70.50 (0.19) 123.52 (0.10) 67.16 (0.22) 158.75 (0.24)

O_$9 2.3175 (0.0044) 70.50 (0.19) 123.52 (0.10) 75.48 (0.18) 91.85 (0.14) 158.75 (0.24) 67.16 (0.22) 117.14 (0.25)

Si_$1 2.9618 97.54 (0.08) 97.54 (0.08) 82.46 (0.08) 82.46 (0.08) 146.42 (0.11) 33.58 (0.11) 146.42 (0.11)

Si 2.9618 82.46 (0.08) 82.46 (0.08) 97.54 (0.08) 97.54 (0.08) 33.58 (0.11) 146.42 (0.11) 33.58 (0.11)

FeB_$21 3.3114 77.21 (0.11) 95.98 (0.10) 158.48 (0.09) 35.61 (0.09) 35.02 (0.10) 99.17 (0.14) 94.23 (0.10)

AlB_$21 3.3114 77.21 (0.11) 95.98 (0.10) 158.48 (0.09) 35.61 (0.09) 35.02 (0.10) 99.17 (0.14) 94.23 (0.10)

CaA - O_$1 O_$8 O_$16 O_$17 O_$19 O_$13 O_$18

FMAP and GRID set by program

FMAP 2 3 8

GRID -2.500 -2 -1 2.500 2 1

R1 = 0.0337 for 44 unique reflections after merging for Fourier

Electron density synthesis with coefficients Fo-Fc

Highest peak 0.23 at 0.2486 0.1502 0.0810 [ 1.01 A from CAA ]

Deepest hole -0.24 at 0.3910 0.0587 -0.0012 [ 1.37 A from O ]

Mean = -0.01, Rms deviation from mean = 0.08 e/A^3, Highest memory used = 2409 / 9658

Fourier peaks appended to .res file

x y z sof U Peak Distances to nearest atoms (including symmetry equivalents)

Q1 1 0.2486 0.1502 0.0810 1.00000 0.05 0.23 1.01 CAA 1.65 O 1.73 O 2.33 ALB

Q2 1 0.0596 0.6220 -0.0934 1.00000 0.05 0.23 1.60 O 1.62 O 1.71 CAA 1.95 ALB

Q3 1 -0.0489 0.6033 0.0693 1.00000 0.05 0.20 1.33 O 1.47 O 1.58 ALB 1.58 FEB

Q4 1 0.1289 0.6668 0.0071 1.00000 0.05 0.19 1.05 O 1.52 CAA 2.09 O 2.29 SI

Q5 1 0.0158 0.6582 0.1032 1.00000 0.05 0.17 0.86 O 1.13 SI 1.94 O 2.25 ALB

Q6 1 0.0267 0.6556 0.0736 1.00000 0.05 0.17 0.49 O 1.31 SI 2.06 ALB 2.06 FEB

Q7 1 0.0001 0.5909 0.0254 1.00000 0.05 0.17 0.93 O 1.12 ALB 1.12 FEB 1.12 ALB

Q8 1 0.1618 0.0745 0.0144 1.00000 0.05 0.14 1.22 CAA 1.39 O 1.76 O 2.12 ALB

Q9 1 -0.0170 0.0688 -0.0261 1.00000 0.05 0.14 0.89 ALB 0.89 FEB 0.89 ALB 0.89 FEB

Q10 1 0.2500 0.4805 0.0000 0.50000 0.05 0.14 1.25 SI 1.30 O 1.71 CAA 2.62 O

Q11 1 0.0588 0.7048 0.0447 1.00000 0.05 0.13 0.64 O 1.29 SI 2.10 O 2.19 CAA

Q12 1 0.1018 0.5697 0.0581 1.00000 0.05 0.12 1.20 O 1.62 ALB 1.62 FEB 1.62 ALB

Q13 1 0.0236 0.7222 0.0212 1.00000 0.05 0.12 0.89 O 1.30 SI 1.71 O 1.79 CAA

Q14 1 0.2500 0.0758 0.0000 0.50000 0.05 0.07 0.58 CAA 1.86 O 2.38 SI 2.46 O

Q15 1 0.2645 0.4208 -0.1189 1.00000 0.05 0.06 1.52 SI 1.64 O 1.65 O 2.13 O

Shortest distances between peaks (including symmetry equivalents)

7 9 0.33 1 8 0.35 5 6 0.37 10 13 0.43 11 13 0.54 3 12 0.70 2 8 0.75

3 9 0.76 6 11 0.77 3 7 0.79 5 15 0.86 13 13 0.86 1 2 0.89 2 2 0.91

10 11 0.93 1 4 0.93 9 9 0.94 1 14 1.00 6 13 1.00 6 7 1.01 5 11 1.02

4 11 1.04 8 14 1.06 7 9 1.06 3 5 1.08 11 15 1.08 3 6 1.09 15 15 1.09

9 12 1.10 8 12 1.12 1 12 1.12 10 14 1.13 14 14 1.17 2 4 1.18 4 8 1.19

6 15 1.20 13 14 1.23 7 9 1.23 5 7 1.23 5 13 1.24 6 9 1.28 7 12 1.29

7 12 1.29 9 12 1.29 1 14 1.30 6 10 1.33 7 12 1.33 3 7 1.33 11 13 1.33

4 12 1.34 8 13 1.35 3 9 1.35 7 7 1.36 6 12 1.36 2 2 1.37 3 9 1.39

3 6 1.41 13 15 1.41 3 15 1.42 4 13 1.42 4 14 1.44 3 5 1.44 2 12 1.44

4 15 1.44 4 6 1.45 5 9 1.45 3 15 1.46 9 12 1.46 7 8 1.46 1 1 1.46

5 11 1.46 5 10 1.48 1 1 1.50 4 4 1.52 9 9 1.52 2 8 1.52 3 7 1.53

7 11 1.53 8 10 1.54 2 4 1.54 12 15 1.55 5 12 1.56 5 12 1.56 1 8 1.58

7 13 1.58 10 15 1.59 8 14 1.59 1 13 1.59 8 11 1.59 3 3 1.59 7 9 1.61

2 7 1.62 2 2 1.63 2 8 1.63 5 5 1.64 6 9 1.64 3 12 1.65 2 2 1.65

6 12 1.66 1 2 1.66 8 9 1.66 4 8 1.67 11 14 1.68 6 13 1.68 2 14 1.69

11 12 1.69 6 15 1.69 2 8 1.69 4 8 1.70 1 4 1.71 1 10 1.71 1 2 1.71

8 8 1.72 7 9 1.72 5 9 1.73 11 15 1.74 4 13 1.74 3 13 1.75 5 6 1.75

1 7 1.75 1 2 1.75 11 11 1.76 4 5 1.76 2 7 1.76 6 8 1.76 2 4 1.76

5 13 1.77 1 4 1.77 7 7 1.77 3 15 1.78 3 11 1.78 5 15 1.78 5 13 1.78

4 7 1.78 9 9 1.79 1 8 1.79 2 9 1.80 7 15 1.80 4 11 1.80 4 14 1.80

13 15 1.80 3 8 1.80 5 15 1.80 11 15 1.81 10 15 1.81 1 3 1.82 9 15 1.82

4 10 1.82 7 15 1.82 6 11 1.83 13 15 1.83 3 12 1.83 4 12 1.84 8 13 1.84

12 15 1.84 2 13 1.85 6 7 1.86 9 11 1.86 5 15 1.86 5 10 1.87 3 5 1.87

4 8 1.87 9 13 1.88 4 10 1.88 1 11 1.88 5 7 1.88 7 10 1.89 12 14 1.89

1 11 1.89 5 6 1.89 9 15 1.90 2 11 1.91 1 9 1.92 3 11 1.92 1 1 1.92

3 10 1.92 4 15 1.93 2 14 1.93 13 15 1.93 3 4 1.94 1 13 1.95 2 9 1.96

2 11 1.96 2 4 1.96 2 12 1.96 6 11 1.97 6 15 1.97 1 4 1.97 5 11 1.97

3 11 1.98 3 6 1.98 7 8 1.98 3 8 1.99 7 9 2.00 12 12 2.00 6 15 2.00

8 9 2.00 1 6 2.02 11 12 2.02 8 15 2.02 5 13 2.03 1 7 2.03 1 12 2.03

12 13 2.03 2 4 2.03 1 8 2.04 5 15 2.04 6 6 2.05 2 6 2.05 6 15 2.06

4 14 2.07 1 15 2.07 6 12 2.07 4 5 2.08 2 6 2.08 12 13 2.08 13 15 2.08

1 6 2.08 8 11 2.09 4 9 2.09 8 8 2.10 2 3 2.10 2 7 2.10 12 12 2.10

2 9 2.10 5 8 2.11 14 15 2.11 3 4 2.11 6 14 2.12 8 8 2.12 6 13 2.12

1 9 2.13 4 13 2.13 6 8 2.14 2 12 2.14 2 10 2.14 2 13 2.15 8 8 2.15

4 4 2.15 1 8 2.15 9 10 2.16 10 12 2.16 3 9 2.16 1 13 2.16 3 13 2.17

6 12 2.17 9 12 2.17 5 12 2.17 8 9 2.17 8 12 2.17 1 4 2.18 11 15 2.18

11 11 2.18 2 8 2.18 5 11 2.18 1 2 2.18 3 9 2.19 6 9 2.19 2 9 2.19

6 10 2.19 3 12 2.19 12 15 2.20 5 5 2.21 2 8 2.21 3 3 2.22 7 8 2.22

1 10 2.23 1 3 2.23 7 13 2.23 7 14 2.23 7 7 2.24 4 13 2.24 1 15 2.25

6 7 2.25 12 14 2.26 1 9 2.26 1 13 2.27 9 12 2.27 1 2 2.27 11 11 2.27

4 9 2.27 3 7 2.27 9 11 2.28 15 15 2.28 10 15 2.29 5 6 2.29 11 12 2.29

8 11 2.29 3 11 2.30 10 14 2.30 11 13 2.30 1 5 2.30 7 15 2.31 6 9 2.31

6 13 2.31 11 15 2.31 14 15 2.32 4 7 2.32 2 9 2.32 6 6 2.32 5 12 2.33

8 12 2.33 2 3 2.33 12 15 2.33 5 5 2.33 3 13 2.34 5 9 2.34 1 7 2.34

3 6 2.35 3 4 2.35 11 14 2.35 13 14 2.36 11 13 2.36 2 14 2.36 5 7 2.36

1 2 2.36 4 8 2.36 4 4 2.37 9 12 2.37 10 11 2.37 5 6 2.38 1 11 2.38

12 13 2.38 1 11 2.38 5 14 2.39 2 7 2.39 4 9 2.40 3 7 2.40 5 5 2.40

9 15 2.40 3 13 2.40 6 11 2.40 1 13 2.40 4 15 2.40 2 4 2.41 11 12 2.41

5 8 2.41 1 11 2.41 1 5 2.41 8 9 2.42 11 13 2.42 2 5 2.42 4 6 2.43

1 8 2.43 2 5 2.43 12 13 2.43 5 8 2.43 6 6 2.43 8 13 2.43 6 7 2.44

5 6 2.44 7 12 2.44 1 1 2.44 2 9 2.45 10 11 2.45 12 12 2.45 3 9 2.46

8 13 2.46 7 12 2.46 1 5 2.46 7 12 2.46 9 14 2.47 2 13 2.47 5 7 2.48

12 15 2.48 3 11 2.48 9 13 2.48 4 6 2.48 1 3 2.48 3 14 2.49 2 3 2.49

11 15 2.49 5 9 2.49 3 15 2.49 5 6 2.49 2 13 2.49 9 13 2.50 10 12 2.50

3 13 2.50 7 11 2.50 6 15 2.50 10 10 2.50 1 12 2.51 3 14 2.51 4 13 2.51

8 11 2.51 4 12 2.52 8 15 2.52 10 13 2.52 7 11 2.52 3 5 2.52 1 4 2.52

13 15 2.52 2 11 2.53 7 11 2.53 4 7 2.53 2 10 2.53 13 13 2.53 2 3 2.54

13 13 2.54 8 10 2.54 12 13 2.55 7 8 2.55 5 12 2.55 5 10 2.56 8 9 2.56

8 15 2.56 8 15 2.56 6 8 2.57 2 14 2.57 7 15 2.57 15 15 2.57 5 13 2.57

3 8 2.58 1 12 2.58 2 4 2.58 1 2 2.58 5 9 2.58 5 11 2.58 2 14 2.59

9 11 2.59 3 4 2.59 3 10 2.60 11 15 2.60 9 13 2.61 10 12 2.61 2 15 2.61

6 6 2.61 5 13 2.61 2 8 2.62 8 12 2.62 3 12 2.62 3 7 2.62 6 7 2.63

8 8 2.63 4 10 2.63 2 11 2.63 3 5 2.63 3 15 2.64 4 7 2.64 8 15 2.64

1 6 2.64 8 11 2.64 5 15 2.65 3 10 2.65 4 15 2.65 2 12 2.65 5 7 2.65

2 7 2.65 2 11 2.65 4 4 2.65 4 11 2.65 8 11 2.66 2 15 2.66 5 15 2.66

11 12 2.66 8 12 2.66 1 6 2.66 11 13 2.67 1 15 2.67 2 10 2.67 7 15 2.68

9 15 2.68 7 15 2.69 2 3 2.69 1 10 2.69 2 13 2.70 2 6 2.70 1 1 2.70

9 11 2.70 1 9 2.70 8 12 2.71 3 8 2.71 3 5 2.71 3 10 2.71 7 13 2.71

4 8 2.71 1 3 2.71 6 9 2.71 2 14 2.72 2 8 2.72 6 9 2.72 8 13 2.73

6 15 2.73 4 9 2.73 4 11 2.73 9 15 2.73 3 3 2.74 9 11 2.74 3 11 2.74

11 14 2.74 3 12 2.74 2 7 2.74 2 15 2.74 7 8 2.74 5 7 2.75 4 5 2.75

10 13 2.75 6 7 2.75 13 14 2.75 4 7 2.75 4 6 2.75 9 11 2.75 4 9 2.76

9 11 2.76 4 6 2.76 6 13 2.76 3 15 2.76 7 13 2.76 1 15 2.76

Time profile in seconds

-----------------------

0.02: Read and process instructions

0.00: Fit rigid groups

0.00: Interpret restraints etc.

0.00: Generate connectivity array

0.00: Analyse DFIX/DANG restraints

0.00: Analyse SAME/SADI restraints

0.00: Generate CHIV restraints

0.00: Check if bonds in residues restrained

0.00: Generate DELU restraints

0.00: Generate SIMU restraints

0.00: Generate ISOR restraints

0.00: Generate NCSY restraints

0.00: Analyse other restraints etc.

0.00: Read intensity data, sort/merge etc.

0.00: Set up constraints

0.02: OSF, H-atoms from difference map

0.00: Set up l.s. refinement

0.00: Generate idealized H-atoms

0.00: Structure factors and derivatives

0.00: Sum l.s. matrices

0.00: Generate and apply antibumping restraints

0.00: Apply other restraints

0.00: Solve l.s. equations

0.00: Generate HTAB table

0.02: Other dependent quantities, CIF, tables

0.00: Analysis of variance

0.00: Merge reflections for Fourier and .fcf

0.00: Fourier summations

0.00: Peaksearch

0.02: Analyse peaklist

+++++++++++++++++++++++++++++++++++++++++++++++++++++++++++++++++++++++++++++

+ granat1_12345 finished at 16:21:23 Total CPU time: 0.1 secs +

+++++++++++++++++++++++++++++++++++++++++++++++++++++++++++++++++++++++++++++

**H K L Fo Fc Phase**

0 2 2 50.27 49.16 0.00

1 2 3 14.91 12.76 180.00

2 3 3 104.48 103.92 180.00

2 2 4 243.59 243.02 0.00

1 3 4 118.15 112.88 0.00

0 4 4 65.48 61.51 180.00

4 4 4 334.94 348.95 180.00

1 2 5 120.03 119.24 180.00

2 3 5 120.29 118.30 180.00

3 4 5 31.50 36.84 180.00

2 5 5 60.67 65.29 180.00

1 1 6 182.65 176.16 0.00

0 2 6 111.59 113.69 0.00

3 3 6 42.71 42.51 180.00

0 4 6 320.37 327.08 180.00

2 4 6 341.07 344.49 0.00

1 5 6 37.09 34.57 0.00

3 5 6 44.54 44.37 180.00

5 5 6 29.38 29.34 0.00

4 6 6 235.11 247.29 0.00

1 4 7 67.19 63.15 0.00

3 4 7 32.01 30.04 0.00

2 5 7 28.27 27.04 180.00

4 5 7 52.78 56.27 0.00

1 6 7 46.65 48.06 180.00

5 6 7 45.61 46.19 0.00

2 7 7 49.64 50.66 0.00

0 0 8 452.14 472.96 0.00

0 2 8 25.31 23.39 180.00

2 2 8 57.69 57.06 0.00

0 4 8 289.75 280.51 0.00

2 4 8 227.72 232.52 0.00

4 4 8 49.31 47.60 0.00

1 5 8 57.43 58.21 180.00

3 5 8 79.62 82.28 180.00

4 6 8 198.10 193.87 180.00

1 2 9 20.38 22.10 180.00

2 3 9 41.57 39.31 180.00

1 4 9 82.19 80.31 180.00

3 4 9 32.50 35.63 180.00

1 6 9 18.76 27.29 0.00

0 2 10 85.05 80.96 180.00

0 4 10 220.65 214.05 180.00

2 4 10 240.29 230.86 0.00
